# Supplementary figures and images for: Taxonomic, phylogenetic, and functional diversity of mollusk death assemblages in coral reef and seagrass sediments from two shallow gulfs in Western Cuban Archipelago
Source: PLoS One. 2024 May 14;19(5):e0303539. doi: 10.1371/journal.pone.0303539 (PMC11093297; doi:10.1371/journal.pone.0303539)

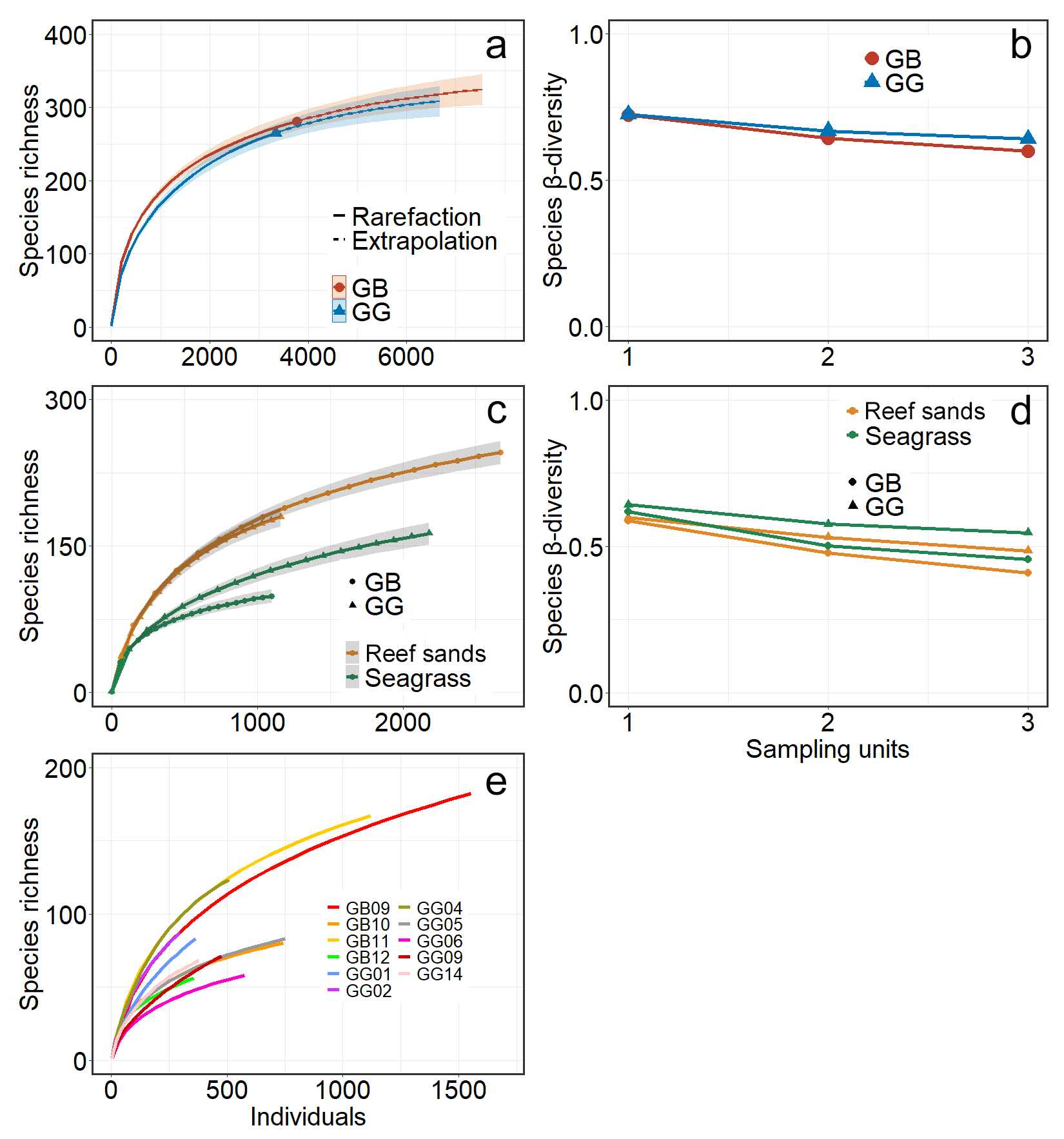

Supplement: S1 Fig — (a) Regional species richness. (b) Regional β-diversity. (c) Habitat species richness. (d) Habitat β-diversity. (e) Local species richness Dots indicate the observed richness, shaded areas the 0.95 CIs. (TIF) [file pone.0303539.s001.tif]

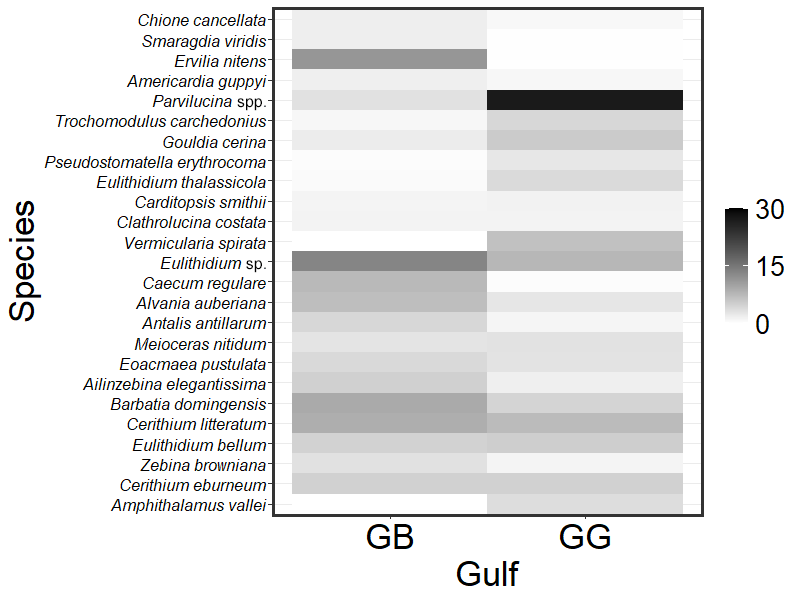

Supplement: S2 Fig — Heat map of relative abundance (in %) of those typical species that contribute to the 70% of similarity within each gulf. Note that species were ordered by their similarity across the gulfs. (TIF) [file pone.0303539.s002.tif]
