# Supplementary material for: Taxonomic, phylogenetic, and functional diversity of mollusk death assemblages in coral reef and seagrass sediments from two shallow gulfs in Western Cuban Archipelago
Source: PLoS One. 2024 May 14;19(5):e0303539. doi: 10.1371/journal.pone.0303539 (PMC11093297; doi:10.1371/journal.pone.0303539)
Supplement: S3 Table — (DOCX) [file pone.0303539.s005.docx]

**Supporting information S5.** Results of the Permutational Analysis of Variance (PERMANOVA) on the multivariate structure of the death mollusks assemblages. Tests correspond to a fully nested design. ECV = estimates of components of variation. In bold those factors statistically significant at p < 0.05.

| Source | Degrees of freedom | Sum of squares | Mean square | Pseudo-F | P-value | ECV (%) |
| --- | --- | --- | --- | --- | --- | --- |
| Gulf | 1 | 7614 | 7613 | 0.66 | 0.82 | 0 |
| **Habitat (Gulf)** | **2** | **23183** | **11592** | **2.78** | **0.0001** | **32** |
| **Site(Habitat(Gulf))** | **7** | **29325** | **4189** | **2.62** | **0.0001** | **30** |
| Residual | 18 | 28776 | 1599 |  |  | 38 |
| Total | 28 | 93837 |  |  |  |  |
