# Supplementary material for: Taxonomic, phylogenetic, and functional diversity of mollusk death assemblages in coral reef and seagrass sediments from two shallow gulfs in Western Cuban Archipelago
Source: PLoS One. 2024 May 14;19(5):e0303539. doi: 10.1371/journal.pone.0303539 (PMC11093297; doi:10.1371/journal.pone.0303539)
Supplement: Attachment — Submitted filename: PONE-D-23-26159_R1_reviewer_20240311.pdf [file pone.0303539.s007.pdf]

PLOS ONE

Taxonomic, phylogenetic, and functional diversity of mollusk death assemblages in coral reef and seagrass sediments from two shallow gulfs in western Cuban archipelago

--Manuscript Draft--

**Manuscript Number:**	PONE-D-23-26159R1
**Article Type:**	Research Article
**Full Title:**	Taxonomic, phylogenetic, and functional diversity of mollusk death assemblages in coral reef and seagrass sediments from two shallow gulfs in western Cuban archipelago
**Short Title:**	Diversity patterns of mollusk death assemblages
**Corresponding Author:**	Rosely Peraza-Escarrá<br>Instituto de Ciencias del Mar y Limnología: Universidad Nacional Autonoma de Mexico<br>Instituto de Ciencias del Mar y Limnologia<br>Coyoacán, CIUDAD DE MÉXICO MEXICO
**Keywords:**	diversity; mollusk death assemblage; thanatocoenosis; Gulf of Mexico; Caribbean Sea
**Abstract:**	Mollusks are recognized for their huge diversity and their shell remnants (death assemblages) provide valuable ecological insights. Diversity patterns in tropical marine habitats still are understudied; therefore, we aimed to investigate the taxonomic, phylogenetic, and functional diversity of mollusk death assemblages at regional and local scales in coral reef sands and seagrass meadows. We collected sediment samples at 11 sites in reef sands and seagrass within two shallow gulfs (Batabanó in the Caribbean Sea and Guanahacabibes in the southeastern Gulf of Mexico). All the shells were counted and identified to species level and classified into biological traits. We identified 7113 individuals belonging to 393 species. Diversity and assemblage structure between gulfs showed many similarities given their geological and geographical commonalities. Reef sands had higher richness than seagrasses likely because of a more favorable productivity-disturbance balance. Reef sands were dominated by epifaunal herbivores feeding on microphytobenthos and bisally attached bivalves adapted to intense hydrodynamic regime. In seagrass meadows, suspensivores dominated in exposed sites and chemosynthetic infaunal bivalves dominated in sites where oxygen replenishment was limited. Time averaging of death assemblages was likely in the order of 100 years in our samples, with stronger effects in reef sands compared to seagrass meadows. Our research provides evidence of the outstanding taxonomic and functional diversity of death mollusk assemblages in tropical coastal sediments as resulted of the influence of scale-related processes and habitat type.
**Order of Authors:**	Rosely Peraza-Escarrá
	Maickel Armenteros, Dr.
	Raúl Fernández-Garcés
	Adolfo Gracia, Dr.
**Response to Reviewers:**	Dear editor and reviewers:<br><br>Thank you very much for the sound evaluation of our paper. We appreciate your suggestions and criticisms very much, we carefully read all of them and accepted the majority. In agreement with reviewer # 2, the paper has been shortened by one third approximately. Also based on suggestions from both reviewers, we removed specific objectives 2 and 3 because of the small number of living mollusks and single deep-sea site, respectively.<br>Below, you can find a detailed response to each point. For easier reading, we have split paragraphs with a hyphen when specific responses are required, and our responses start with R/.

Powered by Editorial Manager® and ProduXion Manager® from Aries Systems Corporation

Looking forward for a positive response for this new version,

Rosely Peraza (on behalf of other authors)

Review Comments to the Author

Please use the space provided to explain your answers to the questions above. You may also include additional comments for the author, including concerns about dual publication, research ethics, or publication ethics. (Please upload your review as an attachment if it exceeds 20,000 characters)

Reviewer #1:
-This ms is well-organized and well-written ms, and addresses some important, mostly still-understudied issues in the application of molluscan dead-shell assemblages to the analysis of marine biodiversity. It includes:
R/ Thank you very much for your opinion, it means a lot to us.

-(1) a spatially-nested analysis of the diversity (several measures) and structure (not fully clear = relative abundance distribution?) of death assemblages; really nice design, and so great to see new field data from this important part of the Caribbean, on top of the general issues;
R/ Thank you. We revised all instances of the use of “community structure” in the manuscript to avoid confusion, if possible, we used a more specific term (e.g., composition).

-(2) an analysis of molluscan death assemblages as a surrogate of the combined dead+living molluscan assemblage; analogous to how benthic foram people approach field samples owing to the challenges of differentiating living and dead individuals, not clear why the authors would do this rather than a more conventional dead versus live comparison, or at least to the exclusion of a more conventional analysis (were sample sizes of living mollusks too small?); and
R/ Indeed, that was the case, living mollusks were too few, then the comparison dead vs live was highly biased because the disproportionate sample size of the two data sets. Based on your comments and the ones by reviewer # 2 about the length of the manuscript, we decided to remove the analysis of dead versus live mollusks (specific objective 2).

-(3) a test and quantification of the extent of down-slope transport of shelf-dwelling species into deep-sea, i.e. slope, sediments; wonderful to have, for comparison with the few other strong tests of this, most notably field data from Bouchet in New Caledonia, and then a zillion anecdotal-quality observations (summarized 30y ago in Kidwell/Bosence).
R/ We agree with reviewer # 2 about that the extent of down-slope transport based on a single deep-sea site is hard to evaluate beyond doubt. Therefore, we removed the specific objective 3.

-The paper thus covers a *lot of intellectual territory and has a rather large number of figures (some could probably go into an appendix, and simply be compared using text to a smaller number of figs published in the main text). But it is well-referenced throughout, connecting the findings well with the large existing literature, both observational and theoretical.
R/ We appreciate and acknowledge your respected opinion. Following your advice, we moved those figures do not convey core information to supplementary material.

- As above, my concerns and suggestions are interspersed below among features that I really like, and were written as I initially read the ms. Overall, I think this would make an excellent paper in Plos-1 and recommend acceptance with only modest revisions, should the editor agree.
Reviewed by Susan Kidwell, thanks for the opportunity.
R/ Thanks again for your encouraging words, your work has inspired us in our studies about mollusk death assemblages.

-Methods = field collection methods were actually better than the ‘usual’, with authors

using box cores rather than van Veens, which are the standard, although, in the end, the depth of penetration of this gear was still only ~10 cm. They thus likely missed collecting many larger-bodied, adult, and especially deeper-burrowing taxa – this is a common problem, should be mentioned either here in Methods or in the Discussion, as a 'caveat', but the authors need not be defensive about it – although it biases the sample, it is an almost universal bias, and thus permits comparison to the many many other, similarly-limited datasets (see Lees 2021 Neth J Sea Res, and Powell &Mann 2016 Cont Shelf Res for discussions). I especially admire that they acquired these samples on SCUBA, allowing for careful ad known placement… and 3 replicates per site.
R/ Agree. We added the following sentence in the Sampling section: "A potential caveat with this sampling is that it may miss deep burrowing species inhabiting below 10 cm deep".

- Description of field study areas is exemplary – these authors really describe the areas in useful ways, including extent of physical disturbance and, of course, likely human effects, drawing on earlier work (includes the admirable Table 1)… thus well-chose field areas. The 0.5mm sieve is much finer than is usual (1mm, and in some live-dead studies by geologists/paleoecologists, a 2mm or coarser sieve is used) – only a concern because this likely included juvenile individuals that can be hard to identify AND have unlikely persistence, both as living individuals (persistence to adulthood) and as dead shells (postmortem) – although I appreciate that tropical faunas can have many many 'micromollusks' that might be largely missed with a 1 mm mesh. Just something worth mentioning, as a guide to other researchers thinking of repeating this kind of analysis elsewhere.
R/ Full agreement, we chose 0.5-mm mesh size based on previous reports about the huge diversity of micro-mollusks in Cuban waters, as there are many new species described by Espinosa et al. from both studied gulfs.

-Live data have already been reported, in Armenteros et al 2018 – those results should be provided in tabular form here because the sample sizes of this living assemblage is critical to evaluating live-dead agreement.
R/ Noted. We removed objective 2 from the paper, therefore the data from Armenteros et al. are not needed any more in the paper.

-Analysis – Richness: I really like the authors' approach for taxonomic richness, strongly prefer accumulation curves rather than post-hoc rarefaction fittings, AND I like using effective numbers of species for richness – super. Phylo richness – not familiar, feels like a stretch to add this, but no objection. Functional richness – good use of Todd & Mikkelson/Bieler for coding taxa; although the N of categories is (slightly) smaller than the S of species, could still use the same metrics; but I like bringing in these FD metrics from biogeography/macroecology. Beta – many ways to do it, they should also consider (and in discussion compare their results to) the methods used in Tomasovych/Kidwell 2009-2011 (e.g., diversity transfer from alpha to beta, spatial gradient capture, richness/evenness at alpha) – a series of papers by those authors (summarized in Kidwell Tomasovych 2013) explore the effects of time-averaging on the suite of metrics used here, seems to be one part of the paleo/taph literature missed.
R/ We agree that there are many ways to approach this sort of data. This is in part the reason for a paragraph of the introduction devoted to the most relevant (in our opinion) and modern techniques for analysis of diversity. We do not wish to enlarge much the manuscript with another set of metrics and prefer to keep it as it is. Indeed, reviewer # 2 is requesting to shorten the manuscript's length.

-Evenness – would prefer to see a quantitative metric of evenness, such as the sample-size-independent Hurlbert's PIE, rather than only a graphical depiction; N singletons and doubletons is a measure of the proportion of rare taxa, which is useful/important, but not the same as dominance/evenness that focus on the proportional abundances of the top few taxa and overall shape of the cumulative richness curve. PIE is now widely used in live-dead analysis.
R/ We disagree. We prefer to use graphical devices (dominance curves) instead of reducing all the information to a single index.

-Ah—structure = simper analysis, not what I'd call structure, but rather inter-sample

Powered by Editorial Manager® and ProduXion Manager® from Aries Systems Corporation

similarities, series of pairwise comparisons…
R/ Agree. We change the term “structure” by composition, which is clearer.

-Comparison of live-dead – using the ancient, very simple metrics of Kidwell & Bosence 1991. That’s OK as long as include a table with the raw N’s being compared (e.g., how similar is the N of living and dead individuals?), because those ancient authors found that the small-sample size of the living assemblages in most studies was the most important factor explaining (correlated with) the low apparent live-dead agreements (which the later modeling of Kidwell and others showed was true). But a full live-dead nowadays would explore live-dead using standard ecological measures such as (dis)similarity of paired live-dead species lists for a site/habitat/region, ditto in rank-abundance of taxa live&dead, nmds etc. [I do note a NMDS lines469; another use of the word ‘structure’, here meant to indicate *spatial variation in species composition of assemblages, whereas structure also used to indicate RADs… be careful, would be good to sharpen language. **I fear that the omission of such measures owes to very low abundances of living individuals, an especially large challenge in tropical settings in my experience… typically we have to process far more sediment for living than for dead to achieve statistical power (see Powell et al from the early 1980s for the first time this was recognized and acted upon; Gilad et al 2018 did the same thing, sieving many-x more volume of sediment for live than dead, in tropical shelf habitats for the same reason). If this is indeed the situation, the authors, again, do not need to be defensive but simply straightforward and ‘up-front’, that is explain from the start that there are limits to what they can do analytically given extremely disparate live-dead sample sizes (and probably extremely small Ns of living in absolute terms, e.g. anything <50 inds and especially <20 inds).
R/ We removed the comparison live-dead, which corresponds to objective 2, because the very low abundance of living individuals, for most of the sites, abundance was < 20 individuals. We changed the term “structure”.

-Results – first para on general features is where the authors should immediately start differentiating their living and death assemblages – e.g., of these 7113 inds, how many dead & how many alive, ditto the S of species orders etc. If all of these numbers and others in the Results section are going to refer exclusively to the death assemblages, then this also needs to be made explicitly clear here, e.g. “All results refer only to dead-shell assemblages unless otherwise noted (e.g., section xx on dead vs total comparison).” I find it hard to proceed without knowing this basic information.
R/ We agree and follow your advice, so in the first paragraph of the results we mention that we identified 7113 dead individuals.

-Line 333 – ‘fluctuations’ typically would refer to variability in time, whereas here I think the authors mean variability among spatial replicates at a site etc collected during the same season… watch for this.
R/ Agree. We changed fluctuation × variability.

-Line 514 Table 2 – Finally! Info on N live and N dead… and indeed the disparity is super high, not shocking for a tropical shallow marine system but very troubling for live-dead comparison. My suspicion re why the authors chose to compare dead versus live+dead are confirmed. This is not a scientifically fair/appropriate test of the ability of the dead to serve as a surrogate of the usual, entirely-live-collected kind of data that biologists typically have – and moreover flips the usual disparity (usually the ‘whole’ is much larger/broader/more inclusive sampling of the target than is the subset being tests as a ‘surrogate’/proxy of i). The results are of course interesting, but mostly for enlightening readers about the challenges of live-dead analysis in such settings. I think the discussion and conclusions from this section will thus need to be very modest, very modulated. ** For the editor: The disparities are e.g. 1500 dead inds versus 5 living ones, etc, with only two sites yielding >20 individuals, with 20 being an absolute minimum N of individuals to do any kind of nmds etc.
R/ Agree. We acknowledge that the disparity between the two kind of assemblages is too large for a fair comparison, and most of the sites has < 20 alive individuals. Thus, we removed from the paper the objective 2 about comparison live-death, that necessarily implies to change the title. However, we moved the information about the abundance and observed richness of dead individuals to the table 1.

-In this table 2, I’m most intrigued by the (to me) large number of taxa found exclusively

	alive, i.e. "alive-only" – I would not expect this given the huge disparity in sample size, i.e. that any taxa would not be encountered among the dead. 2 explanations = (1) new, invasive taxa, populations still small, little opportunity for dead shells to accrue to measurable levels in the death assemblage [given the species, I doubt this, but authors should (in the discussion) indicate if these are all natives or otherwise commonly encountered already by others in Cuba; or (2) taphonomic bias, i.e. these are all taxa with truly exceptionally low preservation potential, e.g. from their especially small/thin-shelled bodies, and/or super-rarity, and/or high-organic aragonitic shell types, and/or life-habit (e.g. I find that commensals have very low preservation potential). From my experience, Solemya has a super-low pres pot (as do lyonsiids, pinnids, some pandoriids), and I commonly find that Bittium, Caecum, and Rissoids/small parasitic & minute herbivores are strongly underrepresented in the dead (but not actually absent) Limaria and Americardia & Codakia should definitely not fall into this category… anyway, a surprising and super-interesting result<br>R/ We removed this part of the paper, although we added the abundance and number of observed species of dead mollusks in the table 1, as basic numbers for diversity assessment and sample size information.<br><br>-523 contribution of shelf species to deep-sea death assemblages – wonderful, super useful observations. It would be super-helpful to provide the typical or maximum known body sizes of these taxa found off shelf, and/or, even better, the typical or max body sizes observed among thte individuals actually collected. Would make a more powerful discussion/conclusions<br>R/ These observations are valuable, but as indicated by reviewer # 2, the occurrence of a single deep-sea site (with only data of presence/absence) is a main weakness of this part. We preferred to remove objective 3 from the paper.<br><br>-Table 3 this section – Please provide info at the bottom of each column on the total N of the death assemblage so that readers can get full value from these prop abundance values. E.g. does 100% ("1") mean 10 out of 10, 100 out of 100 inds, or 1 out of 1? The data on raw N of dead shells from a tropical slope would be valuable in itself.<br>=Interesting from a taphonomic perspective that all are gastropod species w one exception… because epifaunal, which is long thought to increase the potential for postmortem transport (because they die above the sediment-water interface—idea probably in KidwellBosence)? Some info on life habit, not just gastro-bivalve class membership, would be nice to add to this table, or at least to bring into discussion.<br>R/ We removed table 3 from the paper.<br><br>Discussion –<br><br>-549 hyper diverse, may never reach an asymptote – probably true, although still not a popular idea, but that's because most people have never encountered the tropics in a first-hand way. Thus I am glad to see you say this, is a perfectly good description of your curves!<br>R/ Thanks!<br><br>-Regional scale – agree w your interpretation<br>579 – again, people generally don't like to talk about high productivity and coral reefs, or the role of upwelling, but am sure this is a factor in a lot of places and here you have independent evidence of upwelling to promote it; nice regional insight<br>R/ Thanks again!<br><br>-614 – yes Kidwell/Bosence for the even-ing effects of time-averaging (also see the older Peterson paper they cite), but there's more explicit, rigorous testing of this effect in younger literature, e.g. Olszewski/Kidwell 2007, var Tomasovych/Kidwell (it's also in SK/ATomas 2013).<br>R/ We removed this part of the manuscript.<br><br>-620 habitat – by the way, when I first saw 'coral reef' in the abstract, I assumed this would be an analysis of mollusks in the reef framework itself (with all of those huge challenges, e.g. see many nice papers by Zuschin). So I was happy/relieved to see that the authors actually meant the sandy seabeds between reef tongues, i.e. the grooves… be sure that this is clear in the abstract and intro, and then again remind reader in the discussion. Lots of potential for shells washed in from adjacent hard

Powered by Editorial Manager® and ProduXion Manager® from Aries Systems Corporation

substrata/reef framework, but also plenty of taxa that live (and die) in the sands themselves.
R/ Agree. We made clear in the introduction that we are analyzing shells from sedimentary environments within each habitat type. Indeed, we change coral reef × reef sand across the manuscript to make clear that we deal here with sediment assemblages (and no hard-bottom assemblages).

-640ll – yes, transport *can be important, but commonly less that folks worry about it being; thanks for having read the SK/Bosence paper carefully enough to realize that we concluded it was a relatively minor issue in most level-bottom subtidal settings (ditto even on steep shelves per SK 2008, right!).
R/ Agree.

-657 – indeed, it *is interesting that susp feeders are as unimportant as they are in these sandy inter-reef areas oh, that's a term you could use, "inter-reef sand" rather than "coral reef"
R/ Agree. We changed the term for "reef sands".

-To 672 – I really love all the detailed info and thinking about the natural history of your settings, it's a usefully thoughtful discussion.
R/ Thank you very much.

-687 – evenness curves – OK, not crazy about this approach.
R/ Dominance curves are a powerful and widely used technique to detect environmental disturbance and grossly inform about how many species are dominant, moderately dominant, and rare in the assemblages. Indeed, these curves indicated in our study the two sites subjected to local impact.

-693 – 'substantial inter-replicate variability, i.e. small-scale spatial heterogeneity – I find this, too, in tropical shelves… the world is patchier than we imagine, even with time-averaging. Am glad that the authors are being 'true', i.e. fully honest about their findings, are not trying to smooth it over.
R/ Thanks.

-Tomaso et al 2023 – no need to be defensive, this is an 'ideal' work plan, very difficult and expensive to achieve! BTW Adam and I try very hard to get other workers to appreciate 'time averaging' as a scaling process, which operates post-mortem, but NOT as a taphonomic process the way people typically mean that. T-avg does not 'bias' the record, but rather simply averages the input of shells over time, with a series of predictable effects (as summarized SK/AT 2013). It provides a window of opportunity for taph processes such as transport, fragmentation, bioerosion, etc to genuinely bias the composition of that time-averaged assemblage. But time averaging simply means we're sampling bio over a period of time longer than the typical snap-shot of a biological survey – if one were to sum many live-collections over time, the effects onn richness, evenness would be the same, *unless one biased that assemblage by e.g. removing many of the fragile-shelled species. Sorry to be this pushy, but the authors are careful thinkers and ambitious, so it would be great to have the time-averaging vs taphonomic-bias language sharpened in this ms.
R/ We agree with this approach considering time-averaging as a process through the time with predictable outcomes (e.g., increase the richness) instead of a bias. We added a sentence in the introduction to shift away the approach from bias to a time-integrated process. Also, we created a final section in the discussion to discuss the effects of time averaging on our diversity outcomes.

-710—ah, Table 2 discussion! Ok, I see your very good discussion, thanks.
R/ Thanks.

-737 – delighted to see this thoughtful discussion of time averaging, AND that the authors proceed to think-through their local situation given the 210Pb data at hand. Logical, fine job.
R/ We relabeled this part as a subsection in the discussion. Thanks for the encouraging opinion!!

-757 – and dead-only treatment, good. I think they're completely right… although

Kidwell/Bosence is really old, we hit upon a fundamental truth
R/ We removed this part since our evidence is rather weak given the small number of alive individuals we could analyze.

Shelf-deep-sea spp –

-I wondered about changing “deep-sea” to “slope”, throughout the ms, but 1500m is a really deep part of a slope, so just leave it alone. Nice discussion.
R/ Thanks. Unfortunately, we had only a single deep-sea site, and we do not have enough evidence to support any of the three ad hoc explanations. In face of such uncertainty, we prefer to remove the analysis of shell transport between shelf and deep sea.

-Congratulations on a really nice piece of work – a *lot of fieldwork, a *lot of lab work to pick/sort/count these dead individuals down to such a small body size… and then looking at so many metrics including some still-novel ones for live-dead analysis, e.g. FD and Phylo-D.
R/ Thank you very much for your wise advices and careful revision.

With best wishes, SK

Reviewer #2:

-The manuscript " Diversity patterns of mollusk death assemblages in coral reefs and seagrass meadows and a comparison with living assemblages." authored by Peraza-Escarrá and collaborators evaluated the diversity patterns of mollusks assemblages in two gulfs and two different habitats. The main questions of the study are to (1) investigate the taxonomic, phylogenetic, and functional diversities of mollusk death assemblages at regional and local scales in 19 coral reefs and seagrass meadows; (2) evaluate death assemblages as surrogates of the whole mollusk assemblages (alive + dead); and (3) explore the downslope transport of shelf species to the deep sea. The manuscript is relatively well-written but excessively lengthy. The topic is very interesting to the scientific community, but some issues should be addressed, and missing information should be included before publication.
R/ Thanks to the reviewer for the careful revision of our paper. We will check point by point your recommendations and criticisms.

-My main concerns are about how the manuscript is organized, it gives the impression of being organized more like a chapter of a Ph.D. thesis than a scientific article, including too many details often irrelevant to the manuscript's fundamental topic. The main goal is confusing since the title highlights the “mollusk death assemblages in coral reefs and seagrass meadows and a comparison with living assemblages” and there is no specific development of this idea/topic. For example, different analyses with alive and death assemblages separately could be interesting for differentiating them and could be interesting in terms of current ecological differences between seagrass meadows and coral reef mollusks.
R/ We agree with the reviewer’s criticism regarding the length of the manuscript. We also have in account the remarks by reviewer # 1 about the small sample size of the living mollusk database. Therefore, we removed the specific objectives 2 and 3, that contribute together about 1/3 to the whole paper and are based on rather limited evidence due to small sample size (objective 2, living assemblages) and reduced spatial coverage with single deep-sea site (objective 3, shelf-slope transport of shells). We note that given the large amount of information contained in objective 1, the paper still has 2/3 of the original length/content.

-Finally, for the proposed experimental design is also necessary to provide more specific details that are not yet included.
R/ We provided more details in this section.

-The paper in its current form needs major revision before publication. Below I provide some suggestions to improve the manuscript.
R/ Thanks. We addressed all the suggestions.

Specific comments

Powered by Editorial Manager® and ProduXion Manager® from Aries Systems Corporation

-The authors should carefully review and choose the article's topic and then select the important and relevant information on that topic (seagrass meadows vs coral reef?), discarding the rest. In its current form, I consider the manuscript to be too lengthy, containing a wealth of information without a clear guiding thread. On the other hand, it appears to be a mix of data and interpretations in which, at times, it is difficult to find a common thread. Once the authors focus on the objective of what they want to convey in the article, they should direct the discussion to that topic. It's worth noting that the bulk of the work has already been done, as this should only involve a readjustment and reorganization of content.
R/ We agree. As stated above, we removed objectives 2 and 3 and focused on the diversity patterns of dead assemblages.

-A thorough review of the English is necessary to correct mistakes.
R/ We revised the English usage.

-Abstract. Authors should integrate better the sentences, sometimes seems to be a succession of sentences without links between them.
R/ Agree. We revised the abstract.

Introduction

-The authors should rearrange the introduction. I consider it very important to include information about coral reefs and seagrass meadows in the ocean and specifically the mollusk assemblages in those habitats, and why is very important to study them.
R/ Agree. We added a couple of sentences about the two habitats in the introduction, including a statement about the specific biotope sampled (sediments).
-Authors should reorganize the introduction from the most general to the most specific.
R/ Agree. Introduction covers some topics that necessarily must be started de novo, for instance, dead assemblages, habitat, diversity, and Cuba (regional and local settings). However, we improved the connection between paragraphs, keeping the order from general to specific.

Material and Methods
-Why isn't there the same number of replicates in all cases? Because in the case of GB, we have two seagrass sites and two coral reef sites, but in the case of GG, there are 3 and 3? Why wasn't the same number of replicates (n=3) maintained in all cases? Justify it.
R/ The number of replicates (box cores) was n = 3 for eight out of 11 sites, only in three sites we could not collect 3 replicates because diving time was not enough for it. We note that the number of studied sites was different because of limits imposed by the distance of navigation, and amount of material to be processed/identified. We believe that even with this unbalanced setting, our data is valuable and robust.

-Give more details of the deep-sea sampling.
R/ As suggested by the reviewer, this part of the manuscript was removed.

-Is a single slope replicate sufficient to address the differences between the communities of the platform and the slope? Shouldn't the study be balanced? Justify it because this fact could compromise the entire comparison.
R/ Agree. The evidence here is rather limited due to a single deep-sea site being studied. Therefore, we removed this part from the manuscript.

-Authors should also consider to conduct multivariate analyses such as PERMANOVA that assess the entire community and separately for living and dead mollusk assemblages.
R/ The disparate sample size between dead and living assemblages is a main shortage for a fair comparison. Therefore, we removed the section of the manuscript related to the living assemblages. However, we made a PERMANOVA for complementing the results of the assemblage structure because in this case we can use the replicates in the statistical analysis.

-It should be considered by the authors to give more details of the experimental design used, specifying the fixed factors, random factors, nested and orthogonal factors.

R/ Agree. More information was added to the manuscript.

Results

-At times, it is stated that there are significant differences between certain factors. What statistical tests have been conducted to assert the existence of such differences?
R/ …In the manuscript, subsection of data analysis we stated: “In general, the criterion for testing differences between groups was the overlapping of the 0.95 CIs calculated on basis of permutations”. We note here that for the analysis of richness, the most appropriate approach is to sum all the replicates of the sample, it means that no replication exists for making a traditional test such as analysis of variance. In this case, the way to generate a statistical device for the comparison is the calculation of confidence intervals (CIs) based on permutations and to use, as criterion of significance, the overlapping (or not) of the 0.95 CIs.

-The title refers to the comparison of living mollusks with the dead ones, but this comparison is not explicitly addressed except in a small section discussing percentages. Furthermore, throughout the manuscript, there is no mention of a separate matrix that differentiates these two distinct communities
R/ We agree. We removed the part of the comparison between living and dead, and modified the title in consequence.

-Some parts of the results are redundant and some are unnecessary (depending on the topic of the manuscript); for instance, the information provided in Figures 4 and 7 is essentially the same. It would make more biological sense to treat it separately by habitats as it appears in Fig 7 (seagrass and coral reefs) according to the indicated in the title of the work, rather than jointly in GB and GG. The authors should review this idea and provide only the necessary results for the study's topic.
R/ We disagree. The spatial scale is a very important issue in diversity studies; therefore, we consider it very important to describe the regional (i.e., between gulfs) and local (i.e., between sites) patterns.

-Perhaps it's a good idea to analyze the data in a more general manner without delving into details of differences between replicates and specific gulfs. If the goal is to contribute to the broader scientific community, it might be more important to focus on observable differences in mollusk assemblages between habitats (seagrass vs. coral reef). This approach could involve explaining differences in species number, abundance, and functional traits rather than detailing local differences between the two. With this shift, the almost exclusive local focus of the study can be broadened to a much more global interest
R/ We disagree. As stated above, the comparison at two spatial scales is relevant to current biodiversity research. The two large spatial units compared here (GG and GB) belong to two biomes of paramount importance in the tropical Atlantic Ocean: Gulf of Mexico and Caribbean Sea, respectively. In our opinion, there is no way that the comparison between these units be local or not interesting. This sort of comparison is the foundation of biogeography, and we believe they must be analyzed in the paper as is. In addition, to analyze diversity at local scale allows to disentangle the role of local processes on the difference at level of habitat. Indeed, the nested analysis indicates that the local differences are very important to explain the large variability within the same habitat.

Discussion

-Considering the aforementioned points, it might be advantageous to reframe the discussion. Instead of delving into intricate details of differences between replicas and specific gulf regions, a more effective approach could be to analyze the data in a broader context. Focusing on the overarching differences in mollusk assemblages between habitats, specifically comparing seagrass and coral reef environments, would align better with a global scientific community. This approach would involve elucidating variations in species number, abundance, and functional traits, steering away from intricate local disparities. By adopting this perspective, the study's interest could transition from predominantly local to a more globally significant contribution
R/ This comment is in the same spirit as the previous one and we disagree again. We do not see why global community would be interested to compare between seagrass

Powered by Editorial Manager® and ProduXion Manager® from Aries Systems Corporation

	and coral reefs, and not equally interested to compare spatial units between Gulf of Mexico and Caribbean Sea. Also, the role of local processes is very important to interpret diversity variation, based on the suggested point of view, no study using analysis between sites should be published. We think that the articulation in a nested design of spatial units (gulfs, sites) and habitats is a methodological strength of our study.<br><br>Figures and Tables<br><br>-The authors should integrate the Figures and Tables in the manuscript as closely as possible to the reference in the text to facilitate the reading of the manuscript.<br>R/ We followed the journal's guideline, that is only figure captions are within the text, and figures at the end.<br><br>-The authors should keep only relevant figures and tables, taking in mind that this is a scientific paper rather than a chapter where considering and including all the possible information is necessary and mandatory.<br>R/ Agree. After the reorganization of the paper, the number of figures was reduced from 11 to 10, and the tables from 3 to 1. Two figures were simplified, and part of the information was sent to supplementary material.
**Additional Information:**	
**Question**	**Response**
**Financial Disclosure**<br><br>Enter a financial disclosure statement that describes the sources of funding for the work included in this submission. Review the submission guidelines for detailed requirements. View published research articles from *PLOS ONE* for specific examples.<br><br>This statement is required for submission and **will appear in the published article** if the submission is accepted. Please make sure it is accurate.	RPE received support from a PhD scholarship (CVU_1080567) granted by the Consejo Nacional de Humanidades, Ciencias y Tecnologías (CONAHCYT). MA received support from a postdoctoral fellowship (CVU_982475) also granted by CONAHCYT. Additional funds were obtained by The Ocean Foundation through the "Proyecto Tres Golfos". The funders had no role in study design, data collection and analysis, decision to publish, or preparation of the manuscript.

**Unfunded studies**<br>Enter: *The author(s) received no specific funding for this work.*<br><br>**Funded studies**<br>Enter a statement with the following details:<br>• Initials of the authors who received each award<br>• Grant numbers awarded to each author<br>• The full name of each funder<br>• URL of each funder website<br>• Did the sponsors or funders play any role in the study design, data collection and analysis, decision to publish, or preparation of the manuscript?<br>• **NO** - Include this sentence at the end of your statement: *The funders had no role in study design, data collection and analysis, decision to publish, or preparation of the manuscript.*<br>• **YES** - Specify the role(s) played.<br><br>* typeset	
**Competing Interests**<br><br>Use the instructions below to enter a competing interest statement for this submission. On behalf of all authors, disclose any competing interests that could be perceived to bias this work—acknowledging all financial support and any other relevant financial or non-financial competing interests.<br><br>This statement is required for submission and **will appear in the published article** if the submission is accepted. Please make sure it is accurate and that any funding sources listed in your Funding Information later in the submission form are also declared in your Financial Disclosure statement.<br><br>View published research articles from *PLOS ONE* for specific examples.	The authors have declared that no competing interests exist.

Powered by Editorial Manager® and ProduXion Manager® from Aries Systems Corporation

**NO authors have competing interests**<br><br>Enter: *The authors have declared that no competing interests exist.*<br><br>**Authors with competing interests**<br><br>Enter competing interest details beginning with this statement:<br><br>*I have read the journal's policy and the authors of this manuscript have the following competing interests: [insert competing interests here]*<br><br>* typeset	
**Ethics Statement**<br><br>Enter an ethics statement for this submission. This statement is required if the study involved:<br><br>• Human participants<br>• Human specimens or tissue<br>• Vertebrate animals or cephalopods<br>• Vertebrate embryos or tissues<br>• Field research<br><br>Write "N/A" if the submission does not require an ethics statement.<br><br>General guidance is provided below. Consult the submission guidelines for detailed instructions. **Make sure that all information entered here is included in the Methods section of the manuscript.**	The collection of sediment samples in Cuban waters was made under the permit LH112-AN-(23)-2014 granted by Centro de Inspección y Control Ambiental (CICA).

**Format for specific study types**<br><br>**Human Subject Research (involving human participants and/or tissue)**<br>• Give the name of the institutional review board or ethics committee that approved the study<br>• Include the approval number and/or a statement indicating approval of this research<br>• Indicate the form of consent obtained (written/oral) or the reason that consent was not obtained (e.g. the data were analyzed anonymously)<br><br>**Animal Research (involving vertebrate animals, embryos or tissues)**<br>• Provide the name of the Institutional Animal Care and Use Committee (IACUC) or other relevant ethics board that reviewed the study protocol, and indicate whether they approved this research or granted a formal waiver of ethical approval<br>• Include an approval number if one was obtained<br>• If the study involved *non-human primates*, add *additional details* about animal welfare and steps taken to ameliorate suffering<br>• If anesthesia, euthanasia, or any kind of animal sacrifice is part of the study, include briefly which substances and/or methods were applied<br><br>**Field Research**<br><br>Include the following details if this study involves the collection of plant, animal, or other materials from a natural setting:<br>• Field permit number<br>• Name of the institution or relevant body that granted permission	
**Data Availability**<br><br>Authors are required to make all data underlying the findings described fully available, without restriction, and from the time of publication. PLOS allows rare exceptions to address legal and ethical concerns. See the PLOS Data Policy and FAQ for detailed information.	Yes - all data are fully available without restriction

A Data Availability Statement describing where the data can be found is required at submission. Your answers to this question constitute the Data Availability Statement and **will be published in the article**, if accepted.<br><br>**Important:** Stating 'data available on request from the author' is not sufficient. If your data are only available upon request, select 'No' for the first question and explain your exceptional situation in the text box.<br><br>Do the authors confirm that all data underlying the findings described in their manuscript are fully available without restriction?	
**Describe where the data may be found in full sentences. If you are copying our sample text, replace any instances of XXX with the appropriate details.**<br><br>• If the data are **held or will be held in a public repository**, include URLs, accession numbers or DOIs. If this information will only be available after acceptance, indicate this by ticking the box below. For example: *All XXX files are available from the XXX database (accession number(s) XXX, XXX.*).<br>• If the data are all contained **within the manuscript and/or Supporting Information files**, enter the following: *All relevant data are within the manuscript and its Supporting Information files.*<br>• If neither of these applies but you are able to provide **details of access elsewhere**, with or without limitations, please do so. For example:<br><br>*Data cannot be shared publicly because of [XXX]. Data are available from the XXX Institutional Data Access / Ethics Committee (contact via XXX) for researchers who meet the criteria for access to confidential data.*<br><br>*The data underlying the results presented in the study are available from (include the name of the third party*	All relevant data are within the manuscript and its Supporting Information files.

Powered by Editorial Manager® and ProduXion Manager® from Aries Systems Corporation

and contact information or URL).<br>• This text is appropriate if the data are owned by a third party and authors do not have permission to share the data.<br><br>* typeset	
Additional data availability information:	Tick here if the URLs/accession numbers/DOIs will be available only after acceptance of the manuscript for publication so that we can ensure their inclusion before publication.

Powered by Editorial Manager® and ProduXion Manager® from Aries Systems Corporation

Manuscript Click here to access/download;Manuscript;Manuscript.docx

Taxonomic, phylogenetic, and functional diversity of mollusk death assemblages in coral reef and seagrass sediments from two shallow gulfs in western Cuban archipelago

Short title: Diversity patterns of mollusk death assemblages

Rosely Peraza-Escarrá[1,2*], Maickel Armenteros[2], Raúl Fernández-Garcés[3], Adolfo Gracia[2]

[1] Posgrado de Ciencias del Mar y Limnología, Universidad Nacional Autónoma de México, Ciudad de México, México.

[2] Instituto de Ciencias del Mar y Limnología, Universidad Nacional Autónoma de México, Ciudad de México, México.

[3] Centro de Estudios Ambientales de Cienfuegos, Ciudad de Cienfuegos, Cuba.

* Corresponding author

Email: roselyperaza2014@gmail.com

Abstract

Mollusks are recognized for their huge diversity and their shell remnants (death assemblages) provide valuable ecological insights. Diversity patterns in tropical marine habitats still are understudied; therefore, we aimed to investigate the taxonomic, phylogenetic, and functional diversity of mollusk death assemblages at regional and local scales in coral reef sands and seagrass meadows. We collected sediment samples at 11 sites in reef sands and seagrass within two shallow gulfs (Batabanó in the Caribbean Sea and Guanahacabibes in the southeastern Gulf of Mexico). All the shells were counted and identified to species level and classified into biological traits. We identified 7113 individuals belonging to 393 species. Diversity and assemblage structure between gulfs showed many similarities given their geological and geographical commonalities. Reef sands had higher richness than seagrasses likely because of a more favorable productivity-disturbance balance. Reef sands were dominated by epifaunal herbivores feeding on microphytobenthos and bisally attached bivalves adapted to intense hydrodynamic regime. In seagrass meadows, suspensivores dominated in exposed sites and chemosynthetic infaunal bivalves dominated in sites where oxygen replenishment was limited. Time averaging of death assemblages was likely in the order of 100 years in our samples, with stronger effects in reef sands compared to seagrass meadows. Our research provides evidence of the outstanding taxonomic and functional diversity of mollusk death assemblages in tropical coastal sediments as resulted of the influence of scale-related processes and habitat type.

Keywords: diversity, mollusk death assemblage, thanatocoenosis, Gulf of Mexico, Caribbean Sea.

Introduction

Death assemblages are defined as the taxonomically identifiable skeletal remains from recent and past generations that are present in the surficial mixed layer on landscapes and seafloors [1, 2]. Studies about ecology of death assemblages provide historical baselines (e.g., [3]) but also insights about modern spatial patterns (e.g., [4]). Some taphonomic processes influence the diversity and ecological structure of death assemblages, being the most important the time averaging and the postmortem lateral transport of shells. Time averaging is the co-occurrence of skeletal remains of individuals that died in different times reducing the temporal resolution of the study [2]. However, a positive point is that time averaging allows death assemblages to reach higher species richness than any single census of their corresponding living assemblage and displays a better mapping of communities and habitat types [5]. On the other hand, the literature suggests that the lateral transport of skeletal remains in bottom sublittoral environments is rather small because [5]: lateral movement of shells is mainly within-habitat, most allochthonous shells are from immediately adjacent habitats, and drift transport affects particularly small thin shells. We focus here on Mollusca, which together with Foraminifera and Echinodermata are the most studied death assemblages in the marine realm.

Mollusca is the 2nd most diverse phylum of metazoans [6] with about 133000 described species [7]. Most mollusks bear a shell of calcium carbonate that lasts in the sediments longer after the dead of the individual providing a rich source of information about diversity. Especially in marine ecosystems, mollusk death assemblages are a promising source of historical data since the analyses of shells provides knowledge about structure (e.g., evenness, composition) and functional features (e.g., productivity, bioturbation potential) [1, 8-11]. Therefore, mollusk death assemblages constitute a powerful and rich-information tool to analyze diversity and to stablishes biological

baselines; particularly in those ecosystems where sampling and knowledge is scarce or null, like the western tropical Atlantic.

To study mollusk assemblages some methodological issues should be addressed given that diversity is a complex concept that includes scales in space and time, and entities such as species, traits, and evolutionary units [12]. Within the last ten years has occurred an accelerate development of approaches and metrics for measuring diversity [13, 14]. Magurran [14] highlights the power of Robert Whittaker's approach that uses complementary facets of diversity (i.e., richness and compositional change) and levels (α-, β-, and γ-diversities). This approach extends to the analysis of species (or taxonomic) diversity, but also includes phylogenetic and functional diversities [14]. [15] and [16] revised the plethora of phylogenetic and functional metrics, respectively, and proposed guidelines for the selection of appropriate metrics. For the analysis of compositional change (β-diversity *sensu lato*), in an outstanding synthesis, [17] proposed the use of turnover and variation for directional and non-directional compositional changes, respectively; they also highlight the power of multivariate measures for β-diversity assessment. By other side, the partition of β-diversity patterns has been a controversial issue based on ecological and mathematical grounds [18]: An approach splits β-diversity in replacement and richness differences [19-21]; while a second approach use replacement and nestedness [22-24]. Evenness also constitutes a key metric of the community when changes in species relative abundance is of interest [13]. Recently, other two unified frameworks for the analyses of diversity that include the relative abundance have been proposed: [12] proposed three types of overall measures (richness, skewness, and divergence) and [25] proposed Hill's numbers for measuring richness and differentiation. Given the bewildering array of approaches for describing diversity, we have chosen some of the above methods that better fit our objectives and type of data.

Habitat type is a tenet of benthic ecology affecting community structure and function [26, 27]. Habitat type is a main driver of the diversity and assemblage structure of alive mollusks at local and regional scales [28]. Therefore, we use the habitat type as an organizing factor in current study of mollusk death assemblages in the western Cuban shelf. Specifically, we focus on sediments from coral reefs and seagrass meadows, two iconic habitats of tropical shelves harboring a huge diversity and complex communities [29, 30]. Coral reefs extent over an important extension of Cuban archipelago and display a large spatial variability regarding cover, average coral size, and health [31]. Given that most coral reef substrate is hard bottom, we selected the sandy spots located in the spur-and-groove biotope within the reef (hereafter "reef sands"). Seagrass meadows also occupy a broad extension of soft bottoms in Cuban shelf and play key roles such as biodiversity hotspot and carbon storage [32]. Therefore, we compare here the sediment assemblages between the two habitat types.

In Cuban archipelago, mollusk diversity and community structure have been the subject of a few studies. Most of the mollusk diversity studies have been done in the Gulf of Batabanó, arguably the best-studied inner sea of the archipelago [28, 33, 34]. Only one study has been done on the deep-sea mollusk assemblages (about 1500 m depth) in the northwestern slope, reporting a very high diversity and potential transport of shells from the shelf [11]. Other three studies using sediment dating with ^{210}Pb and ^{14}C reported historical changes in mollusk death assemblages as response to human disturbances [35-37].

This study is part of a large project aimed to compare the benthic biology between inner seas (gulfs) in the western Cuban shelf, that encompass the southeastern Gulf of Mexico and the western Caribbean Sea. Given the background and knowledge gaps summarized above, the goal of our study is to document the taxonomic, phylogenetic, and functional diversity of mollusk death assemblages in sediment environments within two types of habitats and at two spatial scales. As part of the

diversity assessment, we analyze the two components of diversity (richness and β-diversity) using the species, phylogenetic, and functional dimensions. We also address the evenness and taxonomic/functional composition of death assemblages; in other words, this part considers the relative abundance of the species. Note that a phylogenetic composition is not appropriate due to the nested nature of the Linnean hierarchy.

Materials and methods

Study regions

We sampled two shallow seas (hereafter gulfs) in the western part of Cuban archipelago (Fig 1): Gulf of Batabanó (GB) in the northwestern Caribbean Sea and Gulf of Guanahacabibes (GG) in the southeastern Gulf of Mexico. Both gulfs have dominance of sedimentary bottoms, with shoreline covered by mangrove forests, extensive areas of seagrass meadows (mostly *Thalassia testudinum*) in the central part and bordered by coral reef tracts in the shelf border [35, 38]. Reef sand sites in GB and GG are within the coral reef systems Canarreos and Los Colorados, respectively. Canarreos is a reasonably healthy reef system with the largest individual coral size and reef complexity of the whole Cuban archipelago [31]. Meanwhile, Los Colorados contains a worrying number of depleted reefs with low living coral cover [31]. Seagrass sites differed in the proximity of the shelf border and therefore in the influence of open waters. GB sites, in the shelf border, were subjected to an intense physical disturbance by waving and currents coming from the Caribbean Sea and were relatively far from the pollution sources in the Cuba island. Meanwhile GG sites were farther away from the open ocean, with slower flushing of water by hydrodynamics and more exposed to anthropogenic disturbances such as eutrophication and turbidity [37].

Fig 1. **Map of the study region**. Sampling sites within the gulfs of Batabanó (GB, 4 sites) and Guanahacabibes (GG, 7 sites). Reef sands (orange circles) and seagrass meadows (green triangles). Spatial data from GADM v3.6.

Other similarities between GB and GG are the average depth (about 6–8 m) and the hydrological variables: temperature 27–29 °C, salinity 35.1–37.4 PSU, dissolved oxygen 6.1–8.0 mg L^{-1}, and pH 7.9–8.2. However, there are main differences between GB and GG, such as maximum depth (12 versus 28 m, respectively) and extension (20000 versus 1900 km^2).

Sampling and sample processing

We sampled 11 soft-bottom sites in two expeditions to GB (November 1st–5th, 2015) and GG (May 30th–June 10th, 2014) (Table 1). We selected two types of habitats broadly distributed across the gulfs: coral reefs and seagrass meadows. In coral reefs, we sampled in ~~those~~ sandy spots within spur-and-groove structures in forereefs. Therefore, we are dealing here with mollusk assemblages living in sediments (and not those assemblages on hard substrates).

Table 1. Characterization of the sampling sites in the gulfs of Batabanó and Guanahacabibes. Abbreviations: GB = Gulf of Batabanó, GG = Gulf of Guanahacabibes, S+C = silt plus clay fractions, TOM = total organic matter, Reps. = number of replicates, N = abundance of dead individuals, S = number of species.

Gulf	Habitat	Site	Latitude (N)	Longitude (W)	Depth (m)	S+C (%)	TOM (%)	Reps.	N	S	Observations
GB	Reef sands	GB09	21°34.041	81°38.484	30	18	8	3	1553	181	"Acuario" buoy. Outer reef, clean waters, spur-and-groove biotope, fine and white sands
	Seagrass	GB10	21°36.527	81°34.959	3	26	10	3	744	79	Close to entrance channel of Cayo Largo marina. Meadow (high density) of *T. testudinum* and *S. filiforme*
	Reef sands	GB11	21°35.427	81°55.764	14	12	9	3	1118	166	Outer reef, clean waters, spur-and-groove biotope, fine and white sands
	Seagrass	GB12	21°36.744	81°56.016	4	55	9	3	355	55	Meadow (high density) of *T. testudinum* and algae
GG	Reef sands	GG01	22°14.397	84°43.933	20	-	8	3	364	83	Outer reef, clean waters, spur-and-groove biotope, many gorgonians and sponges, scarce sediment of coarse and white sands
	Reef sands	GG02	22°25.818	84°32.303	18	8	9	1	139	40	Outer reef, clean waters, spur-and-groove biotope, many gorgonians and sponges, scarce sediments, signs of strong hydrodynamics

	Reef sands	GG04	22°18.404	84°40.360	18	13	10	3	660	142	Outer reef, spur-and-groove biotope, many gorgonians and corals, more areas of sandy sediments than in previous sites
	Seagrass	GG05	21°55.556	84°48.483	4	43	13	3	752	83	Meadow composed mostly of *T. testudinum* (medium density) and many algae. Sediment gray and with white sand grains and darker in the top10 cm
	Seagrass	GG06	22°22.792	84°26.019	5	61	10	3	576	58	Meadow composed mostly of *T. testudinum* (medium density), many algae, muddy-sandy bottom rich in detritus, and many epiphytes on blades. Sediment is light gray along the entire core. Some shells >5 cm
	Seagrass	GG09	22°05.030	84°24.206	5	36	8	2	474	71	Meadow of *T. testudinum* (high density), muddy-sandy bottom with many invertebrates
	Seagrass	GG14	22°00.582	84°48.783	5	6	5	2	378	68	Meadow of *T. testudinum* (mid-low density) with short blades and many green algae
Total		11						29	7113	393	

Sediment samples were collected by SCUBA divers using a box core (= sampling unit) with an effective sampling area of 100 cm^2. ~~Usually,~~ three sampling units were collected per site, but in three sites the replication number was lower (Table 1). The box core was pushed 10 cm into the bottom collecting approximately 1000 cm^3 of sediments. A potential caveat with this sampling is that it may miss deep burrowing species inhabiting below 10 cm deep. Sediments were sieved onboard with filtered seawater through a 500-μm sieve and preserved in 70% ethanol. We chose this mesh size to capture the micro-mollusks. In the laboratory, all the mollusk shells were sorted and counted under a stereomicroscope SZX7. In the case of the bivalves, each disarticulated valve was counted as an individual. Mollusk shells were also classified into (i) living individuals by the presence of soft body tissues, which were not included in this study, or (ii) dead individuals with a whole empty shell. Large skeletal fragments retaining the apex or more than half of the hingeline were also counted as dead individuals. Mollusks were identified to species level using taxonomic literature [39-42].

Phylogeny and biological traits

Phylogeny was established by the Linnean system of classification (class, order, family, genus, and species) since no other data were available for most of the species (e.g., DNA-based phylogeny). The taxonomy of the species was obtained by the Match taxa tool of the World Register of Marine Species [7], we used only the accepted names given at WoRMS.

We classified all the species after five biological traits: (i) life mode, (ii) feeding type, (iii) relationship with the substrate, (iv) mobility, and (v) shell attachment (S1 Table). The biological traits were mainly obtained from the database Neogene Marine Biota of Tropical America (NMITA) [43], including support literature for mollusk ecology [40, 41, 44, 45]. All categories

within traits were adapted following the codes given at NMITA. The biological trait information was usually given at genus or family levels, so we extrapolated at the species level if necessary.

Gastropods were classified only after their life mode and the feeding type as no other information was available at NMITA or in the consulted literature. For the Scaphopods, we were able to fill in their traits after [41].

Data analysis

We analyzed the diversity using two complementary components [14]: richness (α- and γ-diversity) and β-diversity. We investigated the three dimensions of diversity: species, phylogenetic, and functional diversity. We also analyzed the ecological structure of the assemblage considering the relative abundance of the species using dominance curves, heat map and numerical ordination according to the appropriate scale. The statistical design, according to the objectives, included two spatial scales: regional that encompassed the gulf and local that referred to the sampling site. We also included habitat in the design because it has a significant impact on the diversity in the studied region [28]. In general, the criterion for testing differences between groups was the overlapping of the 0.95 CIs calculated on basis of permutations.

Richness

For the species richness, we built accumulation curves versus individuals using the effective number of species (Hill's number 0) [46]. The accumulation curves included the observed richness, the asymptotic estimates (only at regional scale), and 0.95 confidence intervals (CIs) generated by

500 permutations using the functions *inext* and *gginext* in the R package iNEXT 3.0.0 [47]. In the second step, we calculated the expected richness at the same level of abundance using rarefaction.

For the phylogenetic richness, we used the Faith's phylogenetic diversity (PD sensu [48]), which is the sum of the branch lengths in a phylogenetic tree. PD measures the amount of evolutionary history across species [15]. The phylogenetic tree was based on the Linnean hierarchy (class, order, family, genus, and species) and built with the function *linnean* in the package BAT 2.9.2 [49].

For the functional richness, we used the Petchey and Gaston's functional diversity (FD sensu [50]), which is the sum of the branch lengths in a functional tree. FD measures the overall distance between the different functional units (i.e., species) of a given assemblage [51]. Related with functional diversity, we used "response traits" that focus on an organism's response to its environment and does not imply that a trait necessarily influences an ecological process [52]. The functional tree was built in the package BAT with the function *tree.build* using the following settings: the traits were converted from categorical to numerical (i.e., dummy variables), Gower distance as resemblance measure, and modified Neighbor-Joining algorithm as linkage strategy. Species, phylogenetic, and functional richness were calculated using the function *alpha* in the R package BAT with rarefied samples (i.e., the same sample size) and using 1000 permutations for generating 0.95 CIs.

β-diversity

We followed the framework proposed by Cardoso et al. [21, 53] that analyzes species-, phylogeny-, and functional-based β-diversity. These three dimensions of total β-diversity were in turn decomposed into two partitions [19, 20]: replacement and richness difference. For the species-based dimension, we built β-diversity accumulation curves for evaluating the effects of sampling

completeness [54]. The pairwise values of dissimilarity were averaged over the relevant scale/level. That is, at regional scale all pairwise inter-site dissimilarities within a gulf were averaged (e.g., for GB: GB09 versus GB10, GB09 versus GB11, etc.). For habitats, pairwise inter-site dissimilarities in a habitat nested within gulf were used (e.g., for reef sands in GB: GB09 vs. GB11; for seagrass in GB: GB10 vs. GB12). β-diversity accumulation curves were built with the function *beta.accum* in the R package BAT. We calculated β-diversity using the function *beta* in the R package BAT. The phylogenetic and functional trees were the same as for richness analyses. The measure of dissimilarity was the Sorensen index that uses incidence data since changes in composition between samples was the target of the analysis. Calculations were made on rarefied samples (at level of smallest sample size) to reduce the effects of differences in abundance and 100 permutations were done to generate 0.95 CIs around the mean β-diversity.

Evenness

This property of the assemblage structure was represented in curves of dominance of species rank (x-axis in log scale) versus relative abundance [55]. We also quantified the rareness based on the number of species with one individual (i.e., singletons) and two individuals (i.e., doubletons).

Taxonomic composition

In a second approach to describe the assemblage structure, we used the SIMPER analysis to identify those species that most contribute to the 70% of similarity (Bray-Curtis index) for a particular scale/level (i.e., gulf, habitat, or site); such species can be considered as the typical or characteristic of that scale/level [55]. For this analysis, we averaged the abundance of species over

the sites. To visualize the typical species indicated by the SIMPER, we built heat maps of their relative abundance. The typical species in the heat map were ordered on basis of their similarity across the samples using an index of association [55]. In the case of analyses at site scale, instead of a heat map, we preferred to build a numerical ordination by non-metric multidimensional scaling (NMDS) with all the replicates to visualize the similarity pattern across sites. NMDS was built on basis of a similarity matrix calculated with the Bray-Curtis index, no transformation of data was done. A permutational analysis of variance (PERMANOVA) [56] was made for testing differences in the multivariate assemblage structure following the nested statistical design mentioned above: GULF as main fixed factor, HABITAT as random factor nested within GULF, and SITE as random factor nested within HABITAT. The settings of PERMANOVA were Bray-Curtis as measure of resemblance, 9999 permutations for building the null distribution, sum of squares type III (partial), and permutation of residuals under a reduced model.

Functional composition

We built four matrices of trait × samples by summing species based on their biological traits: feeding type, relationship organism-substrate, mobility, and shell attachment. The trait life mode (i.e., benthic or pelagic) was not included because there were only six pelagic species. The samples were aggregated according to the scale/level of the analyses (i.e., gulf, habitat, or site). We represented the relative contribution of each trait category to the total abundance in stacked bar plots. Categories within a trait were not independent (i.e., they summed 100%), therefore no statistical tests were done to compare them. For the functional composition across sites, we ordered them according to their similarity based on species abundance using the Bray-Curtis index.

All graphs were made in the package Tidyverse [57] in R.

Results

General features

We identified 7113 dead individuals belonging to 393 species, 242 genera, 101 families, 20 orders, and three classes. Pelagic mollusks occurred only in GB and were represented by six species and 41 individuals (0.6% of total abundance). The full matrix of mollusk species × sites was given in the S2 table. The most abundant species were *Parvilucina* spp. (8%), *Eulithidium* sp. (6%), *Cerithium litteratum* (4%), *Barbatia domingensis* (4%), *Eulithidium bellum* (3%), *Cerithium eburneum* (3%) and *Alvania auberiana* (3%).

Regional scale (Gulfs)

Richness

We identified 280 and 265 mollusk species in the gulfs of Batabanó and Guanahacabibes, respectively. The two accumulation curves of species did not reach asymptotes (S3 Fig). However, the sampling coverage was 0.98 for both gulfs, suggesting a high completeness of the sampling effort. At a level of rarefaction of 3000 individuals, no significant differences of species richness occurred between gulfs (Fig 2a). Nevertheless, the phylogenetic and functional richness were different between gulfs as indicated by the non-overlapped CIs (Figs 2c and 2e). In general, the Gulf of Batabanó tended to have larger richness than Guanahacabibes.

Fig 2. **Regional richness and mean β-diversity of mollusk death assemblages in the gulfs of Batabanó and Guanahacabibes**. (a) Species richness. (b) Species β-diversity. (c) Phylogenetic

richness. (d) Phylogenetic β-diversity. (e) Functional richness. (f) Functional β-diversity. Samples were rarefied to 3000 individuals in (a), (c), and (e); while in (b), (d), and (f) were rarefied to the lowest number of individuals between pairs of sites. Two additive partitions of β-diversity are shown: replacement and richness difference. Horizontal lines = mean, boxes (or whiskers) = 0.95 confidence intervals, and vertical lines = range. Phylo. = phylogenetic, Funct. = functional

β-diversity

The accumulation curves suggested a modest decrease of mean β-diversity with the increase of the replication suggesting that the current sample size (n = 3, for most of the sites) is enough for a robust comparison (S3 Fig). The total species β-diversity was high (> 0.6) and similar for GB and GG (Fig 2b). The replacement partition was higher than richness difference partition in both gulfs. Phylogenetic β-diversity was similar between the gulfs (around 0.5), and with replacement partition being higher than richness difference as well (Fig 2d). Functional β-diversity had the same pattern, although total values of β-diversity were lower (< 0.5) (Fig 2f).

Evenness

The evenness was similar in the two gulfs as indicated by the similar shape of the dominance curves (Fig 3a). The main difference between the two curves is the level of dominance of the most abundant species, which was 17% and 7% in GG and GB, respectively. About nine species contributed less than 5% each to the total abundance in any gulf followed by a long tail of rare species represented by singletons (71 in GB and 77 in GG) and doubletons (38 in GB and 48 in GG).

Fig 3. Evenness of mollusk death assemblages. Dominance curves of relative contribution of species to the total abundance versus abundance species rank. (a) Regional scale. (b) Habitat. (c) Site scale

Taxonomic composition

The heat map representing the assemblage structure of 25 typical species for the gulfs suggested that they were largely the same, despite variability in the relative abundances (S4 Fig). In other words, of these 25 typical species, only two species (*Smaragdia viridis* and *Ervilia nitens*) occurred in a single gulf (GB), the other 23 species occurred with variable importance in both gulfs.

Functional composition

The trait feeding type was represented by five dominant categories, the category “others” lumped together to: deposit feeders, herbivores/suspensivores, and herbivores/carnivores. Herbivores dominated the functional trophic structure (Fig 4a). The most striking difference between gulfs was the larger abundance of chemosynthetic deposit feeders in GG. The trait relationship organism-substrate included three dominant categories, and the category “others” lumped together to: nestler, borer, and the mixed categories epifaunal/semi-infaunal/borer. The dominant category was infauna, followed by epifauna, and facultative epifauna/infauna (Fig 4b). The two gulfs had a similar contribution of each category to the total abundance. The trait mobility was represented by two dominant categories, and the category “others” lumped together to: swimming, immobile, and the mixed categories actively mobile/sedentary/immobile. The actively mobile forms strongly

dominated in the two gulfs in terms of abundance, followed by sedentary forms (Fig 4c). The trait shell attachment included two dominant categories and the category "others" lumped together to: cemented and mixed categories unattached/bysally attached. The unattached forms were dominant in both gulfs, however the bysally attached forms were more important in GB when compared with GG (Fig 4d).

Fig 4. Regional functional composition of mollusk death assemblages in the gulfs of Batabanó (GB) and Guanahacabibes (GG). (a) Feeding types, including carnivore, chemosymbiotic deposit feeder, herbivore/deposit feeder (facultative), herbivore, suspension feeder, and others. (b) Relationships organism – substrate, including infauna, epifauna, epifauna/infauna (facultative), and others. (c) Mobility, including actively mobile, sedentary, and others. (d) Shell attachment, including unattached, bysally attached, and others. The traits in (b), (c), and (d) were calculated only for bivalves. See the text for details about "others"

Habitat

Richness

We used the nested structure to compare two habitat types within the corresponding gulfs. The species accumulation curves were not asymptotic (S3 Fig). However, the sampling coverage was high as well, ranging between 0.95 and 0.98. Reef sands had higher mollusk species richness than seagrass; and within seagrass meadows, gulf of Guanahacabibes had higher richness than Batabanó (Fig 5a). Phylogenetic richness had the same pattern, with higher richness in the reef sands, intermediate in seagrass from Guanahacabibes, and lowest in seagrass from Batabanó (Fig 5c).

Functional diversity showed a different pattern, with higher richness in reef sands from Batabanó, and lower in the other three groups (Fig 5e).

Fig 5. Habitat richness and mean β-diversity of mollusk death assemblages in reef sands (RS) and seagrass (S) within the gulfs of Batabanó (GB) and Guanahacabibes (GG). (a) Species richness. (b) Species β-diversity. (c) Phylogenetic richness. (d) Phylogenetic β-diversity. (e) Functional richness. (f) Functional β-diversity. Samples were rarefied to 1000 individuals in (a), (c), and (e); while in (b), (d), and (f) were rarefied to the lowest number of individuals between pairs of sites. Two additive partitions of β-diversity are shown: replacement and richness difference. Horizontal lines = mean, boxes (or whiskers) = 0.95 confidence intervals, and vertical lines = range. Phylo. = phylogenetic, Funct. = functional

β-diversity

β-diversity accumulation curves showed a moderate decrease from 1 to 3 sampling units (S3 Fig) indicating that current sample size was enough for a robust comparison. There were no differences of species β-diversity between reef sands and seagrass in the Gulf of Batabanó. However, in the Gulf of Guanahacabibes the species β-diversity was slightly higher in seagrass compared with reef sands (Fig 5b). The replacement partition was much more important than richness differences for all the combinations habitat-gulf. The phylogenetic β-diversity was similar between the two habitats nested within gulfs, and the replacement partition was also the larger (Fig 5d). The functional β-diversity did not show differences between habitats nested within gulfs (Fig 5f). But the richness differentiation partition increased relatively (around 50% of total β-diversity) suggesting that

loss/gain of trait categories played a more important role than simple replacement of one category by another.

Evenness

There were differences among the habitats nested within gulfs as indicated by the four curves (Fig 3b). However, seagrass had larger dominance compared to reef sands, given by the two most abundant species (23% and 15% in GG and GB, respectively). The 2nd and 3rd most abundant species for both types of habitats contributed between 5% and 12% of the total, suggesting a rather high evenness. As expected, there was a long tail of rare species represented by an average (over the four combinations) of 52 singletons and 31 doubletons.

Taxonomic composition

The heat map of typical species suggested sharp differences between habitats. In GB, there were 14 species typical of reef sands but absent in seagrass, and conversely three species typical of seagrass but absent in reef sands (Fig 6). By other side, in GG, there were eight species typical of reef sands but absent in seagrass, and four species typical of seagrass but absent in reef sands.

Fig 6. Assemblage structure of mollusk death assemblages in reef sands (RS) and seagrass (S) within the gulfs of Batabanó (GB) and Guanahacabibes (GG). Heat map of relative abundance (in %) of those typical species that contribute to the 70% of similarity within each habitat. Note that species were ordered by their similarity across the habitat-gulf combinations

Functional composition

Feeding type structure had notable differences between habitats (Fig 7a). The feeding type structure in reef sands from the two gulfs was similar, with dominance of herbivores, followed by carnivores and suspension feeders. Feeding type structure in seagrass was different compared to reef sands, but also had differences between GB and GG. For instance, in GB suspension feeders was the most important category, while in GG was chemosynthetic deposit feeders. The relationship organism-substrate was quite different between habitats nested within gulfs (Fig 7b). In reef sands, epifauna represented more than half of the abundance, while in seagrass they were rather scarce. Seagrass, in both gulfs, were largely dominated by infauna. Mobility had a similar pattern with sharp differences between habitats (Fig 7c). In reef sands, sedentary forms were similarly abundant to actively mobile forms. However, in seagrass the sedentary forms virtually disappeared, and only actively mobile forms occurred. Shell attachment had a similar pattern to the other two previous traits. There were notably differences between habitats nested within gulfs (Fig 7d). In reef sands, bysally attached and unattached forms were roughly equally important, and the category other occurred in much lesser proportion. While in seagrass, unattached forms largely dominated the assemblages.

Fig 7. Habitat functional composition of mollusk death assemblages in reef sands (RS) and seagrass (S) within the gulfs of Batabanó (GB) and Guanahacabibes (GG). (a) Feeding types including carnivore, chemosymbiotic deposit feeder, herbivore/deposit feeder (facultative), herbivore, suspension feeder, and others. (b) Relationships organism - substrate including infauna, epifauna, epifauna/infauna (facultative), and others. (c) Mobility, including actively mobile, sedentary, and others. (d) Shell attachment, including unattached, bysally attached, and others. The traits in (b), (c), and (d) are only for bivalves. See the text for details about “others”

Local scale (sites)

Richness

The shapes of the species accumulation curves for sites were non-asymptotic (S3 Fig). However, the sampling coverage was relatively high and ranged from 0.86 at GG02 (with single replicate) to 0.97 at GB09 and GB10. The most striking point in the curves was the large abundance of dead shells in the reef sand sites GB09 and GB11. The species richness varied significantly across sites with the most obvious differences between reef sand sites (GB09, GB11, GG01, GG02, and GG04) with higher richness and seagrass sites with lower richness (Fig 8a). The phylogenetic richness showed a similar pattern with high variability across sites, and significant differences between reef sand and seagrass sites (Fig 8c). Functional richness did not show differences across sites due to the overlapping of the 0.95 CIs (Fig 8e).

Fig 8. Local richness and mean β-diversity of mollusk death assemblages in 11 sites within the gulfs of Batabanó (GB) and Guanahacabibes (GG). (a) Species richness. (b) Species β-diversity. (c) Phylogenetic richness. (d) Phylogenetic β-diversity. (e) Functional richness. (f) Functional β-diversity. Samples were rarefied to 300 individuals in (a), (c), and (e); while in (b), (d), and (f) were rarefied to the lowest number of individuals between pairs of sites. Two additive partitions of β-diversity are shown: replacement and richness difference. Horizontal lines = mean, boxes (or whiskers) = 0.95 confidence intervals, and vertical lines = range. Phylo. = phylogenetic, Funct. = functional

β-diversity

Species β-diversity was around 0.5, with no differences among sites as suggested by the overlapping of the 0.95 CIs. The most important contribution to the total β-diversity was given by species replacement (Fig 8b). Phylogenetic β-diversity was in general lower than 0.5, but again without clear differences among sites. Most of the phylogenetic β-diversity was given by species replacement (Fig 8d). Functional β-diversity had a rather similar pattern, without differences among sites, but a larger relative contribution of richness differences to the total β-diversity (Fig 8f).

Evenness

The evenness was similar for the 11 sites based on the dominance curves (Fig 3c). However, two seagrass sites in Guanahacabibes had higher dominance of a single species (GG06 with 41% and GG09 with 37%). A long tail of rare species was represented by a mean number of singletons over the 11 sites of 37 species (range: 19–68 species) and a mean number of doubletons of 18 species (range: 4–38 species).

Taxonomic composition

For analyzing changes in taxonomic composition at site scale, we searched for the similarity patterns across sites. A NMDS ordination plot of the 29 samples (= points) based on species abundance indicated a clear pattern based on the habitat type (Fig 9). Reef sands and seagrass sites clustered apart from each other in the plot and showed significant differences (PERMANOVA, S5 table). However, differences in assemblage structure between gulfs were less marked in the plot and non-significant after PERMANOVA. The variability between replicates from the same site was also

considerable and statistically significant (PERMANOVA, S5 table) suggesting high variability in the assemblage structure. Indeed, factors HABITAT and SITE explained about one third each of the total variance, meanwhile remaining third was unexplained (= residual).

Fig 9. Assemblage structure of mollusk death assemblages within the gulfs of Batabanó (GB) and Guanahacabibes (GG). Similarity patterns in a NMDS ordination plot of 29 samples from 11 sites based on species abundance. Samples were coded by habitat (color and shape) and gulf (open/close symbol)

Functional composition

We ordered the sites along the x-axis based on their similarity using the species abundance. The feeding type structure showed differences related with the habitats again (Fig 10a). Sites within the reef sands (GG09, GB11, GG01, GG04, and GG02) had herbivores as the most important category, followed by carnivores. Heterogeneity in feeding type composition was larger in seagrass; some sites (GB12, GG05, GG09, and GG06) had an important contribution of chemosynthetic deposit feeders, while at GG14 dominated herbivores and at GB10 dominated suspension feeders. The relationship organism-substrate had large heterogeneity in reef sand sites (Fig 10b). Three reef sand sites (GB11, GG01, and GG04) had dominance of epifaunal forms, while at GG09 dominated infauna, and in GG02 facultative epifauna/infauna. In seagrass sites, infaunal forms were dominant, although epifauna was important at GG09. The mobility had a rather regular pattern also associated to the habitat (Fig 10c). Three reef sand sites (GB11, GG01, and GG04) had dominance of sedentary forms, while seagrass sites (GB10, GB12, GG14, GG05, GG09, and GG06) and two reef sand sites (GG02 and GB09) were largely dominated by actively mobile forms. The shell

attachment had a similar pattern of habitat influence (Fig 10d). Three reef sand sites (GB11, GG01, and GG04) had dominance of bysally attached forms. The other two reef sand sites (GG02 and GB09) had important contributions of bysally attached forms, but the dominance was given by unattached forms. The latter category was largely the most important in seagrass sites.

Fig 10. Local functional composition of mollusk death assemblages in 11 sites within the gulfs of Batabanó (GB) and Guanahacabibes (GG). (a) Feeding types including carnivore, chemosymbiotic deposit feeder, herbivore/deposit feeder (facultative), herbivore, suspension feeder, and others. (b) Relationships organism - substrate including infauna, epifauna, epifauna/infauna (facultative), and others. (c) Mobility includes actively mobile, sedentary, and others. (d) Shell attachment including unattached, bysally attached, and others. The traits (b), (c), and (d) are only for bivalves. See the text for details about “others”. Note that the sites were ordered by their similarity based on species abundance

Discussion

Our data provide a synoptic picture of the mollusk death assemblages in two habitats within two shallow gulfs: Batabanó (GB) in the Caribbean Sea and Guanahacabibes (GG) in the Gulf of Mexico. We detected ecological patterns related to the scale and habitat type. Our data are robust despite of potential taphonomic biases (see below) of death assemblages. We identified 7113 shells belonging to 393 species, that can be considered as a large sample size. However, the curves of species accumulation, particularly for habitats and at site scale, were hardly asymptotic suggesting that hyper-diverse assemblages may never reach an asymptote [58]. Estimates of sampling coverage

were in general acceptable (i.e., > 90%) suggesting a fair description of the diversity and assemblage structure of mollusk death assemblages.

Mollusk death assemblages at regional scale

The two gulfs showed coherent similarity in terms of species richness, β-diversity, evenness, taxonomic and functional assemblage structures (except for the feeding type); also, PERMANOVA indicated no statistical differences. Similar geological and geographical settings in the western region of Cuba Island may account for these commonalities between Batabanó and Guanahacabibes. For instance, β-diversity, assemblage composition and functional structure may be explained by three general factors after [59]. First, abiotic heterogeneity, which was similar between the two gulfs given the occurrence of typical tropical habitats in both basins (e.g., mangroves, muddy bottoms, seagrass meadows, and coral reefs). The abiotic data of water column and sediments, although with similar average values between gulfs, varied notably within gulfs evidencing the environmental heterogeneity. Second, the regional pool of species should be similar in both gulfs given they belong to the same biogeographic realm (i.e., tropical western Atlantic, [60]). Third, the dispersal rates, which are related to species' biology and on the oceanographic regime. However, it was not possible to compare both gulfs in terms of the oceanographic regime because of the lack of available data.

There were two main differences between the gulfs: richness (phylogenetic and functional) and feeding type composition. First, phylogenetic and functional richness were higher in Batabanó maybe because it has a more advantageous balance of productivity-disturbance. According to [61], richness depends on the ecological time of the regimes of productivity and disturbance. The eastern portion of the Gulf of Batabanó, where the four sites were located (Fig 1), is an area of high

biological productivity due to the confluence of freshwater (and nutrients) runoff from Zapata Swamp and the influence of the Caribbean current on the shelf [62]. Local upwellings, eddies and tidal currents have been reported to promote the exchange between the open ocean and the southeastern portion of Gulf of Batabanó [63]. Additional evidence of the large productivity of eastern Batabanó is the healthy coral reefs status with coral colonies of large size [31] and the high finfish and spiny lobster catches [64]. Besides, the disturbance regime seems to be high in the shelf border of eastern Batabanó. High contribution of bysally attached bivalves, adapted to strong currents, and the occurrence of skeletal remnants of oceanic pelagic species suggest stronger influence of open ocean. In addition, reef sands in Batabanó exhibited the largest number of shells (and total species richness) suggesting high secondary productivity (S2 Fig).

The feeding type composition was different between Batabanó and Guanahacabibes, with higher importance of chemosynthetic bivalves (Lucinids) in the latter. These bivalves dominated in sulfide-rich hypoxic sediments [65], like the four seagrass sites located in the inner part of Guanahacabibes. On contrary, the seagrass sites in Batabanó did not show symptoms of hypoxia (personal observation) likely because oxygen replenishment due to intense hydrodynamics. The different functional composition was also reflected in the evenness between gulfs: In Guanahacabibes the dominance was higher than in Batabanó due to the occurrence of *Parvilucina* spp. (17%).

Two additional regional patterns were related to β-diversity. The first pattern was derived from the comparison of β-diversity dimensions (i.e., species versus phylogenetic versus functional). The species β-diversity was the highest (about 0.6) because of the high number of rare species that promote the differences between sites. By other side, functional β-diversity was the lowest (about 0.3) likely reflecting that taxonomically different species were replaced by functionally similar trait combinations, which are the ones that evolved adaptations to the benthic environments in reef sands and seagrass. Similar findings were reported by [66] for diatom assemblages. Phylogenetic β-

diversity was intermediate (about 0.5) because composition at higher taxonomic level (i.e., genus, family, etc.) reflected a balance between species rareness and similar functional trait combinations.

A second regional pattern of β-diversity, for the three diversity dimensions, was the larger contribution of the replacement partition compared to the richness partition. [18] stated that replacement is due to ecological tolerance or niche breadth, while richness is due to the number of niches. Our findings broadly agree with latter statements because many species with potentially different niche breadth occurred in both gulfs. While there was a similar number of available niches in the gulfs because they share geological and geographical features; for instance, same habitats (e.g., mangroves, seagrass meadows, and coral reefs) and same engineer species (e.g., *Thalassia testudinum* in seagrass and *Orbicella faveolata* in forereefs).

We recorded high evenness of the assemblages at regional scale, with the presence of one moderately abundant species and a very long tail of rare species. This pattern agrees with the very high richness and low dominance of mollusk coastal assemblages in Cuban archipelago as reported by other authors [33-35, 37]. The high evenness reported in our study is also enhanced by two features [5]: (1) time averaging increases the probability of several shifts in species dominance through time; although we have evidence of limited time averaging in our study (see below). And (2), at regional scale there is more tendency to reflect the share dominance of multiple species within a window of time, resulting in lower dominance.

Mollusk death assemblages: Reef sands versus seagrass meadows

Habitat type had a strong influence on all the metrics of assemblages: Richness, β-diversity, evenness, taxonomic and functional assemblage structures; PERMANOVA also indicated a statistically significant effect of the habitat type. This supports the strong role of habitat type on the diversity and assemblage structure of dead [10, 67, 68] and live mollusks [28].

Coral reefs harbor a high richness of mollusks in their different substrates [69]. The relationship between productivity and disturbance may also explain this pattern [61] since coral reefs receive high quality organic carbon produced by a diverse group of primary producers that includes microphytobenthos, macroalgae, zooxanthella and phytoplankton [29]. At the same time, disturbance is strongly related to physical forces from waves and currents, so species may require adaptation against resuspension (e.g., bisally attached bivalves) but promotes oxygen replenishment avoiding hypoxia and generation of sulfur hydrogen in sediments. As opposite, although seagrass harbor a large biodiversity, the balance of productivity-disturbance is likely less favorable. For instance, the fraction of primary production of seagrass is highly refractory due to the content of lignin, so the most labile fraction (e.g., derived from epiphytic microalgae) is that is mostly used by grazer mollusks, mainly gastropods [70]. Seagrass canopy reduces the physical disturbance and associated resuspension risk of benthic organisms, but at the same time reduces oxygen replenishment causing hypoxia and generation of hydrogen sulfide [65].

Each habitat had a distinctive assemblage structure suggesting that the identity can be preserved in the dead assemblages long after death [4]. It is explained by differences in richness (see above) and adaptative traits (see below), but also for a rather limited shell transport between adjacent habitats. The transport of dead shells is an important postmortem process [5, 71], but likely the physical structure of the habitats may reduce it. Seagrass meadows are physically protected environments because canopy and rhizomes reduce the erosion by currents and waves [70], meanwhile spur-and-grooved structures in coral reefs are also physically sheltered by rocky reef structures [72]. [73]

reported similar findings about reduced lateral shell transport in seagrass meadows and coral reefs in a study on fidelity of live and death assemblages. These authors explained that the reduced out-of-habitat transport is due to soft-sediment patches surrounded by corals in coral reefs and by dense leave/rhizome mats in seagrass meadows.

The functional structure changed between the two habitat types reflecting niche preferences. Reef sands were characterized by mollusk assemblages dominated by epifaunal herbivores adapted to graze on the microphytobenthos; in particular, grazer gastropods contributed importantly to this guild. In sandy spots, microphytobenthos has a large primary productivity and provides high food availability to the benthic consumers [74, 75]. Interestingly, bivalve suspension feeders were not dominant in reef sands, likely reflecting low availability of suspended particles in the sandy spots. Sedentary species, mostly bisally attached bivalves, also thrived in reef sands reflecting the adaptation to the intense hydrodynamic, specifically to the resuspension by currents. In seagrass meadows, chemosynthetic detritivores were more important, suggesting that much of the primary production done in the seagrass canopy reach the bottom as detritus. Seagrass from Batabanó and Guanahacabibes differed in the importance of suspension feeders and chemosynthetic bivalves reflecting the influence of hydrodynamic regime on the functional structure. Suspension feeders dominated Batabanó’s seagrasses because of intense hydrodynamic regime in the border of the shelf. Current velocity exerts a physical disturbance on grazers, although it is counteracted by the shoot density of the seagrass meadow [76]. In Batabanó’s seagrass, the high shoot density reported by us (Table 1) possibly enhanced the protection against resuspension promoting the dominance of unattached forms. Meanwhile chemosynthetic lucinid bivalves were more important in Guanahacabibes because slower hydrodynamics reduce oxygen replenishment and promote hydrogen sulfide generation in sediments [65]. Infaunal, actively mobile and unattached forms

dominated in all seagrass meadows probably as an adaptation to look for more oxygenated spots from reduced sediments and/or foraging for less refractory detritus.

Mollusk death assemblages at local scale

Local species richness reflected the combined influence of the regional pool of species [77] and habitat type [28]. In our study, variability of species richness from site to site, within same gulf and habitat type, responded to the local environment. For instance, the highest richness and abundance in GB11 and GG04 responded to local environment conditions that enhanced deposition of shells in sandy spots within spur-and-groove structures.

The total β-diversity at local scale showed moderate shifts from site to site mostly because of differences in abiotic heterogeneity [59]. In terms of β-diversity partitions, many different species with potentially different niche breadth may explain the higher replacement partition of β-diversity across sites [18]. Conversely, similar number of niches across sites within the same habitat explains the lower contribution of richness partition.

Evenness curves indicated two sites subjected to disturbance where few species of lucinids tolerant to deoxygenation were dominant: GG06 and GG09. Historical trends of richness and abundance in sites GG06 and GG09, based on core stratigraphy and ^{210}Pb dating, indicated significant decline of mollusk diversity as result of sedimentation and eutrophication [37]. The pattern of similarity among replicates, represented in the NMDS, strongly suggests the overarching effect of habitat type on the assemblage structure, which agrees with other studies (e.g., [69]). Nevertheless, substantial variability among replicates within a site occurred probably as response of assemblages to small-scale sediment heterogeneity [78-80]. Indeed, SITE explains about one third of the total variance as indicated by PERMANOVA.

Effects of time averaging on the diversity outcomes

The process of time averaging explains the large richness of the mollusk death assemblages in our study [5]. Instead of a bias, the time averaging can be considered as a scaling post-mortem process that averages the input of shells over time [2]. Thus, we can expect that our death assemblage study may inform more about biodiversity than a single snap-shot study using only the living mollusks. The extension of the time averaging depends on some non-exclusive processes such as [1]: (i) the intrinsic durability of the remains, (ii) the extent of the physical and biological reworking processes that can bury or exhume the remains within the mixed layer of sediments, and (iii) the sedimentation rates.

An accurate estimation of the stratigraphic resolution and time averaging in the Holocene-Anthropocene record ultimately requires that age models are coupled with post-mortem age-frequency distributions of shells dated by amino-acid racemization, ^{14}C or U-Th [3]. Nevertheless, latter authors also stated that simple age models for cores thus still can be informative about the temporal scale of fossil assemblages even in the absence of postmortem age-frequency data [3]. On this basis, we analyzed the ^{210}Pb age models published in [37] from the same seagrass sites in Guanahacabibes looking for evidence about time averaging in our study. The age models based solely on sediments and published in [37] have the advantage that samples were taken at short distances (< 100 m) of sites in current study and using techniques (barrel corer) that effectively samples the top ten centimeters of sediment.

We estimated that the temporal framework spanned by the death assemblages in our study was about 100 years based on two lines of evidence. First, the top ten centimeters of sediments at sites GG05, GG06, and GG09a spanned a period (relative to 2014, when the sampling was done) of 58,

99, and 81 years, respectively [37]. Second, time averaging will tend to be low if stratigraphic resolution (and sedimentation rate) is very high, regardless of mixing depths and the rate of disintegration [3]. In Guanahacabibes, the sedimentation rate was high as assessed from mass accumulation rates and dating. The temporal framework of about 100 years in our study supports the value of death assemblages to analyze the diversity of mollusk assemblages in the ecological time.

Our evidence also suggests that time averaging was lower in seagrass meadows compared to reef sands due to lower reworking and higher sedimentation rate [1]. In reef sands the time averaging probably spanned a longer period because they were like open shelf environments in terms of hydrodynamics with stronger reworking and lower sedimentation rate [5]. Even when the time averaging in the reef sands was probably higher compared to seagrass, we hypothesize that in both habitat types the assemblage composition have been not long-time averaged due to the high species-, phylogenetic- and functional β-diversities [2, 67].

Conclusions

At regional scale, diversity and community structure between gulfs showed many similarities likely because geological and geographical commonalities. Habitat type had a strong effect on all the diversity metrics of assemblages. Reef sands had higher species, phylogenetic, and functional richness than seagrass meadows probably influenced by more favorable productivity-disturbance balance. The taxonomic composition was distinctive for each habitat type suggesting a limited lateral transport of dead shells between adjacent sites. Functional composition based on biological traits reflected niche properties of species adapted to each habitat. Reef sands were characterized by epifaunal herbivores (mostly gastropods) adapted to feed on microphytobenthos growing in sandy

spots and bisally attached bivalves adapted to intense hydrodynamics. In seagrass meadows, hydrodynamic regime influenced the functional structured, with dominance of suspensivores in exposed sites in Batabanó and dominance of chemosynthetic infaunal bivalves in Guanahacabibes where oxygen replenishment is more limited. On a local scale, sites had moderate differences because of small-scale environmental variability. Two seagrass sites closer to the coastline showed evidence of impact due to eutrophication and sedimentation. Time averaging was likely in the order of 100 years in our samples, with stronger effects in reef sands compared to seagrass meadows. Our research provides evidence of the outstanding taxonomic and functional diversity of mollusk death assemblages in tropical coastal sediments as resulted of the influence of scale-related processes and the habitat type.

Acknowledgements

R Peraza-Escarrá thanks to PhD Graduate Program “Ciencias del Mar y Limnología” at Instituto de Ciencias del Mar y Limnología (UNAM) and to Consejo Nacional de Humanidades, Ciencias y Tecnologías (CONAHCYT). We acknowledge the crew of the R/V Felipe Poey. We thank the Centro de Estudios Ambientales de Cienfuegos, particularly Misael Díaz-Asencio and Lisbet Díaz-Asencio, for sediment analyses. We thank Dania Saladrigas and Lorena González for sample processing, Abel Valdivia for providing the map, and Fernando Bretos and Daria Siciliano for their support in the field and technical assistance. The collection of sediment samples in Cuban waters was made under the permit LH112-AN-(23)-2014 granted by Centro de Inspección y Control Ambiental (CICA). We acknowledge very much the comments by Susan Kidwell and one anonymous reviewer that improved the manuscript.

Author contribution

RPE: Data Curation, Formal Analysis, Investigation, Writing - Original Draft, Preparation, and Writing - Review & Editing. MA: Conceptualization, Formal Analysis, Funding Acquisition, Resources, Supervision, Writing - Original Draft Preparation, and Writing - Review & Editing. RFG: Data Curation, and Writing - Review & Editing. AG: Conceptualization, Funding Acquisition, Resources, Supervision, Writing - Original Draft Preparation, and Writing - Review & Editing.

References

[1] Kidwell SM. Time- averaging and fidelity of modern death assemblages: building a taphonomic foundation for conservation palaeobiology. Paleontology. 2013;56: 487–522. doi: 10.1111/pala.12042.

[2] Kidwell SM, Tomašových A. Implications of time-averaged death assemblages for ecology and conservation biology. Annu Rev Ecol Evol Syst. 2013;44: 539–563. doi: 10.1146/annurev-ecolsys-110512-135838.

[3] Tomašových A, Dominici S, Nawrot R, Zuschin M. Temporal scales, sampling designs, and age distributions in marine conservation palaeobiology. Geol Soc Spec Publ. 2023;529: SP529–2023. doi: 10.1144/SP529-2022-361.

[4] Casebolt S, Kowalewski M. Mollusk shell assemblages as archives of spatial structuring of benthic communities around subtropical islands. Estuar Coast Shelf Sci. 2018;215: 132–43. doi: 10.1016/j.ecss.2018.09.023.

[5] Kidwell SM, Bosence D. Taphonomy and time-averaging of marine shelly faunas. In: Allison PA, Briggs D (Eds.), Taphonomy: Releasing the data locked in the fossil record. Plenum Publishers. 1991. pp. 115–209.

[6] Zhang Z. Animal Biodiversity: An update of classification and diversity in 2013. Zootaxa. 2013;3707: 5–11. doi: 10.11646/zootaxa.3703.1.3.

[7] WoRMS. World Register of Marine Species. Available from https://www.marinespecies.org at VLIZ. Accessed 2023-04-17. doi: 10.14284/170.

[8] Grill B, Zuschin M. Modern shallow-to deep-water bivalve death assemblages in the Red Sea—ecology and biogeography. Palaeogeogr, Palaeoclimatol, Palaeoecol. 2001; 168: 75–96. doi: 10.1016/S0031-0182(00)00250-9.

[9] Tomašových A, Kidwell SM. Preservation of spatial and environmental gradients by death assemblages. Paleobiology 2009;35: 119–145. doi: 10.1666/07081.1.

[10] Weber K, Zuschin M. Delta-associated molluscan life and death assemblages in the northern Adriatic Sea: implications for paleoecology, regional diversity and conservation. Palaeogeogr Palaeoclimatol Palaeoecol. 2013;370: 77–91. doi: 10.1016/j.palaeo.2012.11.021.

[11] Peraza-Escarrá R, Armenteros M, Fernández-Garcés R, Murawski SA, Gracia A. Mollusk death assemblages from the deep slope off northwestern Cuba (Gulf of Mexico). Bull Mar Sci. 2022;98: 451–470. Doi: 10.5343/bms.2022.0006.

[12] Pavoine S, Bonsall MB. Measuring biodiversity to explain community assembly: A unified approach. Biol Rev. 2011;86: 792–812. Doi: 10.1111/j.1469-185X.2010.00171.x.

[13] Magurran AE. Measuring biological diversity. Oxford: Blackwell; 2004. 257 p.

[14] Magurran AE. Measuring biological diversity. Curr Biol. 2021;31: R1174–1177. Doi: 10.1016/j.cub.2021.07.049.

[15] Tucker CM, Cadotte MW, Carvalho SB, Davies TJ, Ferrier S, Fritz SA, et al. A guide to phylogenetic metrics for conservation, community ecology and macroecology. Biol Rev. 2017;92: 698–715. Doi: 10.1111/brv.12252.

[16] Mammola S, Carmona CP, Guillerme T, Cardoso P. Concepts and applications in functional diversity. Funct Ecol. 2021;35: 1869–1885. Doi: 10.1111/1365-2435.13882.

[17] Anderson MJ, Crist TO, Chase JM, Vellend M, Inouye BD, Freestone AL, et al. Navigating the multiple meanings of β diversity: a roadmap for the practicing ecologist. Ecol Lett. 2011;14: 19–28. Doi: 10.1111/j.1461-0248.2010.01552.x.

[18] Legendre P. Interpreting the replacement and richness difference components of beta diversity. Glob Ecol Biogeogr. 2014; 23: 1324–1334. Doi: 10.1111/geb.12207.

[19] Carvalho JC, Cardoso P, Gomes P. Determining the relative roles of species replacement and species richness differences in generating beta-diversity patterns. Glob Ecol Biogeogr. 2012;21: b760–771. Doi: 10.1111/j.1466-8238.2011.00694.x.

[20] Carvalho JC, Cardoso P, Borges PA V, Schmera D, Podani J. Measuring fractions of beta diversity and their relationships to nestedness: a theoretical and empirical comparison of novel approaches. Oikos. 2013;122: 825–834. Doi: 10.1111/j.1600-0706.2012.20980.x.

[21] Cardoso P, Rigal F, Carvalho JC, Fortelius M, Borges PA V, Podani J, et al. Partitioning taxon, phylogenetic and functional beta diversity into replacement and richness difference components. J Biogeogr. 2014;41: 749–761.

[22] Baselga A. Partitioning the turnover and nestedness components of beta diversity. Glob Ecol Biogeogr. 2010;19: 134–43. doi: 10.1111/j.1466-8238.2009.00490.x.

[23] Baselga A. The relationship between species replacement, dissimilarity derived from nestedness, and nestedness. Glob Ecol Biogeogr. 2012;21: 1223–32. doi: 10.1111/j.1466-8238.2011.00756.x.

[24] Baselga A, Leprieur F. Comparing methods to separate components of beta diversity. Methods Ecol Evol. 2015;6: 1069–79. doi: 10.1111/2041-210X.12388.

[25] Chao A, Chiu CH, Jost L. Unifying species diversity, phylogenetic diversity, functional diversity, and related similarity and differentiation measures through Hill numbers. Annu Rev Ecol Evol Syst. 2014;45:297–324. doi: 10.1146/annurev-ecolsys-120213-091540.

[26] Southwood TRE. Habitat, the templet for ecological studies? J Anim Ecol. 1977;46: 337–365.

[27] Ferraro SP. Ecological periodic tables: In principle and practice. Oikos 2013;122: 1541–53.

[28] Armenteros M, Saladrigas D, González-Casuso L, Estevez ED, Kowalewski M. The role of habitat selection on the diversity of macrobenthic communities in three gulfs of the Cuban Archipelago. Bull Mar Sci. 2018;94: 249–68. doi: 10.5343/bms.2017.1013.

[29] Côté IM, Knowlton N. Coral reef ecosystems: a decade of discoveries. In: Bertness MD, Bruno JF, Silliman BR, Stachowicz JJ, editors. Marine community ecology and conservation. Sunderland: Sinauer Associates; 2013. Pp. 299–314.

[30] Duffy JE, Hughes AR, Moksnes P-O. Ecology of seagrass communities. In: Bertness MD, Bruno JF, Silliman BR, Stachowicz JJ, editors. Marine community ecology and conservation. Sunderland: Sinauer Associates; 2013. Pp. 271–297.

[31] Caballero Aragón H, Armenteros M, Perera Valderrama S, Rey Villiers N, Cobián Rojas D, Campos Verdecia K, Alcolado Menéndez PM. Ecological condition of coral reef assemblages in the Cuban Archipelago. Mar Biol Res. 2019;15: 61–73. doi: 10.1080/17451000.2019.1577557.

[32] Martínez-Daranas B, Suárez AM. An overview of Cuban seagrasses. Bull Mar Sci. 2018;94: 269–282. doi: 10.5343/bms.2017.1014.

[33] Hoskins CW. Molluscan biofacies in calcareous sediments, Gulf of Batabanó, Cuba. Bull Am Assoc Pet Geol. 1964;48: 1680–1704.

[34] Capetillo-Piñar N, Villalejo-Fuerte MT, Tripp-Quezada A. Distinción taxonómica de los moluscos de fondos blandos del Golfo de Batabanó, Cuba. Lat Am J Aquat Res. 2015;43: 856–72. Doi: 10.3856/vol43-issue5-fulltext-6.

[35] Armenteros M, Díaz-Asencio M, Fernández-Garcés R, Eriksson M, Alonso-Hernández C, Sánchez-Cabeza J-A. Historical changes of sediments and mollusk assemblages in the Gulf of

Batabanó (Caribbean Sea) in the twentieth century. Environ Monit Assess. 2012;184: 4709–4723. Doi: 10.1007/s10661-011-2296-z.

[36] Armenteros, M., Díaz-Asencio, M., Fernández-Garcés, R., Hernández, C. A., Helguera-Pedraza, Y., Bolaños-Alvarez, Y., Agraz-Hernández, C., & Sanchez-Cabeza, J.-A. One-century decline of mollusk diversity as consequence of accumulative anthropogenic disturbance in a tropical estuary (Cuban Archipelago). Mar Pollut Bull. 2016;113:224–231. Doi: 10.1016/j.marpolbul.2016.09.023.

[37] Armenteros M, Díaz-Asencio M, Peraza-Escarrá R, Fernández-Garcés R, Martínez-Suárez A, Kenney WF, Brenner M. Mollusk death assemblages in ^{210}Pb-dated marine sediment cores reveal recent biotic changes in the Gulf of Guanahacabibes, NW Cuba. Mar Environ Res. 2021;171: 105477. Doi: 10.1016/j.marenvres.2021.105477.

[38] Buesa RJ. Annual benthic irradiance levels at the Northwestern Cuban shelf. Reg Stud Mar Sci. 2020;33: 100969. Doi: 10.1016/j.rsma.2019.100969.

[39] Abbott RT. American Seashells. 2nd ed. New York: Van Nostrand Reinhold; 1974. 663 p.

[40] Mikkelsen PM, Bieler R. Seashells of southern Florida. Living marine mollusks of the Florida Keys and adjacent regions. Bivalves. New Jersey: Princeton University Press; 2008.

[41] Tunnell JW, Andrews J, Barrera NC, Moretzson F. Encyclopedia of Texas seashells: identification, ecology, distribution and history. Texas: Texas A&M University Press; 2010.

[42] Redfern C. Bahamian seashells: 1161 species from Abaco, Bahamas. Bahamian seashells. Com, Incorporated; 2013.

[43] Todd J. Bivalves; 2008. Gastropods; 2006. Database: Neogene Marine Biota of Tropical America (NMITA) [Internet]. Available from: https://nmita.rsmas.miami.edu/index.htm

[44] Rosenberg G, Moretzsohn F, García EF, Felder DL, Camp DK. Gastropoda (Mollusca) of the Gulf of Mexico. In: Felder DL, Camp DK, editors. Gulf of Mexico origins, waters, and biota, Vol. 1. Texas A&M University Press; 2009. Pp. 579–699.

[45] Turgeon DD, Lyons WG, Mikkelsen P, Rosenberg G, Moretzsohn F, Felder DL, Camp DK. Bivalvia (Mollusca) of the Gulf of Mexico. In: Felder DL, Camp DK, editors. Gulf of Mexico origins, waters, and biota, Vol. 1. Texas A&M University Press; 2009. Pp. 711–744.

[46] Hill MO. Diversity and evenness: a unifying notation and its consequences. Ecology 1973;54: 427–32. Doi: 10.2307/1934352.

[47] Hsieh TC, Ma KH, Chao A. iNEXT: an R package for rarefaction and extrapolation of species diversity (Hill numbers). Methods Ecol Evol. 2016;7: 1451–1456. Doi: 10.1111/2041-210X.12613.

[48] Faith DP. Conservation evaluation and phylogenetic diversity. Biol Conserv. 1992;61: 1–10. Doi: 10.1016/0006-3207(92)91201-3.

[49] Cardoso P, Rigal F, Carvalho JC. BAT – Biodiversity Assessment Tools, an R package for the measurement and estimation of alpha and beta taxon, phylogenetic and functional diversity. Methods Ecol Evol. 2015;6: 232–236. Doi: 10.1111/2041-210X.12310.

[50] Petchey OL, Gaston KJ. Functional diversity (FD), species richness and community composition. Ecol Lett. 2002;5: 402–411. Doi: 10.1046/j.1461-0248.2002.00339.x.

[51] Legras N, Loiseau N, Gaertner J-C. Functional richness: Overview of indices and underlying concepts. Acta Oecol. 2018;87: 34–44. Doi: 10.1016/j.actao.2018.02.007.

[52] Streit RP, Bellwood DR. To harness traits for ecology, let's abandon 'functionality.' Trends Ecol Evol. 2022;3084: 1–10. Doi: 10.1016/j.tree.2022.11.009.

[53] Cardoso P, Rigal F, Borges PAV, Carvalho JC. A new frontier in biodiversity inventory: A proposal for estimators of phylogenetic and functional diversity. Methods Ecol Evol. 2014;5: 452–461. Doi: 10.1111/2041-210X.12173

[54] Cardoso P, Borges PAV, Veech JA. Testing the performance of beta diversity measures based on incidence data: the robustness to undersampling. Divers Distrib. 2009;15: 1081–1090. Doi: 10.1111/j.1472-4642.2009.00607.x.

[55] Clarke KR, Gorley RN, Somerfield PJ, Warwick RM. Change in marine communities: an approach to statistical analysis and interpretation. 3rd ed. Plymouth: PRIMER-E: Plymouth; 2014. 262 p.

[56] Anderson MJ, Gorley RN, Clarke KR. PERMANOVA+ for PRIMER: Guide to software and statistical methods. Massey: PRIMER-E Ltd; 2008. 218 p.

[57] Wickham H, Averick M, Bryan J, Chang W, McGowan LD, François R, Grolemundet G, et al. Welcome to the Tidyverse. J Open Source Soft. 2019;4: 1686. doi: 10.21105/joss.01686.

[58] Gotelli NJ, Colwell RK. Quantifying biodiversity: procedures and pitfalls in the measurement and comparison of species richness. Ecol Lett. 2001;4: 379–391. doi: 10.1046/j.1461-0248.2001.00230.x.

[59] van der Plas F, Hennecke J, Chase JM, van Ruijven J, Barry KE. Universal beta-diversity – functioning relationships are neither observed nor expected. Trends Ecol Evol. 2023. doi: 10.1016/j.tree.2023.01.008.

[60] Costello MJ, Tsai P, Wong PS, Cheung AKL, Basher Z, Chaudhary C. Marine biogeographic realms and species endemicity. Nature Comm. 2017;8: 1057. doi: 10.1038/s41467-017-01121-2.

[61] Huston M. Biological diversity — The coexistence of species. Cambridge University Press; 1994. 681 p.

[62] Lluis-Riera M. Estudios hidrológicos del Golfo de Batabanó y de las aguas oceánicas adyacentes. Ser Oceanol. 1972;14: 1–46.

[63] Arriaza L, Rodas L, Simanca J, Lorenzo-Sánchez S, Milian DL, Romero P. Contribución a la gestión ambiental del Golfo de Batabanó, Cuba: Modelación numérica de corrientes marinas. Rev Invest Mar. 2008;29: 89–99.

[64] Baisre J. An overview of Cuban commercial marine fisheries: The last 80 years. Bull Mar Sci. 2018;94: 359–375. doi: 10.5343/bms.2017.1015.

[65] Altieri AH, Johnson MD, Swaminathan SD, Nelson HR, Gedan KB. Resilience of tropical ecosystems to ocean deoxygenation. Trends Ecol Evol. 2021;3: 227–238. doi: 10.1016/j.tree.2020.11.003.

[66] Virta L. Diversity of benthic diatoms in the Baltic Sea: alpha and beta diversity, environmental drivers, and diversity-biomass relationships. Mar Ecol Progr Ser. 2023;712: 35–47. doi: 10.3354/meps14320.

[67] Zuschin M, Ebner C. Compositional fidelity of death assemblages from a coral reef-associated tidal-flat and shallow subtidal lagoon in the northern Red Sea. Palaios. 2015;30(3): 181–191. doi: 10.2110/palo.2014.032.

[68] Bracchi VA, Meroni AN, Epis V, Basso D. Mollusk thanatocoenoses unravel the diversity of heterogeneous rhodolith beds (Italy, Tyrrhenian Sea). Diversity. 2023;15: 526. doi: 10.3390/d15040526.

[69] Barrientos-Luján NA, Rodríguez-Zaragoza FA, and López-Pérez A. Richness, abundance and spatial heterogeneity of gastropods and bivalves in coral ecosystems across the Mexican tropical Pacific. J Molluscan Stud. 2021;87: eyab004. doi: 10.1093/mollus/eyab004.

[70] Hemminga MA, Duarte CM. Seagrass ecology. Cambridge University Press; 2000. 298 p.

[71] Kidwell SM. Ecological fidelity of open marine molluscan death assemblages: effects of post-mortem transportation, shelf health, and taphonomic inertia. Lethaia. 2008;4: 199–217. doi: 10.1111/j.1502-3931.2007.00050.x.

[72] Davis KA, Pawlak G, Monismith SG. Turbulence and coral reefs. Annu Rev Mar Sci. 2021;13: 343–373. doi: 10.1146/annurev-marine-042120-071823.

[73] Albano PG, Sabelli B. Comparison between death and living molluscs assemblages in a Mediterranean infralittoral off-shore reef. Palaeogeogr Palaeoclimatol Palaeoecol. 2011;310: 206–215. doi: 10.1016/j.palaeo.2011.07.012.

[74] MacIntyre HL, Geider RJ, Miller DC. 1996. Microphytobenthos: The ecological role of the “secret garden” of unvegetated, shallow-water marine habitats. I. Distribution, abundance and primary production. Estuaries 1996;19: 186–201.

[75] Miller DC, Geider RJ, MacIntyre HL. 1996. Microphytobenthos: The ecological role of the "secret garden” of unvegetated, shallow-water marine habitats. II. Role in sediment stability and shallow-water food webs. Estuaries 1996;19: 202–212.

[76] Jiménez-Ramos, R., Egea, L. G., Vergara, J. J., Bouma, T. J., & Brun, F. G. (2019). The role of flow velocity combined with habitat complexity as a top–down regulator in seagrass meadows. Oikos, 128, 64–76. doi: 10.1111/oik.05452.

[77] Witman JD, Etter RJ, Smith F. The relationship between regional and local species diversity in marine benthic communities: A global perspective. Proc Nat Acad Sci USA. 2004;101: 15664–15669. doi: 10.1073/pnas.0404300101.

[78] Hewitt JE, Thrush SF, Halliday J, Duffy C. The importance of small-scale habitat structure for maintaining beta diversity. Ecology 2005;86: 1619–1626. doi: 10.1890/04-1099.

[79] Creed JC, Kinupp M. Small scale change in mollusk diversity along a depth gradient in a seagrass bed off Cabo Frio, (Southeast Brazil). Braz J Oceanogr. 2011;59: 267–276.

[80] Davis T, Harasti D, Kelaher B, Smith SDA. Spatial and temporal variation in subtidal molluscan diversity amongst temperate estuarine habitats. Mar Ecol. 2017; 38: e12428. doi: 10.1111/maec.12428.

Supporting Information

S1 Table. **Matrix of biological traits of mollusk species.**

S2 Table. **Matrix of mollusk species per sample.** The replicates are denoted as R1–3.

S3 Fig. Species richness and β-diversity accumulation curves. (a) Regional species richness. (b) Regional β-diversity. (c) Habitat species richness. (d) Habitat β-diversity. (e) Local species richness Dots indicate the observed richness, shaded areas the 0.95 CIs.

S4 Fig. Regional composition. Heat map of relative abundance (in %) of those typical species that contribute to the 70% of similarity within each gulf. Note that species were ordered by their similarity across the gulfs.

S5 Table. Results of the Permutational Analysis of Variance (PERMANOVA).

Figure 1

Click here to access/download;Figure;Fig_1_Map.tif

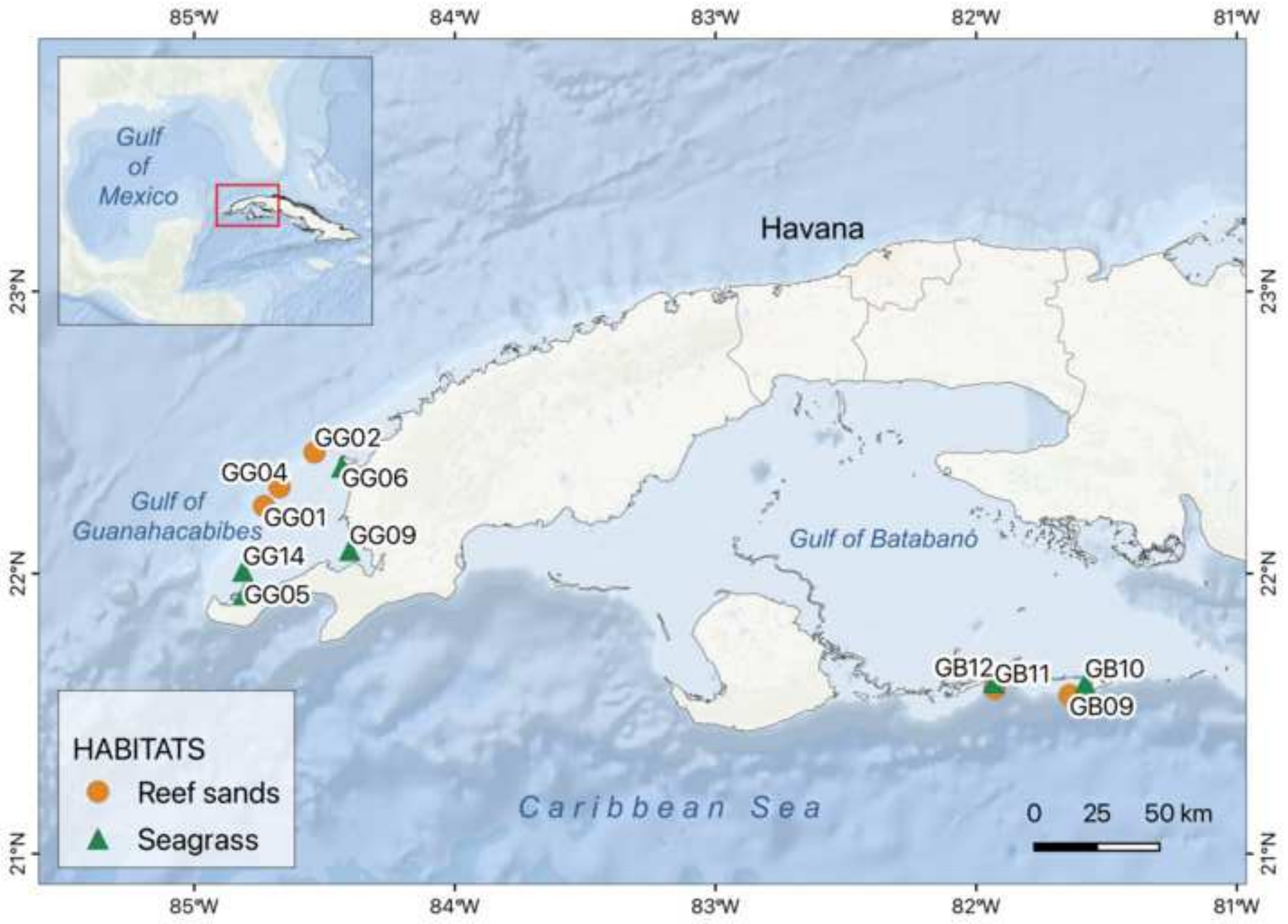

Figure 2

Click here to access/download;Figure;Fig_2_Gulf_Alpha_Beta.tif

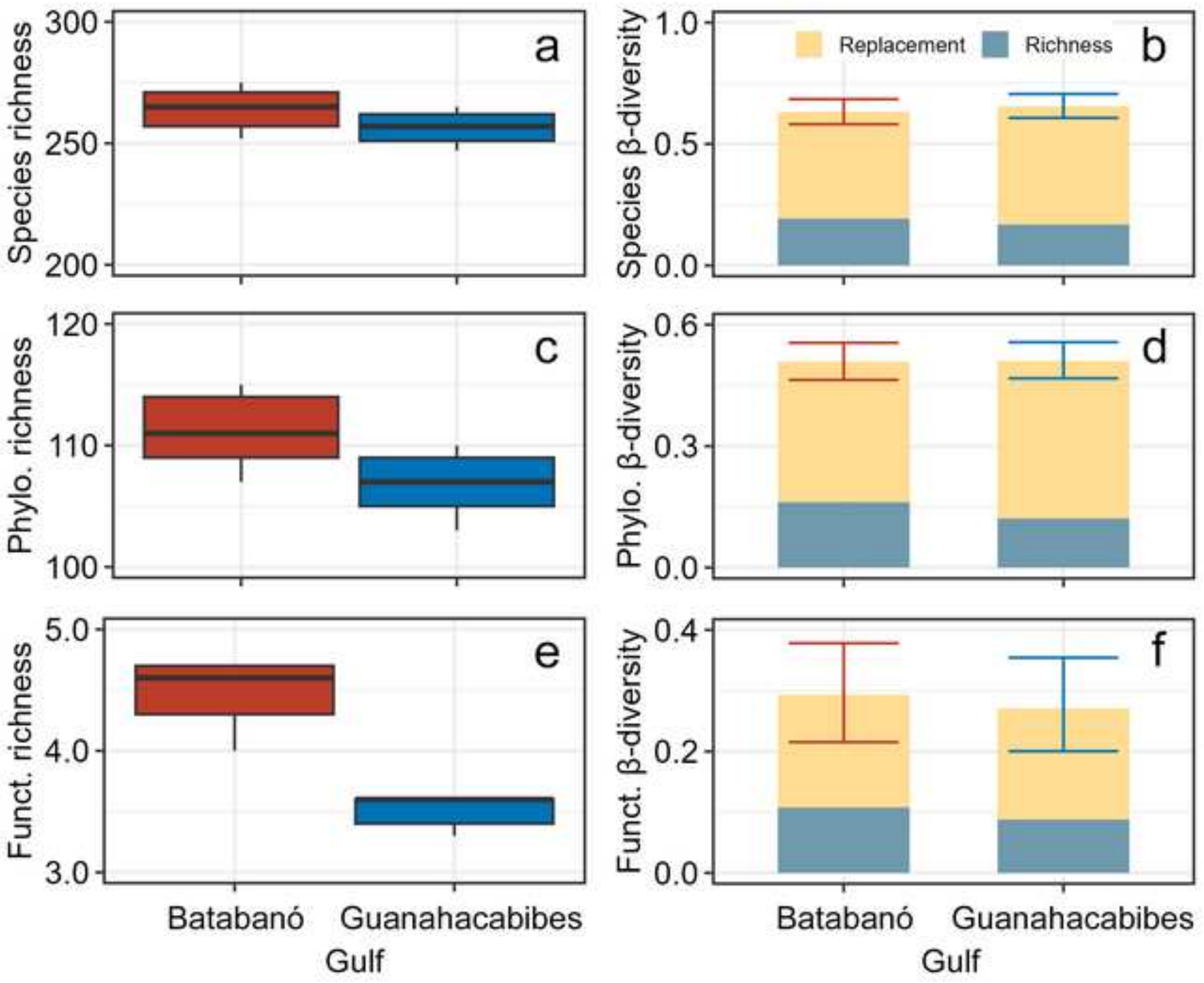

Figure 3

Click here to access/download;Figure;Fig_3_Dominance_curves.tif

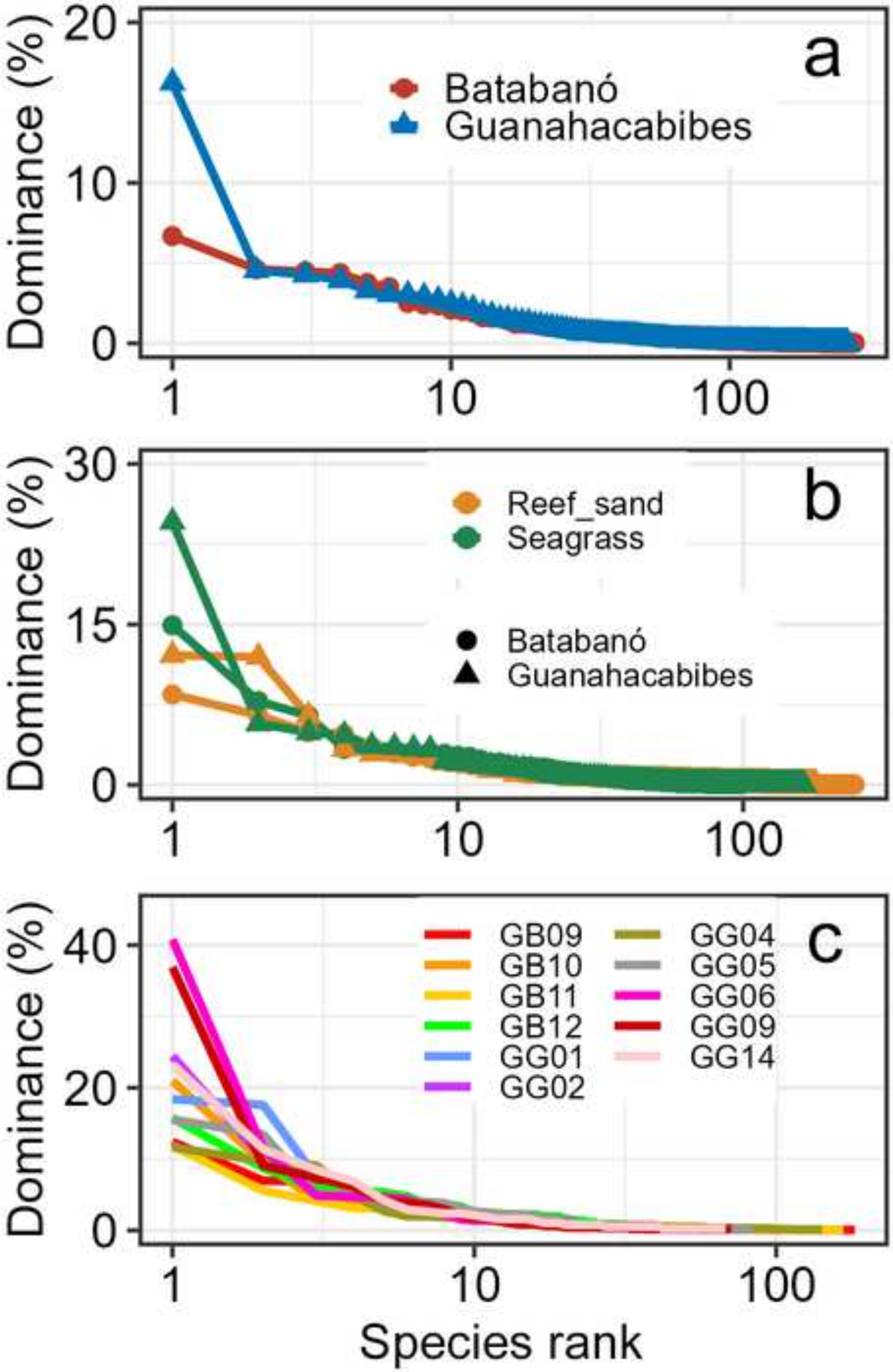

Figure 4

Click here to access/download;Figure;Fig_4_Gulfs_traits.tif

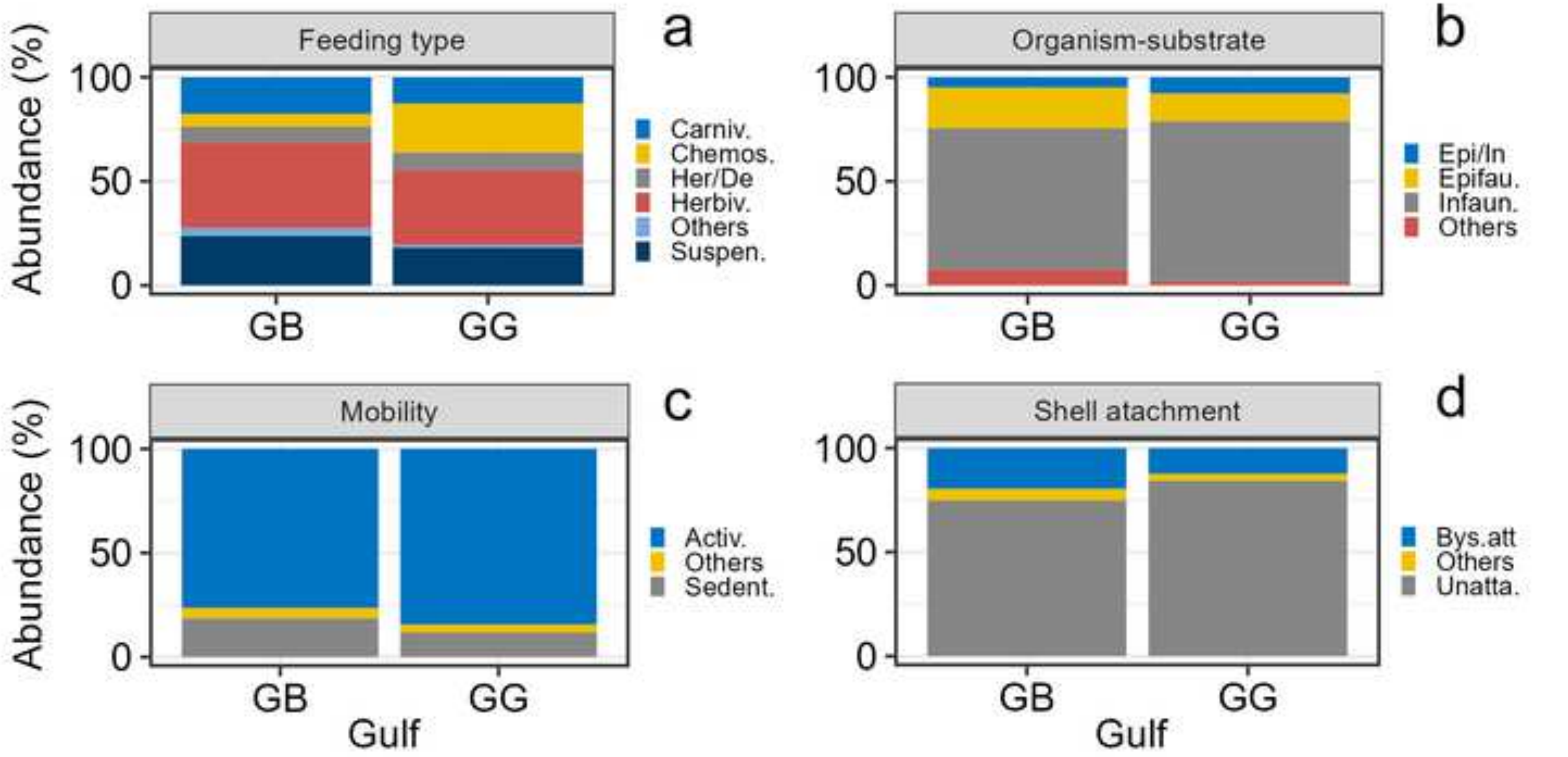

Figure 5 Click here to access/download;Figure;Fig_5_Habitat_Alpha_Beta.tif

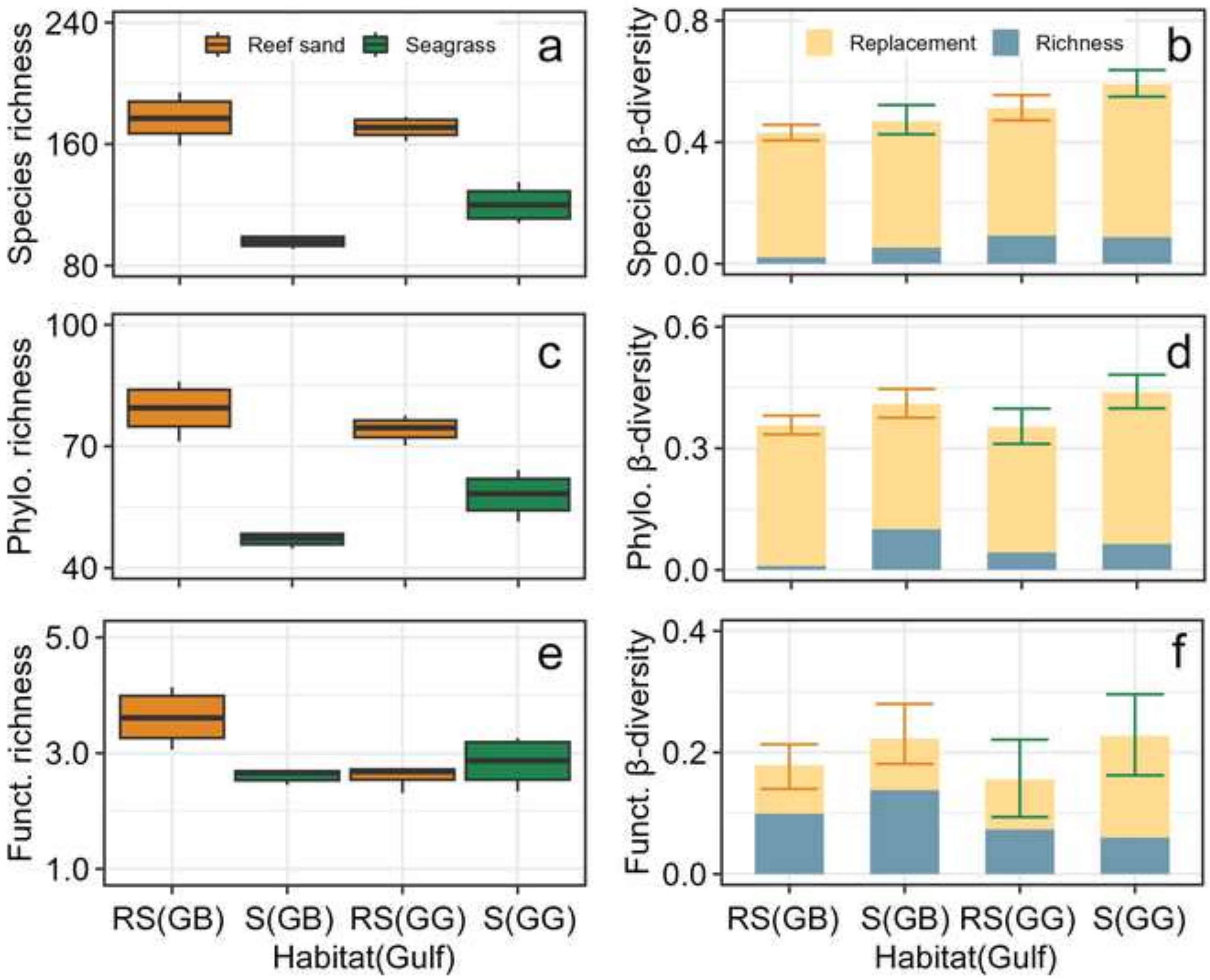

Figure 6

Click here to access/download;Figure;Fig_6_Habitat_heatmap.tiff

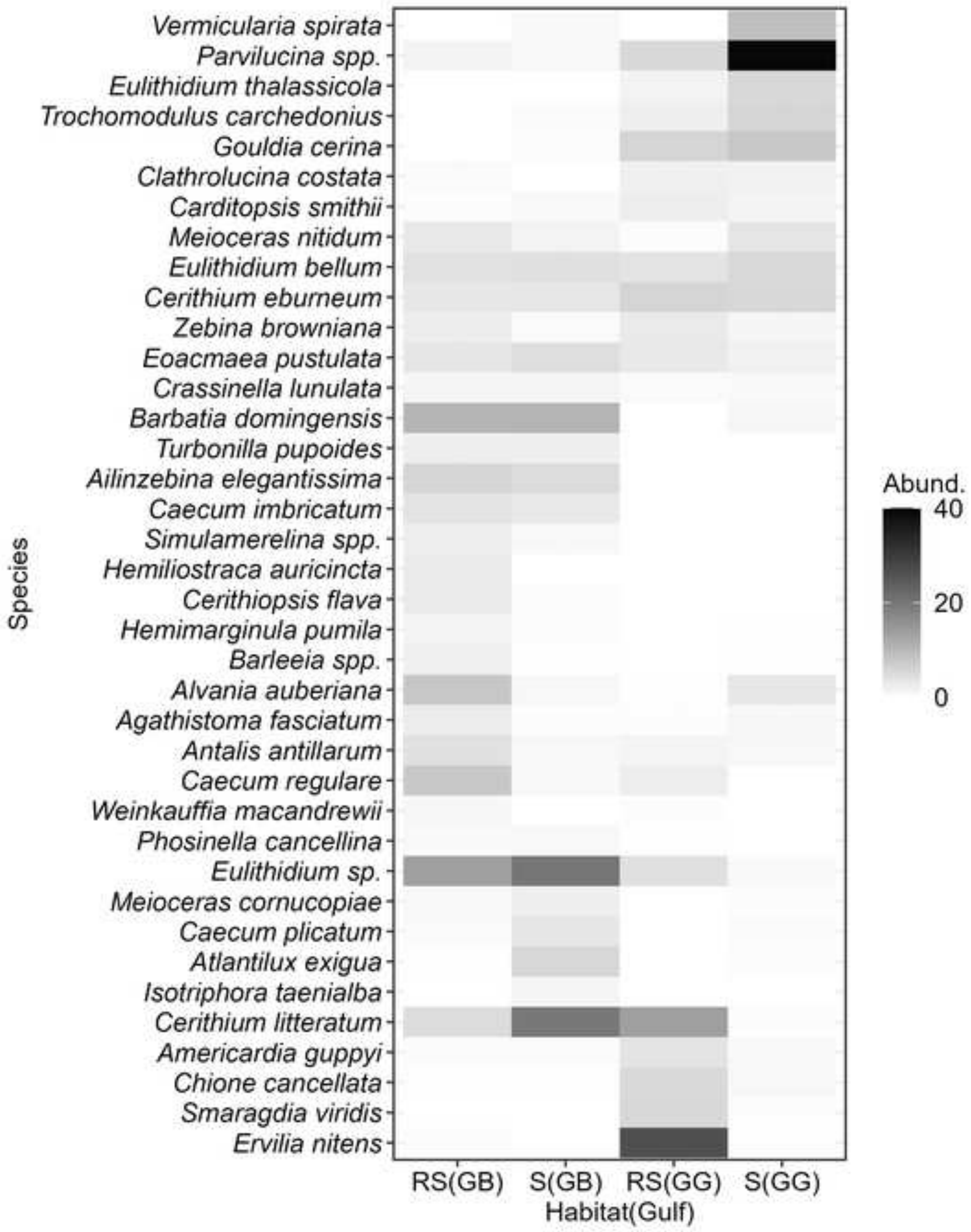

Figure 7

Click here to access/download;Figure;Fig_7_Habitats_traits.tif

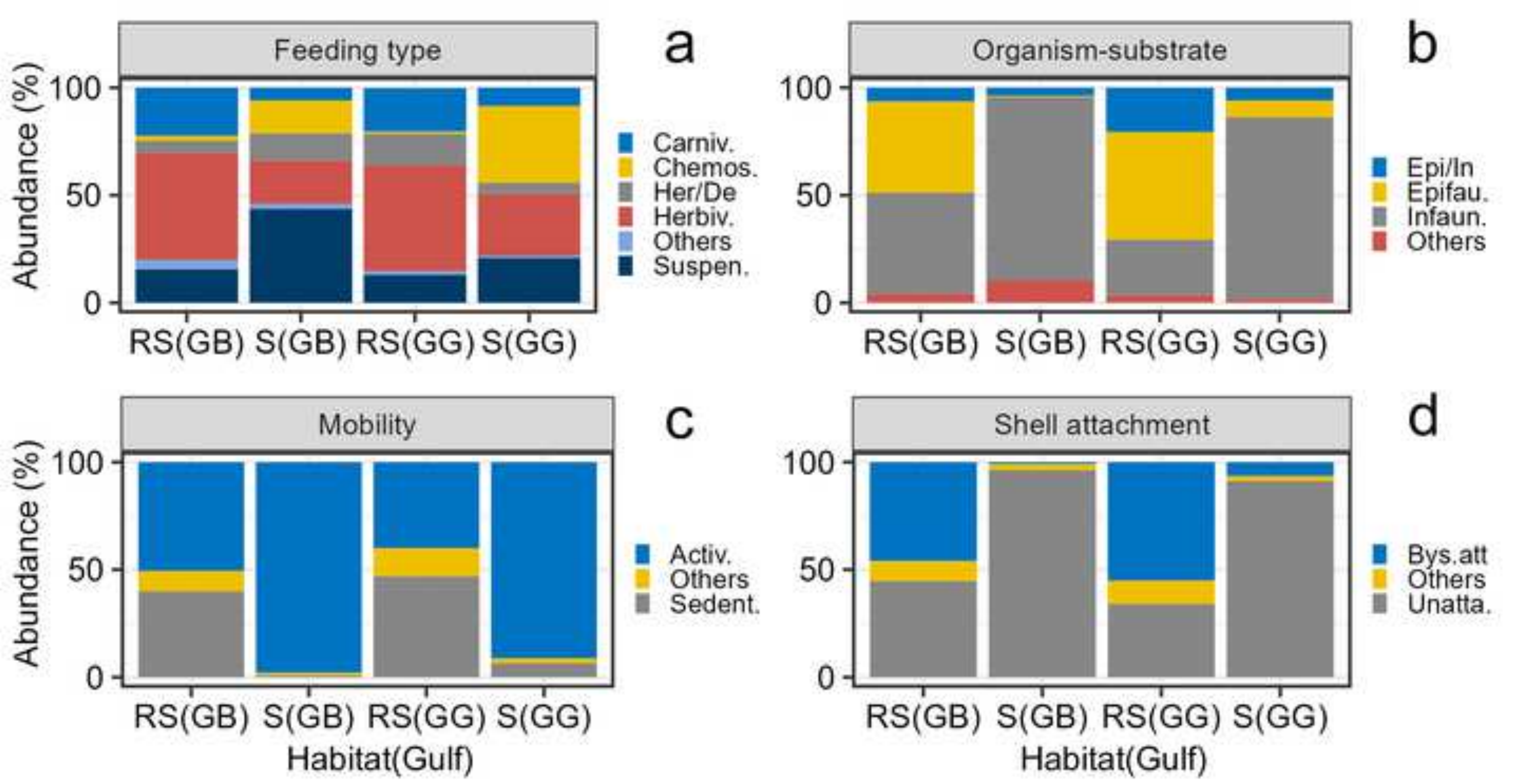

Figure 8

Click here to access/download;Figure;Fig_8_Site_Alpha_beta.tif

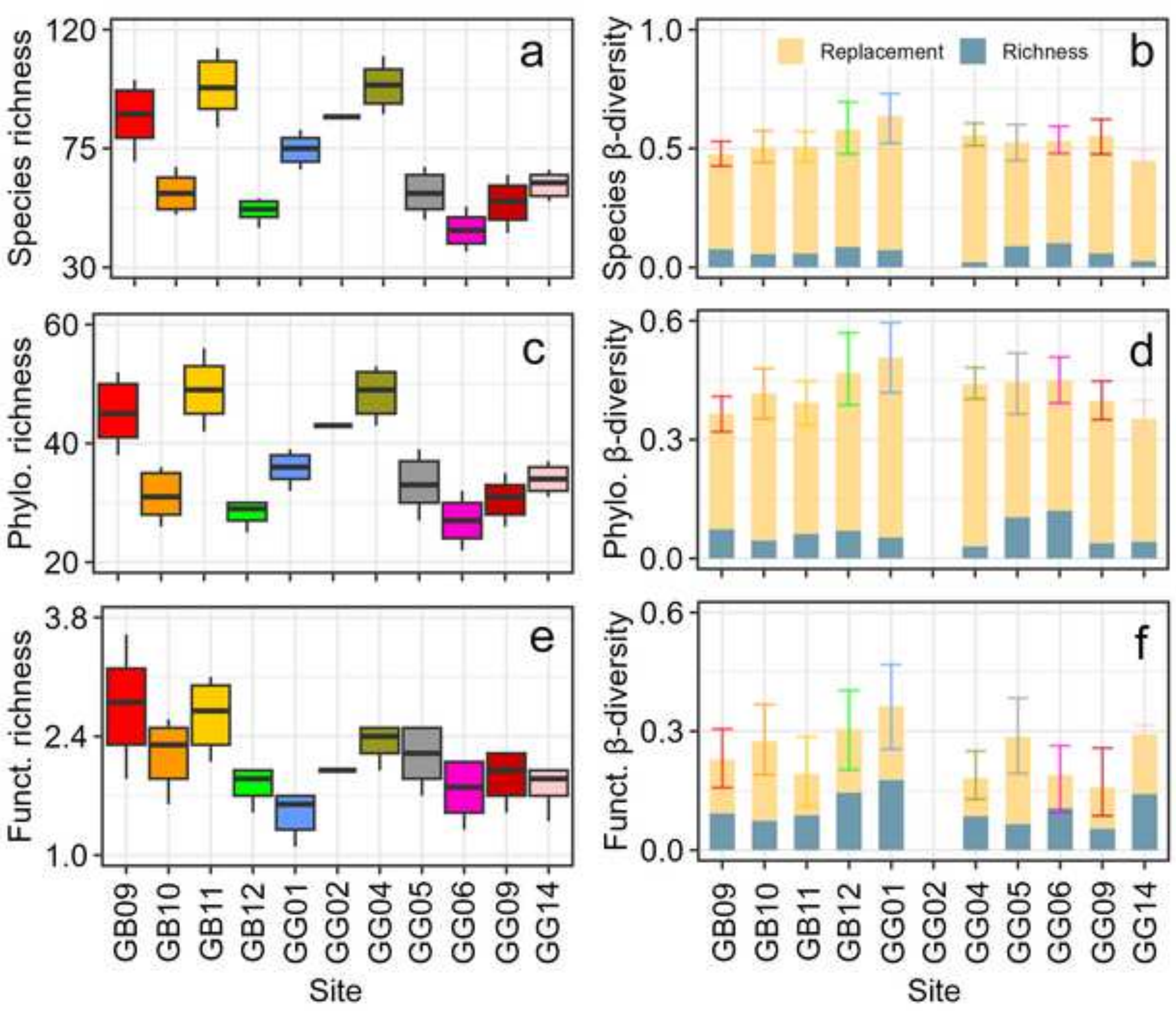

Figure 9

Click here to access/download;Figure;Fig_9_MDS_sites.tif

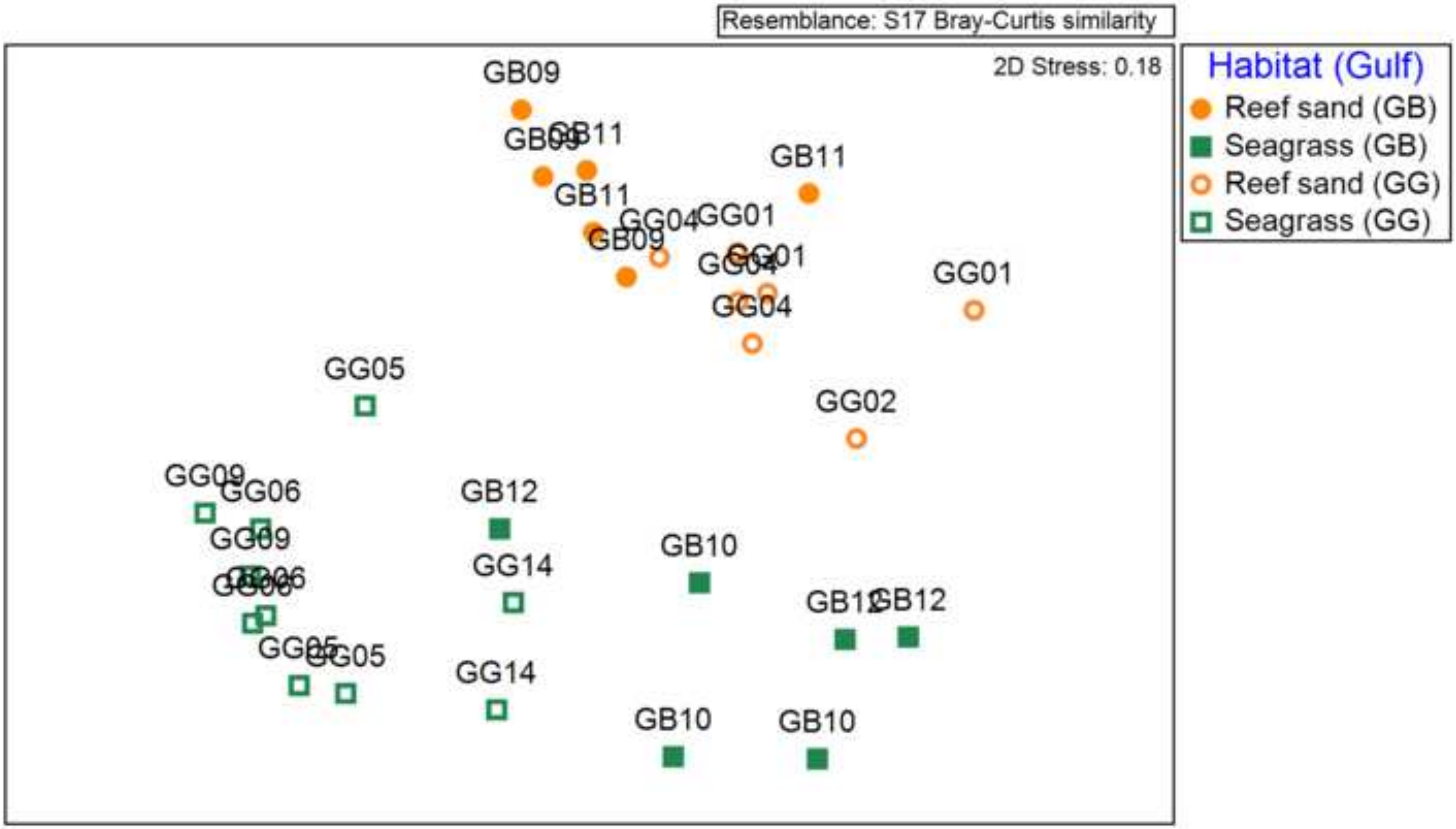

Figure 10

Click here to access/download;Figure;Fig_10_Sites_Traits.tif

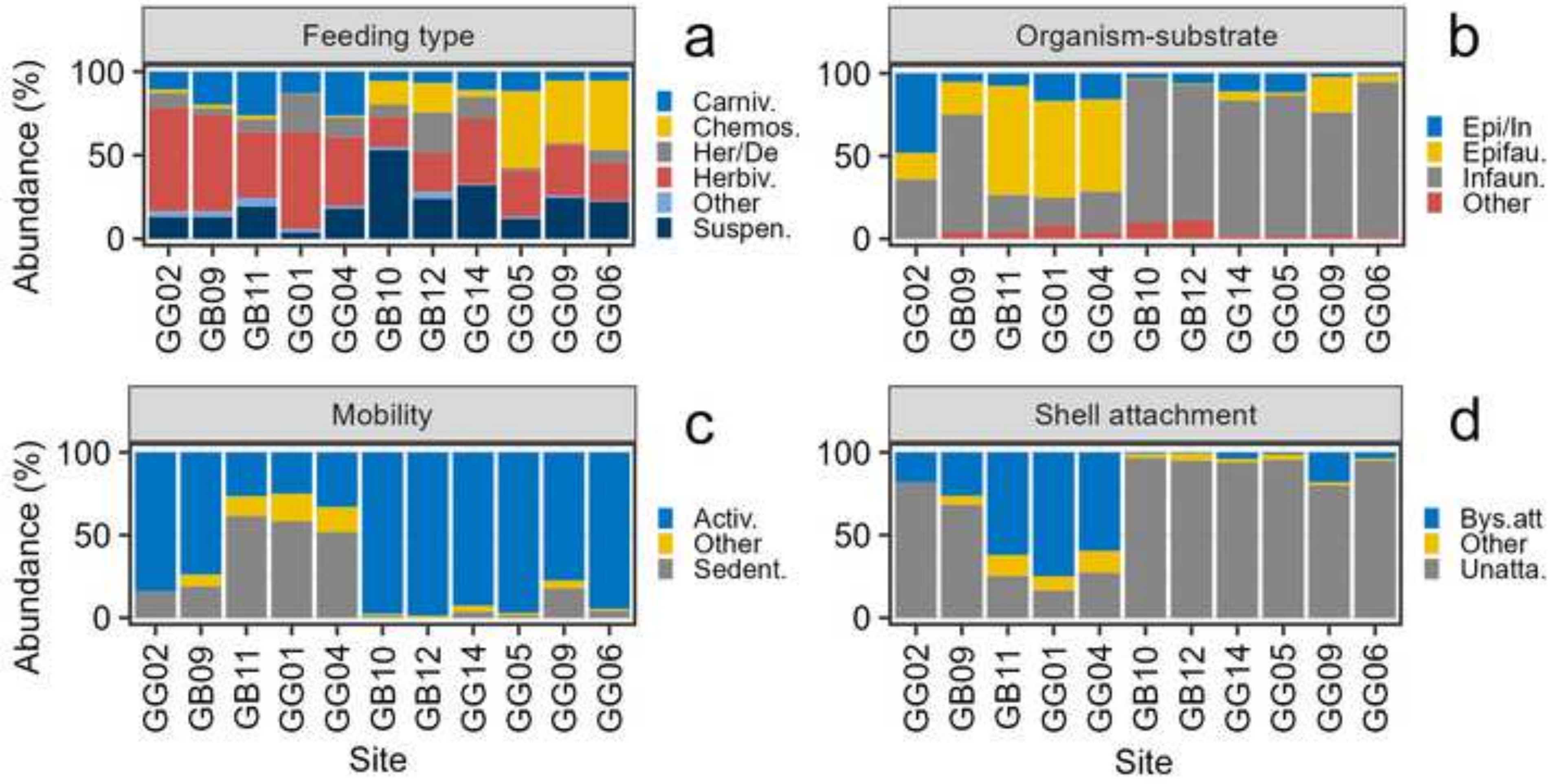

Click here to access/download
Supporting Information
Supporting_Information_S1_Table_Traits.xlsx

Click here to access/download
Supporting Information
Supporting_Information_S2_Table_Abundance.xlsx

Click here to access/download
Supporting Information
Supporting_Information_S3_Fig _Curves.tif

Click here to access/download
Supporting Information
Supporting_Information_S4_Fig_Heatmap_regions.tiff

Click here to access/download
Supporting Information
Supporting_Information_S5_Table_PERMANOVA results.docx

~~Title page~~

~~Full Title: ~~Taxonomic, phylogenetic, and functional ~~D~~diversit~~y~~yies ~~patterns ~~of mollusk death assemblages in coral ~~coral ~~reef~~s~~ and seagrass ~~meadows ~~sediments from two shallow gulfs in western Cuban archipelago

~~he gGulfs of Batabanó (westernNW Caribbean) and Guanahacabibes (SE Gulf of Mexico)two Batabanócuban gulfs in the western Cuba (Caribbean Sea and): Batabanó and Guanahacabibeshatwo gulfs infrom the shallow (two gulfswestern Caribbean Sea) and Guanahacabibes (the southeastern Gulf of Mexico). and a comparison with living assemblages~~

Short title: Diversity patterns of mollusk death assemblages

~~Authors: ~~Rosely Peraza-Escarrá[1,2*], Maickel Armenteros[2], Raúl Fernández-Garcés[3], Adolfo Gracia[2]

~~Affiliations:~~

[1] Posgrado de Ciencias del Mar y Limnología, Universidad Nacional Autónoma de México, Ciudad de México, México.

[2] Instituto de Ciencias del Mar y Limnología, Universidad Nacional Autónoma de México, Ciudad de México, México.

Formatted: Font: Not Bold

Formatted: Centered

Formatted: Font: 14 pt

Formatted: Strikethrough

Formatted: English (United States)

[3] Centro de Estudios Ambientales de Cienfuegos, Ciudad de Cienfuegos, Cuba.

* Corresponding author

Email: roselyperaza2014@gmail.com

Abstract

Mollusks are recognized for their huge diversity and their shell remnants (death assemblages) provide valuable ecological insights. ~~Although, d~~Diversity patterns in tropical marine habitats ~~and links between dead and live sympatric assemblages~~ still are understudied; ~~. Th~~therefore we aimed to ~~(1)~~ investigate the taxonomic, phylogenetic, and functional diversit~~ies~~ y of mollusk death assemblages at regional and local scales in coral reef sands and seagrass meadows~~; (2) evaluate death assemblages as surrogate of the whole mollusk assemblages (alive + dead); and (3) explore the downslope transport of shelf species to the deep sea~~. We collected sediment samples at 11 sites ~~from~~ in ~~coral~~ reef sands and seagrass within two shallow gulfs (Batabanó in the Caribbean Sea and Guanahacabibes in the southeastern Gulf of Mexico). All the shells were counted and identified to species level and classified into biological traits. We identified 7113 individuals belonging to 393 species. Diversity and assemblage structure between gulfs showed many similarities given their geological and geographical commonalities. Reef sands had higher richness than seagrasses likely because of a more favorable productivity-disturbance balance. ~~Coral r~~Reef sands were dominated by epifaunal herbivores feeding on microphytobenthos and bisally attached bivalves adapted to intense hydrodynamic regime. In seagrass meadows, suspensivores dominated in exposed sites and chemosynthetic infaunal bivalves dominated in sites where oxygen replenishment was limited. Time averaging of death assemblages was likely in the order of 100 years in our samples, with stronger effects in reef sands compared to seagrass meadows. ~~Time averaging of the death assemblages~~ Our research provides evidence of the outstanding taxonomic and functional diversity of death mollusk assemblages in tropical coastal sediments as resulted of the influence of scale-related processes and ~~the~~ habitat type.~~Two seagrass sites, closer to the coastline, showed evidence of eutrophication and sedimentation impacts. Death assemblages contained a substantial fraction of~~

Formatted: Font: 18 pt

Formatted: Font color: Red

Formatted: Font color: Red

Formatted: Font color: Auto

Formatted: Font color: Auto

Formatted: Font color: Auto

Formatted: Font color: Auto

Formatted: Font color: Auto

Formatted: Font color: Red

~~the sympatric whole assemblage supporting its value for diversity and structure assessment. The nature of the habitat probably influenced the time averaging as reflected by the lower dead-to-whole assemblage contribution in seagrass sites compared to reefs. There was evidence of shelf-to-slope transport, but β-diversity patterns and distinctive assemblage structure suggested that lateral transport of dead shells was rather limited. Distance was a significant predictor of richness of shelf species in the deep sea. The potential downslope transport of dead shells and pollutants from coastal areas deserves further studies.~~

Formatted: Font color: Red

Keywords: diversity, mollusk death assemblage, ~~thanatocoenoksis~~thanatocoenosis, ~~postmortem transport,~~ Gulf of Mexico, Caribbean Sea.

Introduction

Formatted: Font: 18 pt

Formatted: Font: 20 pt

Death assemblages are defined as the taxonomically identifiable skeletal remains from recent and past generations that are present in the surficial mixed layer on landscapes and seafloors [1, (Kidwell 2013, Kidwell and Tomašových 2013) 2]. Therefore, death assemblages may include refer to taxa with hard structures such as Mollusca, Foraminifera and Echinodermata. Studies about diversity and community ecology structure of death assemblages provide historical baselines (e.g., Tomašových et al. 2023[3]) but also insights about modern spatial patterns (e.g., Casebolt and Kowalewski 2018[4]). Some taphonomic processes may obscureinfluence the signal diversity and ecological structure of death assemblages, being the most important the time averaging and the postmortem lateral transport of shells. Time averaging is the co-occurrence of skeletal remains of individuals that died in different times reducing the temporal resolution of the study [2](Kidwell and Tomašových 2013). However, a positive point is that time averaging allows death assemblages to reach higher species richness than any single census of their corresponding living assemblage and displays a better mapping of communities and habitat types (Kidwell and Bosence 1991)[5]. On the other hand, the literature suggests that the lateral transport of skeletal remains in bottom sublittoral environments is rather small because (Kidwell and Bosence 1991)[5]: lateral movement of shells is mainly within-habitat, most allochthonous shells are from immediately adjacent habitats, and drift transport affects particularly small thin shells. We focus here on Mollusca, which together with Foraminifera and Echinodermata are the most studied Ddeath assemblages may refer to taxa with hard structures such asin the marine realm, and in this study we focus on the first taxonof themmollusks Mollusca, Foraminifera and Echinodermata.

Mollusca is the 2nd most diverse phylum of metazoans (Zhang 2013)[6] with about 133000 described species (WoRMS 2023)[7]. Most mollusks bear a shell of calcium carbonate that lasts in

the sediments longer after the dead of the individual providing a rich source of information about diversity. Especially in marine ecosystems, mollusk death assemblages are a promising source of historical data since the analyses of shells provides knowledge about community structure (e.g., richness, abundance, evenness, composition) and functional features (e.g., trophic guildsproductivity, bioturbation potential) ([1, 8-, Grill and Zuschin 2001, Tomašových and Kidwell 20099, Kidwell 2013, Weber and Zuschin 201310, Peraza-Escarrá et al. 202211)]. Therefore, mollusk death assemblages constitute a powerful and rich-information tool to analyze diversity and to stablishes biological baselines; particularly in those ecosystems where sampling and knowledge is scarce or null, like the southern of the Gulf of Mexicowestern tropical Atlantic.

To study mollusk assemblages some methodological issues should be addressed given that diversity is a complex concept that includes scales in space and time, and entities such as species, traits, and evolutionary units (Pavoine and Bonsall 2011)[12]. Within the last ten years has occurred an accelerate development of approaches and metrics for measuring diversity ([13, 14Magurran 2004, 2021)]. Magurran (2021)[14] highlights the power of Robert Whittaker's approach that uses complementary facets of diversity (i.e., richness and compositional change) and levels (α-, β-, and γ-diversities). This approach extends to the analysis not only of species (or taxonomic) diversity, but also includes phylogenetic and functional diversities (Magurran 2021)[14]. Tucker et al. (2017)[15] and Mammola et al. (2021)[16] revised the plethora of phylogenetic and functional metrics, respectively, and proposed guidelines for the selection of appropriate metrics. For the analysis of compositional change (β-diversity *sensu lato*), in an outstanding synthesis, Anderson et al. (2011)[17] proposed the use of turnover and variation for directional and non-directional compositional changes, respectively; they also highlight the power of multivariate measures for β-diversity assessment. By other side, the partition of β-diversity patterns has been a controversial issue based on ecological and mathematical grounds [18(Legendre 2014)]: An approach splits β-

diversity in replacement and richness differences (Carvalho et al. 2012[19-, 2013,20, Cardoso et al. 2014b21)]; while a second approach use replacement and nestedness [22-, 23, (Baselga 2010, 2012, Baselga and Leprieur 2015)24]. When the analysis of species abundance of species is important, is Latter The eEvenness of the community(i.e., is a measure of how similar species are in their abundance) also constitutes a key metric of the community when changes in species relative the abundance is within the focus of interest (Magurran 2004)[13] and constitutes a key metric of the community structure. Recently, other two unified frameworks for the analyses of diversity that include the relative abundance have been proposed: Pavoine and Bonsall (2011)[12] proposed three types of overall measures (i.e., richness, skewness, and divergence) and Chao et al. (2014a)[25] proposed Hill's numbers for measuring richness and differentiation. Given the bewildering array of approaches for describing diversity, we have choosechosen some of the above methods that better fit to our objectives and type of data.

Formatted: English (United States)

Formatted: English (United States)

Habitat type is a tenet of benthic ecology affecting diversity and community structure and function [26, (Southwood 1977, Ferraro 2013)27]. Habitat type is a main driver of the diversity and assemblage structure of alive mollusks at local and regional scales (Armenteros et al. 2018)[28]. Therefore, we use the habitat type (coral reef and seagrass) as an organizing factor in current study of mollusk death assemblages in the western Cuban shelf. Specifically, Here we focus on sediments from coral reefs and seagrass meadows, two iconic habitats of tropical shelves harboring a huge diversity and complex communities [29, (Côté and Knowlton 2013, Duffy et al. 2013, Côté and Knowlton 2013)30]. Coral reefs cover extent over and important extension of Cuban archipelago and display a large spatial variability regarding cover, average coral size, and health and levels of impact (Caballero Aragoón et al. 2019)[31]. Given that most of coral reefs surfacesubstrate is hard bottom, we selected the coral sandy spots located in the spur-and-groove biotope within the reef (hereafter "reef sands"). to compare an are Seagrass meadows also also are key coastal ecosystems

withcoveroccupy also a -broad extension inof soft bottoms in Cuban shelf and play key roles such as biodiversity hot-spot and carbon storage (Martínez-dDaranas et al. 2018)[32]. Therefore, we compare here the sediment assemblages occurred inbetween the two both habitat types.

We compare Given tThe very different architecture of coral reefs (mostly hard substrate) and seagrass meadows (mostly muddy bottoms) makes difficult to compare , we selected the sediments To Both habitat types includesing important ecological functions such as carbon storage and roles as The diversitHowever, y of mollusk assemblages variabiliin … Seagrass meadows … Habitat type is a main driver of the diversity and assemblage structure of alive mollusks at local and regional scales (Armenteros et al. 2018). Therefore, we use the habitat type (coral reef and seagrass) as an organizing factor in current study of mollusk death assemblages in the western Cuban shelf.

In Cuban archipelago, mollusk diversity and community structure have been the subject of a few studies. Most of the mollusk diversity studies have been done in the Gulf of Batabanó, arguably the best-studied inner sea of the archipelago [28, 33, 34, (Hoskins 1964, Capetillo-Piñar et al. 2005, Armenteros et al. 2018]). Only one study has been done on the deep-sea mollusk assemblages (about 1500 m depth) in the northwestern slope, reporting a very high diversity and potential transport of shells from the shelf (Peraza-Escarrá et al. 2022)[11]. Other three studies using sediment dating with ^{210}Pb and ^{14}C reported historical changes in death mollusk assemblages as response to human disturbances [356, 37, -387] (Armenteros et al. 2012, 2016, 2021).

This study is part of a large project aimed to compare the benthic biology between inner seas (gulfs) in the western Cuban shelf, that encompass the southeastern Gulf of Mexico and the western Caribbean Sea. Given the background and knowledge gaps summarized above, Particularly for the gulfs of Batabanó and Guanahacabibes, we quantified the dead and live mollusks from a same sampling unit (box core) giving the opportunity to compare both fractions of the whole mollusk

assemblage across regions and habitats. This sort of comparison may support death assemblages as a surrogate of the whole diversity (Kidwell 2002, Smith 2008, Tomašových and Kidwell 2009). Tthe goal of our study is to document the -taxonomic, phylogenetic, and functional diversity of mollusk death assemblages in sediment environments within two types of habitats and at two spatial scales. We propose three specific objectivesGiven the background and knowledge gaps summarized above, we propose three objectives:

To investigate the diversity and structure of mollusk death assemblages : (i) at regional scale (Batabanó versus Guanahacabibes gulfs);).

To compare mollusk death assemblages , (ii) between habitats (coral reef sands sands versus seagrass meadow s).;, in coral reefs, we selected sediment (sandy) spots to compare with the soft bottoms in seagrass.comparabledirectly having in account that targeting th, and

1. To evaluate mollusk death assemblages among sites (iii) at local scale (i.e., among sites).As part of the diversity assessment,

2. For each To evaluate the death assemblages as surrogate of the whole mollusk assemblage (dead + live) collected from the same sites? and,

3. Are the shelf benthic species found in a deep-sea site off northwestern Cuba (site SL-38-750 at 1670 m depth) a remnant of the mollusk assemblages inhabiting the Gulf of Guanahacabibes?

The first specific objective, we analyzes the two components of diversity (richness and β-diversity) using the species, phylogenetic, and functional dimensions. The objectivesWe 1 also address the evenness and ecological structure (taxonomic/ and functional composition) of death assemblages; in other words, this part takes in accountconsiders the relative abundance of the species. Note that a phylogenetic ecological structurecomposition is not appropriate due to the nested nature of the

Formatted: Normal, No bullets or numbering

Formatted: Normal, Space Before: 0 pt, No bullets or numbering

Formatted: Normal, Space Before: 0 pt

Linnean hierarchy. The other two objectives are more specific and represent comparisons of shallow mollusk death assemblages with live (objective 2) and deep-sea (objective 3) assemblages.

Materials and methods

Formatted: Font: 18 pt

Study regions

Formatted: Font: 16 pt

We sampled two shallow seas (hereafter gulfs) in the western part of Cuban archipelago (Fig 1): Gulf of Batabanó (GB) in the northwestern Caribbean Sea and Gulf of Guanahacabibes (GG) in the southeastern Gulf of Mexico. Both gulfs have dominance of sedimentary bottoms, with shoreline covered by mangrove forests, extensive areas of seagrass meadows (mostly *Thalassia testudinum*) in the central part and bordered by coral reef tracts in the shelf border [365, (Armenteros et al. 2012, Buesa 2020)398]. Coral rReef sand sites in GB and GG are within the coral reef systems Canarreos and Los Colorados, respectively. Canarreos is a reasonably healthy reef system with the largest individual coral size and reef complexity of the whole Cuban archipelago (Caballero Aragoón et al. 2019)[31]. Meanwhile, Los Colorados contains a worrying number of depleted reefs with low living coral cover (Caballero Aragón et al. 2019)[31]. Seagrass sites differed in the proximity of the shelf border and therefore in the influence of open waters. GB sites, in the shelf border, were subjected to an intense physical disturbance by waving and currents coming from the Caribbean Sea and were relatively far from the pollution sources in the Cuba island. Meanwhile GG sites were farther away from the open ocean, with slower flushing of water by hydrodynamics and more exposed to anthropogenic disturbances such as eutrophication and turbidity (Armenteros et al. 2021)[387].

Fig 1. **Map of the study ~~regions~~regionsites**. Sampling sites within the ~~the Gulf~~gulfs of Batabanó (GB, ~~=~~ 4 sites) and ~~within the Gulf of~~ Guanahacabibes (GG, ~~=~~ 7 sites) are shown as circles and triangles, respectively. ~~Coral r~~Reefs~~Reefs~~ sands and ~~S~~seagrass meadows ~~and coral reefs~~ are ~~denoted~~ colored orange and green ~~and orange colors~~, respectively. Spatial data were visualized with QGIS v3.8~~D~~. ~~The deep-sea site SL-38-750 is indicated with a star.~~

Other similarities between GB and GG are the average depth (about 6–8 m) and the hydrological variables: temperature 27–29 °C, salinity 35.1–37.4 PSU, dissolved oxygen 6.1–8.0 mg L^{-1}, and pH 7.9–8.2. However, there are main differences between GB and GG, such as maximum depth (12 versus 28 m, respectively) and extension (20000 versus 1900 km^2).

Sampling and sample processing

We sampled 11 soft-bottom sites in two expeditions to GB (~~November,~~November 1st–5th, 2015) and GG (~~June~~May 30th–June 10th, ~~May~~ 2014) (Table 1). We selected two types of habitats broadly distributed across the gulfs: coral reefs and seagrass meadows. In coral reefs, we sampled in those ~~(i)~~ sandy spots within spur-and-groove structures in forereefs ~~(hereafter reef sand).~~ ~~(hereafter coral reef) and (ii) seagrass meadows (hereafter seagrass).~~ Therefore, we are dealing here with mollusk assemblages living in sediments (and not those assemblages on hard substrates ~~of the reef~~).

Formatted: Font: 16 pt

Formatted: Superscript

Formatted: Superscript

Formatted: Superscript

Formatted: Superscript

Table 1. Characterization of the sampling sites in the gulfs of Batabanó and Guanahacabibes. Abbreviations: GB = Gulf of Batabanó, GG = Gulf of Guanahacabibes, S+C = silt plus clay fractions, TOM = total organic matter, Reps. = number of replicates, N = abundance of dead individuals, S = number of species~~ in the mollusk death assemblage~~.

Gulf	Habitat	Site	Latitude (N)	Longitude (W)	Depth (m)	S+C (%)	TOM (%)	Reps.	N	S	Observations
GB	~~Coral r~~Reef sands	GB09	21°34.041	81°38.484	30	18	8	3	1553	181	“Acuario” buoy. Outer reef, clean waters, spur-and-groove biotope, fine and white sands
	Seagrass	GB10	21°36.527	81°34.959	3	26	10	3	744	79	Close to entrance channel of Cayo Largo marina. Meadow (high density) of *T. testudinum* and *S. filiforme*
	~~Coral r~~Reef sands	GB11	21°35.427	81°55.764	14	12	9	3	1118	166	Outer reef, clean waters, spur-and-groove biotope, fine and white sands
	Seagrass	GB12	21°36.744	81°06.016	4	55	9	3	355	55	Meadow (high density) of *T. testudinum* and algae
GG	R~~Coral r~~eef sands	GG01	22°14.397	84°43.933	20	-	8	3	364	83	Outer reef, clean waters, spur-and-groove biotope, many gorgonians and sponges, scarce sediment of coarse and white sands
	~~Coral r~~Reef sands	GG02	22°25.818	84°32.303	18	8	9	1	139	40	Outer reef, clean waters, spur-and-groove biotope, many gorgonians and sponges, scarce sediments, signs of strong hydrodynamics

	~~Coral r~~Reef sands	GG04	22°18.404	84°40.360	18	13	10	3	660	142	Outer reef, spur-and-groove biotope, many gorgonians and corals, more areas of sandy sediments than in previous sites
	Seagrass	GG05	21°55.556	84°48.483	4	43	13	3	752	83	Meadow composed mostly of *T. testudinum* (medium density) and many algae. Sediment gray and with white sand grains and darker in the top10 cm
	Seagrass	GG06	22°22.792	84°26.019	5	61	10	3	576	58	Meadow composed mostly of *T. testudinum* (medium density), many algae, muddy-sandy bottom rich in detritus, and many epiphytes on blades. Sediment is light gray along the entire core. Some shells >5 cm
	Seagrass	GG09	22°05.030	84°24.206	5	36	8	2	474	71	Meadow of *T. testudinum* (high density), muddy-sandy bottom with many invertebrates
	Seagrass	GG14	22°00.582	84°48.783	5	6	5	2	378	68	Meadow of *T. testudinum* (mid-low density) with short blades and many green algae
Total		11						29	7113	393	
~~Off GG~~	~~Continental slope~~	~~SL-38-750~~	~~22°28.735~~	~~84°41.568~~	~~1670~~	~~94~~	~~0.87~~	~~2, multi core~~			

Abbreviations: GB = Gulf of Batabanó, GG = Gulf of Guanahacabibes, S+C = silt plus clay fractions, TOM = total organic matter, Reps. = number of replicates, N = abundance, S = number of species. Data of the deep-sea site (SL-38-750) off the Gulf of Guanahacabibes are given as well..

Sediment samples~~ for mollusks~~ were collected by SCUBA divers using a box core (= sampling unit) with an effective sampling area of 100 cm^2. Usually, three sampling units were collected per site, but in three sites the replication number was lower (Table 1). The box core was pushed 10 cm into the bottom collecting approximately 1000 cm^3 of sediments. A potential caveat with this sampling is that it may miss ~~large~~deep burrowing species inhabiting below 10 cm deep. Sediments were sieved onboard with filtered seawater through a 500-µm sieve and preserved in 70% ethanol. We chose this mesh size to capture the micro-mollusks.~~ as well~~ In the laboratory, all the mollusk shells were sorted and counted under a stereomicroscope SZX7. In the case of the bivalves, each disarticulated valve was counted as an individual. Mollusk shells were also classified into (i) living individuals by the presence of soft body tissues, which were ~~(did~~ not included in this study~~)~~, or (ii) dead individuals with a whole empty shell. Large skeletal fragments retaining the apex or more than half of the hingeline were also counted as dead individuals. ~~The analysis of the diversity and distribution of live mollusks was published in Armenteros et al. (2018). Here we focus on the analysis of death mollusk assemblages.~~ Mollusks were identified to species level using taxonomic literature [40~~39-~~, 41, ~~(Abbott 1974, Mikkelsen and Bieler 2008, Tunnell et al. 2010~~42~~, Redfern 2013)~~43~~2~~].

Formatted: English (United States)

Phylogeny and biological traits

Formatted: Font: 16 pt

Phylogeny was established by the Linnean system of classification (class, order, family, genus, and species) since no other data were available for most of the species (e.g., DNA-based phylogeny). The taxonomy of the species was obtained by the Match taxa tool of the World Register of Marine Species ~~(WoRMS 2023)~~[7], ~~and~~ we used only the accepted names given at WoRMS.

We classified all the species after five biological traits: (i) life mode, (ii) feeding type, (iii) relationship with the substrate, (iv) mobility, and (v) shell attachment (S1 Table). The biological traits were mainly obtained from the database Neogene Marine Biota of Tropical America (NMITA) [443](NMITA, Todd 2023), including support literature for mollusk ecology [410, (Mikkelsen and Bieler 2008, Rosenberg et al. 2009 421, 454, , Turgeon et al. 2009465], Tunnell et al. 2010). All categories within traits were adapted following the codes given at NMITA. The biological trait information was usually given at genus or family levels, so we extrapolated at the species level if necessary.

Gastropods were classified only after their life mode and the feeding type as no other information was available at NMITA or in the consulted literature. For the Scaphopods, we were able to fill in their traits after Tunnell et al. (2010)[421].

Data analysis

We analyzed the diversity using two complementary components (Magurran 2021)[14]: richness (α- and γ-diversitiesdiversity) and β-diversity. We investigated the three dimensions of diversity: species, phylogenetic, and functional diversity. We also analyzed the evenness ecological structure of the assemblage to take ininto accountconsidering the relative abundance of the species using dominance curves, heat map and numerical ordination according to the appropriate scale. The statistical design, according to the research goalsobjectives, included two spatial scales: regional that encompassed the gulf and local that referred to the sampling site. We also included habitat in the design because it has a significant impact on the diversity in the studied region (Armenteros et al. 2018)[28]. In general, the criterion for testing differences between groups was the overlapping of the 0.95 CIs calculated on basis of permutations.

Formatted: English (United States)

Formatted: English (United States)

Formatted: Font: 16 pt

Richness

~~.~~For the species richness, we built accumulation curves versus individuals using the effective number of species (Hill's number 0) ~~(Hill 1973)~~[476]. The accumulation curves included the observed richness, the asymptotic estimates (only at regional scale), and 0.95 confidence intervals (CIs) generated by 500 permutations using the functions *inext* and *gginext* in the R package iNEXT 3.0.0 ~~(Hsieh et al. 2016)~~[487]. In the second step, we calculated the ~~observed~~ expected richness at ~~a samethe~~ the same level of abundance using rarefaction.

For the phylogenetic richness, we used the Faith's phylogenetic diversity (PD sensu ~~Faith 1992~~[498]), which is the sum of the branch lengths in a phylogenetic tree. PD measures the amount of evolutionary history across species ~~(Tucker et al. 2017)~~[15]. The phylogenetic tree was based on the Linnean hierarchy (class, order, family, genus, and species) and built with the function *linnean* in the package BAT 2.9.2 ~~(Cardoso et al. 2015)~~[4509].

For the functional richness, we used the Petchey and Gaston's functional diversity (FD sensu ~~Petchey and Gaston 2002, 2006~~[510]), which is the sum of the branch lengths in a functional tree. FD measures the overall distance between the different functional units (i.e., species) of a given assemblage ~~(Legras et al. 2018)~~[521]. Related with functional diversity, we used "response traits" that focus~~es~~ on an organism's response to its environment and does not imply that a trait ~~has necessarily~~necessarily ~~has an effect on~~influences an ecological process ~~(Streit and Bellwood 2022)~~[532]. The functional tree was built in the package BAT with the function *tree.build* using the following settings: the traits were converted from categorical to numerical (i.e., dummy variables), Gower distance as resemblance measure, and modified Neighbor-Joining algorithm as linkage strategy. Species, phylogenetic, and functional richness were calculated using the function *alpha* in

Formatted: Font: 14 pt

the R package BAT with rarefied samples (i.e., the same sample size) and using 1000 permutations for generating 0.95 CIs.

β-diversity.

Formatted: Font: 14 pt

-We followed the framework proposed by Cardoso et al. [21, (2014a, 2014b)543] that analyzes species-, phylogeny-, and functional-based β-diversity. These three dimensions of total β-diversity were in turn decomposed into two partitions (Carvalho et al. 2012, 2013)[19, 20]: replacement and richness difference. For the species-based dimension, we built β-diversity accumulation curves for evaluating the effects of sampling completeness (Cardoso et al. 2009)[554]. The pairwise values of dissimilarity were averaged over the relevant scale/level. That is, at regional scale all pairwise inter-site dissimilarities within a gulf were averaged (e.g., for GB: GB09 versus GB10, GB09 versus GB11, etc.). For habitats, pairwise inter-site dissimilarities in a habitat nested within gulf were used (e.g., for coral reef sands in GB: GB09 vs. GB11; for seagrass in GB: GB10 vs. GB12). β-diversity accumulation curves were built with the function *beta.accum* in the R package BAT. We calculated β-diversity using the function *beta* in the R package BAT. The phylogenetic and functional trees were the same as for richness analyses. The measure of dissimilarity was the Sorensen index that uses incidence data since changes in composition between samples was the target of the analysis. Calculations were made on rarefied samples (at level of smallest sample size) to reduce the effects of differences in abundance and 100 permutations were done to generate 0.95 CIs around the mean β-diversity.

Evenness.

Formatted: Font: 14 pt

-This property of the assemblage structure was represented in curves of dominance of species rank (x-axis in log scale) versus relative abundance ~~(Clarke et al. 2014)~~[565]. We also quantified the rareness based on the number of species with one individual (i.e., singletons) and two individuals (i.e., doubletons).

Taxonomic ~~Taxonomic C~~composition ~~*assemblage structure*~~.

Formatted: Font: 14 pt

In a second approach to describe the assemblage structure, ~~W~~we used the SIMPER analysis to identify those species that most contribute to the 70% of similarity (Bray-Curtis index) for a particular scale/level (i.e., gulf, habitat, or site); such species can be considered as the typical or characteristic of that scale/level ~~(Clarke et al. 2014)~~[565]. For this analysis, we averaged the abundance of species over the sites. To visualize the typical species indicated by the SIMPER, we built heat maps of their relative abundance. The typical species in the heat map were ordered on basis of their similarity across the samples using an index of association ~~(Clarke et al. 2014)~~[565]. In the case of analyses at site scale, instead of a heat map, we preferred to build a numerical ordination by non-metric multidimensional scaling (NMDS) with all the replicates to visualize the similarity pattern across sites. NMDS was built on basis of a similarity matrix calculated with the Bray-Curtis index, no transformation of data was done. A permutational analyseis of variance (PERMANOVA) [576] was made for testing ~~statistical~~ differences in the multivariate assemblage structure following ~~the~~the nested statistical design mentioned above ~~(Anderson et al. 2008)~~: GULF as main fixed factor~~, (fixed)~~, HABITAT as random factor nested within GULF, and SITE as random factor nested within ~~combinations of GULF-~~HABITAT. The settings of PERMANOVA were ~~vutur~~ Bray-Curtis as measure of resemblance, 9999 permutations for building the null distribution, ~~and~~ sum of squares type III (partial), and permutation of residuals under a reduced model.

Formatted: Font color: Auto

Formatted: Font color: Red

Functional assemblage structurecomposition.

Formatted: Font: 14 pt

-We built four matrices of trait × samples by summing species based on their biological traits: feeding type, relationship organism-substrate, mobility, and shell attachment. The trait life mode (i.e., benthic or pelagic) was not included because there were only six pelagic species. The samples were aggregated according to the scale/level of the analyses (i.e., gulf, habitat, or site). We represented the relative contribution of each trait category to the total abundance in stacked bar plots. Categories within a trait were not independent (i.e., they summed 100%), therefore no statistical tests were done to compare them. For the functional structure composition across sites, we ordered them according to their similarity based on species abundance using the Bray-Curtis index.

The relationship between distance and number of species was assessed by simple linear regression.

All graphs were made in the package Tidyverse (Wickham et al. 2019)[587] in R.

Comparison between death and living assemblages. We compared the data derived of death (present study) and living mollusk assemblages (reported in Armenteros et al. 2018) obtained from the same sampling units. Based on the raw data, we compared the abundance (number of shells 100 cm^{-2}) and species richness of both death and living assemblages for the 11 sampled sites. The idea was to evaluate the relative contribution of the death assemblages to the total abundance and species richness:

$$N\% = \frac{N_{dead}}{(N_{dead} + N_{alive})} \times 100$$

$$S\% = \frac{S_{dead}}{S_{total}} \times 100$$

Where:

Formatted: Centered

N%: Relative contribution of the death mollusk assemblage to the total abundance.

N_{dead}: Number of individuals of the dead assemblage, the replicates were summed per site.

N_{alive}: Number of individuals of the living assemblage, the replicates were summed per site.

S%: Relative contribution of the dead assemblage to the total species richness.

S_{total}: The species richness including dead and live individuals, the replicates were summed per site.

S_{dead}: The species richness only of the dead assemblage, the replicates were summed per site.

Results

Formatted: Font: 18 pt

General features

Formatted: Font: 16 pt

We identified 7113 dead dead individuals in the mollusk death assemblages: belonging to 393 species, 242 genera, 101 families, 20 orders, and three classes. Pelagic mollusks occurred only in GB and were represented by six species and 41 individuals (0.6% of total abundance). The full matrix of mollusk species × sites was given in the S1 S2 table. The most abundant species were *Parvilucina* spp. (8%), *Eulithidium* sp. (6%), *Cerithium litteratum* (4%), *Barbatia domingensis* (4%), *Eulithidium bellum* (3%), *Cerithium eburneum* (3%) and *Alvania auberiana* (3%).

Regional scale (Gulfs)

Formatted: Font: 16 pt

Richness.

Formatted: Font: 14 pt

We identified 280 and 265 mollusk species in the gulfs of Batabanó and Guanahacabibes, respectively. The two accumulation curves of species did not reach asymptotes (~~S2~~ S3 Fig). However, the sampling coverage was 0.98 for both gulfs, suggesting a high completeness of the sampling effort. At a level of rarefaction of 3000 individuals, no significant differences of species richness occurred between gulfs (Fig 2a). Nevertheless, the phylogenetic and functional richness were different between gulfs as indicated by the non-overlapped CIs (Figs 2c and 2e). In general, the Gulf of Batabanó tended to have larger richness than Guanahacabibes.

~~**Fig 2. Regional richness and mean β-diversity of mollusk death assemblages in the gulfs of Batabanó (GB) and Guanahacabibes (GG).** (a) Species richness. (b) Species β-diversity. (c) Phylogenetic richness. (d) Phylogenetic β-diversity. (e) Functional richness. (df) Functional β-diversity. In (a), (c), and (e) samples were rarefied to 3000 individuals; in (b), (d), and (f) samples were rarefied to the lowest number of individuals between pairs of sites. Two additive partitions of β-diversity are shown: replacement and richness difference. Horizontal lines = mean, boxes (or whiskers) = 0.95 confidence intervals, and vertical lines = range. Phylo. = phylogenetic, Funct. = functional.~~

Fig 2. Regional richness and mean β-diversity of mollusk death assemblages in the gulfs of Batabanó and Guanahacabibes. (a) Species richness. (b) Species β-diversity. (c) Phylogenetic richness. (d) Phylogenetic β-diversity. (e) Functional richness. (f) Functional β-diversity. Samples were rarefied to 3000 individuals iIn (a), (c), and (e) ~~samples were rarefied to 3000 individuals;~~ while in (b), (d), and (f) were rarefied to the lowest number of individuals between pairs of sites. Two additive partitions of β-diversity are shown: replacement and richness difference. Horizontal lines = mean, boxes (or whiskers) = 0.95 confidence intervals, and vertical lines = range. Phylo. = phylogenetic, Funct. = functional

Formatted: English (United States)

Formatted: English (United States)

Formatted: English (United States)

Formatted: English (United States)

Formatted: English (United States)

Formatted: English (United States)

Formatted: Space After: 0 pt, Line spacing: Double

Formatted: English (United States)

Formatted: English (United States)

β-diversity

Formatted: Font: 14 pt

~~.~~ The accumulation curves suggested a modest decrease of mean β-diversity with the increase of the replication suggesting that the current sample size (n = 3, for most of the sites) is enough for a robust comparison (~~S2~~ S3 Fig). The total species β-diversity was high (> 0.6) and similar for GB and GG (Fig 2b). The replacement partition was higher than richness difference partition in both gulfs. Phylogenetic β-diversity was similar between the gulfs (around 0.5), and with replacement partition being higher than richness difference as well (Fig 2d). Functional β-diversity had the same pattern, although total values of β-diversity were lower (< 0.5) (Fig 2f).

Evenness~~.~~

Formatted: Font: 14 pt

The evenness was similar in the two gulfs as indicated by the similar shape of the dominance curves (Fig 3a). The main difference between the two curves is the level of dominance of the most abundant species, which was 17% and 7% in GG and GB, respectively. About nine species contributed less than 5% each to the total abundance in any gulf followed by a long tail of rare species represented by singletons (71 in GB and 77 in GG) and doubletons (38 in GB and 48 in GG).

Fig 3. **~~Assemblage structure~~Evenness of mollusk death assemblages ~~represented by Ddominance curves.~~** ~~represented by d~~Dominance curves of ~~of r~~Rrelative contribution of species to the total abundance ~~Regional e~~Evenness ~~and taxonomic assemblage structure~~versus abundance ~~order~~ species rank ~~of mollusk death assemblages.~~ **~~in the gulfs of Batabanó (GB) and~~**

Formatted: Font: Bold

Formatted: Font: Bold

Formatted: Font: Bold

Formatted: Font: Not Bold

Formatted: Font: Not Bold

~~**Guanahacabibes (GG).**~~ (a) Regional scale. ~~Regional Dominance curves of relative contribution of species to the total abundance.~~ (b) Habitat. (c) Site scale~~.~~

~~Heat map of relative abundance (in %) of those typical species that contribute to the 70% of similarity within each gulf. Note that species were ordered by their similarity across the gulfs.~~

Formatted: Font color: Red

Taxonomic ~~assemblage structure~~composition~~.~~

Formatted: Font: 14 pt

The heat map representing the assemblage structure of 25 typical species for the gulfs suggested that they were largely the same, despite variability~~fluctuations~~ in the relative abundances (~~Fig 3b~~S4 Fig). In other words, of these 25 typical species, only two species (*Smaragdia viridis* and *Ervilia nitens*) occurred in a single gulf (GB), the other 23 species occurred with variable importance in both gulfs.

Functional ~~assemblage structure~~composition~~.~~

Formatted: Font: 14 pt

The trait feeding type was represented by five dominant categories, the category "others" lumped together to: deposit feeders, herbivores/suspensivores, and herbivores/carnivores. Herbivores dominated the functional trophic structure (Fig 4a). The most striking difference between gulfs was the larger abundance of chemosynthetic deposit feeders in GG. The trait relationship organism-substrate included three dominant categories, and the category "others" lumped together to: nestler, borer, and the mixed categories epifaunal/semi-infaunal/borer. The dominant category was infauna, followed by epifauna, and facultative epifauna/infauna (Fig 4b). The two gulfs had a similar contribution of each category to the total abundance. The trait mobility was represented by two dominant categories, and the category "others" lumped together to: swimming, immobile, and the

mixed categories actively mobile/sedentary/immobile. The actively mobile forms strongly dominated in the two gulfs in terms of abundance, followed by sedentary forms (Fig 4c). The trait shell attachment included two dominant categories and the category “others” lumped together to: cemented and mixed categories unattached/bysally attached. The unattached forms were dominant in both gulfs, however the bysally attached forms were more important in GB when compared with GG (Fig 4d).

Fig 4. Regional functional ~~assemblage structure~~composition of mollusk death assemblages in the gulfs of Batabanó (GB) and Guanahacabibes (GG). (a) Feeding types, including carnivore, chemosymbiotic deposit feeder, herbivore/deposit feeder (facultative), herbivore, suspension feeder, and others. (b) Relationships organism – substrate, including infauna, epifauna, epifauna/infauna (facultative), and others. (c) Mobility, including actively mobile, sedentary, and others. (d) Shell attachment, including unattached, bysally attached, and others. The traits in (b), (c), and (d) were calculated only for bivalves. See the text for details about “~~O~~others”~~**Fig 4. Regional functional assemblage structure composition of mollusk death assemblages in the gulfs of Batabanó (GB) and Guanahacabibes (GG)**. (a) Feeding types, including carnivore, chemosymbiotic deposit feeder, herbivore/deposit feeder (facultative), herbivore, suspension feeder, and others. (b) Relationships organism – substrate, including infauna, epifauna, epifauna/infauna (facultative), and others. (c) Mobility, including actively mobile, sedentary, and other. (d) Shell attachment, including unattached, bysally attached, and others. The traits in (b), (c), and (d) were calculated only for bivalves. See text for details about the categories “others”.~~

Habitat

Formatted: English (United States)

Formatted: English (United States)

Formatted: English (United States)

Formatted: English (United States)

Formatted: English (United States)

Formatted: Font: 16 pt

Richness~~.~~

Formatted: Font: 14 pt

~~-~~We used the nested structure to ~~compare between~~compare two habitat types within the corresponding gulfs. The species accumulation curves were not asymptotic (~~S2~~ S3 Fig). However, the sampling coverage was high as well, ranging between 0.95 and 0.98. ~~Coral R~~Reef sands~~s~~ had higher mollusk species richness than seagrass; and within seagrass meadows, gulf of Guanahacabibes had higher richness than Batabanó (Fig 5a). Phylogenetic richness had the same pattern, with higher richness in~~of~~ the ~~coral~~ reef sands, intermediate in seagrass from Guanahacabibes, and lowest in seagrass from Batabanó (Fig 5c). Functional diversity showed a different pattern, with higher richness in ~~coral~~ reef sands~~s~~ from Batabanó, and lower in the other three groups (Fig 5e).

Fig 5. Habitat richness and mean β-diversity of mollusk death assemblages in ~~coral~~ reef sands (~~C~~RS) and seagrass (S) within the gulfs of Batabanó (GB) and Guanahacabibes (GG). (a) Species richness. (b) Species β-diversity. (c) Phylogenetic richness. (d) Phylogenetic β-diversity. (e) Functional richness. (f~~d~~) Functional β-diversity. Samples were rarefied to 1000 individuals i~~I~~n (a), (c), and (e) ~~samples were rarefied to 1000 individuals~~; while in (b), (d), and (f) ~~samples~~ were rarefied to the lowest number of individuals between pairs of sites. Two additive partitions of β-diversity are shown: replacement and richness difference. Horizontal lines = mean, boxes (or whiskers) = 0.95 confidence intervals, and vertical lines = range. Phylo. = phylogenetic, Funct. = functional~~.~~

β-diversity~~.~~

Formatted: Font: 14 pt

-β-diversity accumulation curves showed a moderate decrease from 1 to 3 sampling units (~~S2~~ S3 Fig) indicating that current sample size was enough for a robust comparison. There were no differences of species β-diversity between ~~coral~~ reef sands and seagrass in the Gulf of Batabanó. However, in the Gulf of Guanahacabibes the species β-diversity was slightly higher in seagrass compared with ~~coral~~ reef sands (Fig 5b). The replacement partition was much more important than richness differences for all the combinations habitat-gulf. The phylogenetic β-diversity was similar between the two habitats nested within gulfs, and the replacement partition was also the larger (Fig 5d). The functional β-diversity did not show differences between habitats nested within gulfs (Fig 5f). But the richness differentiation partition increased relatively (around 50% of total β-diversity) suggesting that loss/gain of trait categories played a more important role than simple replacement of one category by another.

Evenness~~.~~

Formatted: Font: 14 pt

-There were differences among the habitats nested within gulfs as indicated by the four curves (Fig ~~6a~~3b). However, seagrass had larger dominance compared to~~with~~ ~~coral~~ reef sands, given by the two most abundant species (23% and 15% in GG and GB, respectively). The 2nd and 3rd most abundant species for both types of habitats contributed between 5% and 12% of the total, suggesting a rather high evenness. As expected, there was a long tail of rare species represented by an average (over the four combinations) of 52 singletons and 31 doubletons.

~~Fig 6.~~ Assemblage structure ~~Habitat evenness and t~~Taxonomic ~~assemblage structure~~composition ~~of mollusk death assemblages in coral~~ reef sands ~~(CRS)~~

Formatted: Font: 14 pt

~~and seagrass (S) within the gulfs of Batabanó (GB) and Guanahacabibes (GG)~~. ~~(a) Dominance curves of relative contribution of species to the total abundance. (b) Heat map of relative abundance (in %) of those typical species that contribute to the 70% of similarity within each habitat. Note that species were ordered by their similarity across the habitat-gulf combinations.~~

Taxonomic ~~assemblage structure~~composition~~.~~

The heat map of typical species suggested sharp differences between habitats. In GB, there were 14 species typical of ~~coral~~ reef sands but absent in seagrass, and conversely three species typical of seagrass but absent in ~~coral~~ reef sands (Fig 6~~b~~). By other side, in GG, there were eight species typical of ~~coral~~ reef sands but absent in seagrass, and four species typical of seagrass but absent in ~~coral~~ reef sands.

Fig 6. Assemblage structure of mollusk death assemblages in reef sands (RS) and seagrass (S) within the gulfs of Batabanó (GB) and Guanahacabibes (GG). Heat map of relative abundance (in %) of those typical species that contribute to the 70% of similarity within each habitat. Note that species were ordered by their similarity across the habitat-gulf combinations

Functional ~~assemblage structure~~composition~~.~~

Formatted: Font: 14 pt

Feeding type structure had notable differences between habitats (Fig 7a). The feeding type structure in ~~coral~~ reef sands from the two gulfs was similar, with dominance of herbivores, followed by

carnivores and suspension feeders. Feeding type structure in seagrass was different compared to ~~coral~~ reef sands, but also had differences between GB and GG. For instance, in GB suspension feeders was the most important category, while in GG was chemosynthetic deposit feeders. The relationship organism-substrate was quite different between habitats nested within gulfs (Fig 7b). In ~~coral~~ reef sands, epifauna represented more than half of the abundance, while in seagrass they were rather scarce. Seagrass, in both gulfs, were largely dominated by infauna. Mobility had a similar pattern with sharp differences between habitats (Fig~~.~~ 7c). In ~~coral~~ reef sands, sedentary forms were similarly abundant to actively mobile forms. However, in seagrass the sedentary forms virtually disappeared, and only actively mobile forms occurred. Shell attachment had a similar pattern to the other two previous traits. There were notably differences between habitats nested within gulfs (Fig 7d). In ~~coral~~ reef sands, bysally attached and unattached forms were roughly equally important, and the category other occurred in much lesser proportion. While in seagrass, unattached forms largely dominated the assemblages.

Fig 7. Habitat functional ~~assemblage structure~~composition of mollusk death assemblages in ~~coral~~ reef sands (~~C~~RS) and seagrass (S) within the gulfs of Batabanó (GB) and Guanahacabibes (GG). (a) Feeding types including carnivore, chemosymbiotic deposit feeder, herbivore/deposit feeder (facultative), herbivore, suspension feeder, and others. (b) Relationships organism - substrate including infauna, epifauna, epifauna/infauna (facultative), and others. (c) Mobility, including actively mobile, sedentary, and others. (d) Shell attachment, including unattached, bysally attached, and others. The traits in (b), (c), and (d) are ~~were calculated~~ only for bivalves. See the text for details about “~~the categoryies o~~Oothers”~~.~~

Local scale (sites)

Richness~~.~~

-The shapes of the species accumulation curves for sites were non-asymptotic (~~S2~~ S3 Fig). However, the sampling coverage was relatively high and ranged from 0.86 at GG02 (with single replicate) to 0.97 at GB09 and GB10. The most striking point in the curves was the large abundance of dead shells in the ~~coral~~ reef sand sites GB09 and GB11. The species richness varied significantly across sites with the most obvious differences between ~~coral~~ reef sand sites (GB09, GB11, GG01, GG02, and GG04) with higher richness and seagrass sites with lower richness (Fig 8a). The phylogenetic richness showed a similar pattern with high variability across sites, and significant differences between ~~coral~~ reef sand and seagrass sites (Fig 8c). Functional richness did not show differences across sites due to the overlapping of the 0.95 CIs (Fig 8e).

Fig 8. Local richness and mean β-diversity of mollusk death assemblages in 11 sites within the gulfs of Batabanó (GB) and Guanahacabibes (GG). (a) Species richness. (b) Species β-diversity. (c) Phylogenetic richness. (d) Phylogenetic β-diversity. (e) Functional richness. (~~d~~f) Functional β-diversity. Samples were rarefied to 300 individuals i~~I~~n (a), (c), and ~~(e)(e)€ samples were rarefied to 300 individuals~~; while in (b), (d), and (f) ~~samples~~ were rarefied to the lowest number of individuals between pairs of sites. Two additive partitions of β-diversity are shown: replacement and richness difference. Horizontal lines = mean, boxes (or whiskers) = 0.95 confidence intervals, and vertical lines = range. Phylo. = phylogenetic, Funct. = functional

β-diversity~~.~~

Formatted: Font: 16 pt

Formatted: Font: 14 pt

Formatted: English (United States)

Formatted: Font: 14 pt

-Species β-diversity was around 0.5, with no differences among sites as suggested by the overlapping of the 0.95 CIs. The most important contribution to the total β-diversity was given by species replacement (Fig 8b). Phylogenetic β-diversity was in general lower than 0.5, but again without clear differences among sites. Most of the phylogenetic β-diversity was given by species replacement (Fig 8d). Functional β-diversity had a rather similar pattern, without differences among sites, but a larger relative contribution of richness differences to the total β-diversity (Fig 8f).

Evenness

-The evenness was similar for the 11 sites based on the dominance curves (Fig 9a3c). However, two seagrass sites in Guanahacabibes had higher dominance of a single species (GG06 with 41% and GG09 with 37%). A long tail of rare species was represented by a mean number of singletons over the 11 sites of 37 species (range: 19–68 species) and a mean number of doubletons of 18 species (range: 4–38 species).

Fig 9. Assemblage structure Similarity patterns based on Local evenness and tTtaxonomic assemblage structurecomposition of mollusk death assemblages in 11 sites within the gulfs of Batabanó (GB) and Guanahacabibes (GG) based on taxonomic composition). Similarity patterns in (a) Dominance curves of relative contribution of species to the total abundance. (b) NMDS ordination plot of 29 samples from 11 sites based on species abundance. Samples were coded by habitat (color and shape) and gulf (shapeopen/close symbol).

Formatted: Font: 14 pt

Formatted: Font: 14 pt

Formatted: Font: 14 pt, Not Bold

Formatted: Font: 14 pt

Taxonomic ~~assemblage structure~~composition~~.~~

~~-~~For ~~visualizinganalizing~~analyzing changes in taxonomic composition at ~~At~~ site scale, we searched for the similarity patterns across sites. ~~selected to buil preferred to show the The~~A NMDS ordination plot of the 29 samples (= points) based on species abundance indicated a clear pattern ~~of structure~~ based on the habitat type (Fig 9~~b~~). ~~Coral r~~Reef sands and seagrass sites clustered apart from each other ~~together in the upper half of the~~ in the plot and showed significant differences (PERMANOVA, S5 table). However, ~~; but also, there were small~~ differences in assemblage structure between gulfs were less marked in the plot and non-significant after PERMANOVA.~~, with GB samples in the upper left and GG samples in the upper right. In a similar way, sSeagrass sites also clustered in the lower half together although with higher dispersal of the points. These samples were also slightly separated after gulfs, with GG samples in the lower left and GB samples in the lower right.~~ The variability between replicates from the same site was also considerable and statistically significant (PERMANOVA, S5 table)~~y~~ suggesting high variability in the assemblage structure. Indeed, ~~The , (PERMANOVA).F~~factors HABITAT and SITE explained about one third each of the total variance, meanwhile remaining third was unexplained (= residual).~~s d sites~~,

Fig 9. Assemblage structure of mollusk death assemblages within the gulfs of Batabanó (GB) and Guanahacabibes (GG). Similarity patterns in a NMDS ordination plot of 29 samples from 11 sites based on species abundance. Samples were coded by habitat (color and shape) and gulf (open/close symbol)

Formatted: Font: Not Bold

Formatted: Font color: Auto

Formatted: Font color: Red

Functional ~~assemblage structure~~composition

Formatted: Font: 14 pt

~~.~~We ordered the sites along the x-axis based on their similarity using the species abundance. The feeding type structure showed differences related with the habitats again (Fig 10a). Sites within the ~~coral~~ reef sands (GG09, GB11, GG01, GG04, and GG02) had herbivores as the most important category, followed by carnivores. Heterogeneity in feeding type composition was larger in seagrass; some sites (GB12, GG05, GG09, and GG06) had an important contribution of chemosynthetic deposit feeders, while at GG14 dominated herbivores and at GB10 dominated suspension feeders. The relationship organism-substrate had large heterogeneity in ~~coral~~ reef sand sites (Fig 10b). Three ~~coral~~ reef sand sites (GB11, GG01, and GG04) had dominance of epifaunal forms, while at GG09 dominated infauna, and in GG02 facultative epifauna/infauna. In seagrass sites, infaunal forms were dominant, although epifauna was important at GG09. The mobility had a rather regular pattern also associated to the habitat (Fig 10c). Three ~~coral~~ reef sand sites (GB11, GG01, and GG04) had dominance of sedentary forms, while seagrass sites (GB10, GB12, GG14, GG05, GG09, and GG06) and two ~~coral~~ reef sand sites (GG02 and GB09) were largely dominated by actively mobile forms. The shell attachment had a similar pattern of habitat influence (Fig 10d). Three ~~coral~~ reef sand sites (GB11, GG01, and GG04) had dominance of bysally attached forms. The other two ~~coral~~ reef sand sites (GG02 and GB09) had important contributions of bysally attached forms, but the dominance was given by unattached forms. The latter category was largely the most important in seagrass sites.

Fig 10. Local functional ~~assemblage structure~~composition of mollusk death assemblages in 11 sites within the gulfs of Batabanó (GB) and Guanahacabibes (GG). (a) Feeding types including carnivore, chemosymbiotic deposit feeder, herbivore/deposit feeder (facultative), herbivore, suspension feeder, and others. (b) Relationships organism - substrate including infauna, epifauna,

epifauna/infauna (facultative), and others. (c) Mobility includes actively mobile, sedentary, and others. (d) Shell attachment including unattached, bysally attached, and others. The traits (b), (c), and (d) were calculatedare only for bivalves. See the text for details about the categoriesy "oOothers". Note that the sites were ordered by their similarity based on species abundance.

Contribution of the dead mollusks to the total assemblage

In terms of abundance, the dead mollusks contributed highly (range: 95%–100%) to the total number of shells in the assemblages (Table 2). In terms of species richness, the dead mollusks also contributed in very high proportion (91%–100%) to the total species richness of the assemblages (Table 2). In most of the sites (except GB10 and GG02), there were a few species in the living assemblage that were not recorded in the dead assemblage as indicated by the contribution lower than 100% of death assemblages. *Parvilucina* spp. contributed more than 40% of total abundance in live assemblages at seagrass sites GG05, GG06, and GG09. These species were also important in the death assemblages at the same sites (16%, 41%, and 37%, respectively).

Table 2. Contribution of the mollusk death assemblages to the total abundance (N%) and species richness (S%).

Data of living assemblages are found infrom Armenteros et al. (2018). The replicates were summed per site. Abbreviations: N%= Relative contribution of the death mollusk assemblage to the total abundance, N_{dead}= Number of individuals of the dead assemblage, N_{alive}= Number of individuals of the living assemblage, S%= Relative contribution of the dead assemblage to the total species richness, S_{total}= The species richness including dead and live individuals, S_{dead}= The species richness only of the dead assemblage, S_{alive}= The species richness only of the living assemblage.

Formatted: Space Before: 0 pt

Shelf benthic species at deep-sea site SL-38-750 in the Gulf of Guanahacabibes

There were 14 mollusk species from shallow waters in the deep sea off the Gulf of Guanahacabibes (site SL-38-750); thirteen species were gastropods and only one bivalve. Eleven of these shelf benthic species were also found in the mollusk death assemblages within the shallow sites of the Gulf of Guanahacabibes, and only *Granulina* sp., *Mytilopsis leucophaeata* and *Rissoella* sp. were absent (Table 3). Most of the shelf benthic species present in the deep sea were rather rare in the shallow sites (< 5% of total abundance); except for *Cerithium litteratum* (18% of total abundance). The number of benthic shelf species found in the deep sea decreased linearly and significantly with the distance (km) between the site SL-38-750 and the seven shallow sites of this study (Fig 11).

Table 3. Dead mollusk species from shallow waters reported at deep sea.

Species	Class	GG01	GG02	GG04	GG05	GG06	GG09	GG14
Alaba incerta	G	0.3	-	-	0.1	-	-	-
Alvania auberiana	G	-	1	0.8	2	1	4	0.5
Bittiolum varium	G	-	-	-	-	0.2	-	-
Caecum regulare	G	-	3	0.3	-	-	-	-
Cerithiopsis spp.	G	0.3	0.7	0.5	-	0.7	0.4	1
Cerithium atratum	G	-	-	0.6	-	-	-	-
Cerithium litteratum	G	18	9	9	-	-	0.2	0.8
Eoacmaea pustulata	G	7	-	0.8	3	1	-	-
Eulithidium bellum	G	1	10	2	4	-	-	11
Finella adamsi	G	-	-	0.3	-	2.3	-	0.8
Granulina sp.	G	-	-	-	-	-	-	-
Ividia havanensis	G	-	0.7	-	-	-	-	-
Mytilopsis leucophaeata	B	-	-	-	-	-	-	-
Rissoella sp.	G	-	-	-	-	-	-	-

Relative abundance (%) of mollusk species at the seven studied sites in the Gulf of Guanahacabibes. Species absent in the shallow

sites are in bold. These species were reported by Peraza-Escarrá et al. (2022) at site SL-38-750 (1670 m depth). Abbreviation: B = Bivalvia, G = Gastropoda.

Formatted: Font: 18 pt, English (United States)

Formatted: Font: 18 pt

Fig 11. Number of transported shelf benthic species vs. distance (km) from each site within the gulf of Guanahacabibes to the deep-sea site SL-38-750. The text inside the plot shows the equation of linear regression, the p-value for the hypothesis test $\beta \neq 0$, and the coefficient of determination (R^2).

Discussion

Our data provide a synoptic picture of the mollusk death assemblages in two habitats within two shallow gulfs: Batabanó (GB) in the Caribbean Sea and Guanahacabibes (GG) in the Gulf of Mexico. We detected ecological patterns related to the scale and habitat type. Our data are robust despite of potential taphonomic biases (see below) of death assemblages. We identified 7113 shells belonging to 393 species, that can be considered as a large sample size. However, the curves of species accumulation, particularly for habitats and at site scale, were hardly asymptotic suggesting that hyper-diverse assemblages may never reach an asymptote (Gotelli and Colwell 2001)[598].

Estimates of sampling coverage were in general acceptable (i.e., > 90%) suggesting a fair description of the diversity and ~~community~~ assemblage structure of mollusk death assemblages.

~~We organized this section around the three objectives. As the first objective encompasses distinctive processes at different scales/habitats, we split it after regional scale, habitat-related, and local scale.~~

Mollusk death assemblages at regional scale

Formatted: Font: 16 pt

The two gulfs showed coherent similarity in terms of species richness, β-diversity, evenness, taxonomic and functional assemblage structures (except for the feeding type); also, PERMANOVA ~~suggested~~indicated no statistical differences ~~of~~in ~~multivariate~~the assemblage structure. Similar geological and geographical settings in the western region of Cuba Island may account for these commonalities between Batabanó ~~GB~~ and Guanahacabibes~~G~~. For instance, β-diversity, assemblage composition and functional structure may be explained by three general factors after ~~van der Plas et al. (2023)~~[~~60~~59]. First, abiotic heterogeneity, which was similar between the two gulfs given the occurrence of typical tropical habitats in both basins (e.g., mangroves, muddy bottoms, seagrass meadows, and coral reefs). The abiotic data of water column and sediments, although with similar average values between gulfs, varied notably within gulfs evidencing the environmental heterogeneity. Second, the regional pool of species should be similar in both gulfs given they belong to the same biogeographic realm (i.e., tropical western Atlantic, ~~Costello et al. 2017~~[6~~1~~0]). Third, the dispersal rates, which ~~is~~are related to species' biology and on the oceanographic regime. However, it wa~~i~~s ~~difficult to~~not possible to compare both gulfs in terms of the oceanographic regime because of the lack of ~~published~~available ~~with the available~~ data.

Formatted: English (United States)

Formatted: English (United States)

There were two main differences between the ~~two~~ gulfs: richness (phylogenetic and functional) and feeding type composition. First, phylogenetic and functional richness were higher in ~~GB~~ Batabanó

maybe because it has a more advantageous balance of productivity-disturbance. According to ~~Huston (1994)~~[6~~2~~1], richness depends on the ecological time of the regimes of productivity and disturbance. The eastern portion of the Gulf of Batabanó, where the four sites were located (Fig 1), is an area of high biological productivity due to the confluence of freshwater (and nutrients) runoff from Zapata Swamp and the influence of the Caribbean current on the shelf ~~(Lluis-Riera 1972)~~[6~~3~~2]. Local upwellings, eddies and tidal currents have been reported to promote the exchange between the open ocean and the southeastern portion of ~~GB~~ Gulf of Batabanó ~~(Arriaza et al. 2008)~~[6~~4~~3]. Additional evidence of the large productivity of eastern ~~G~~Batabanó is the healthy coral reefs status with coral colonies of large size ~~(Caballero Arag~~~~ó~~~~on et al. 2019)~~[31] and the high finfish and spiny lobster catches ~~(Baisre et al. 2018)~~[6~~5~~4]. Besides, the disturbance regime seems to be high in the shelf border of eastern ~~GB~~Batabanó. High contribution of bysally attached bivalves ~~in GB~~, adapted to strong currents, and the occurrence of skeletal remnants of oceanic pelagic species suggest stronger influence of open ocean. In addition, ~~coral~~ reef sands in ~~GB~~ Batabanó exhibited the largest number of shells (and total species richness) suggesting high secondary productivity (S2 Fig).

The feeding type composition was different between ~~GG~~ Batabanó and ~~GB~~Guanahacabibes, with higher importance of chemosynthetic bivalves (Lucinids) in ~~GG~~the latter. These bivalves dominated in sulfide-rich hypoxic sediments ~~(Altieri et al. 2021)~~[6~~6~~5], like the four seagrass sites located in the inner part of ~~GG~~Guanahacabibes. On contrary, the seagrass sites in ~~GB~~ Batabanó did not show symptoms of hypoxia (personal observation) likely because oxygen replenishment due to intense hydrodynamics. The different functional composition was also reflected in the evenness between gulfs: In ~~GG~~ Guanahacabibes the dominance was higher than in ~~GB~~ Batabanó due to the occurrence of *Parvilucina* spp. (17%).

Formatted: English (United States)

Two additional regional patterns were related to β-diversity. ~~First~~The first pattern was derived from the comparison of β-diversity dimensions (i.e., species versus phylogenetic versus functional). The species β-diversity was the highest (about 0.6) because of the high number of rare species that promote the differences between sites. By other side, functional β-diversity was the lowest (about 0.3) likely reflecting that taxonomically different species were replaced by functionally similar trait combinations, which are the ones that evolved adaptations to the benthic environments in ~~coral~~ reef sands and seagrass. Similar findings were reported by ~~Virta (2023)~~[676] for diatom assemblages. Phylogenetic β-diversity was intermediate (about 0.5) because composition at higher taxonomic level (i.e., genus, family, etc.) reflected a~~n~~ balance between species rareness and similar functional trait combinations.

A second regional pattern of β-diversity, for the three diversity dimensions, was the larger contribution of the replacement partition compared to the richness partition. ~~Legendre (2014)~~[18] stated that replacement is due to ecological tolerance or niche breadth, while richness is due to the number of niches. Our findings broadly agree with latter statements because many species with potentially different niche breadth occurred in ~~GB and GG~~both gulfs. While there was a similar number of available niches in the gulfs because they share geological and geographical features; for instance, same habitats (e.g., mangroves, seagrass meadows, and coral reefs) and same engineer species (e.g., *Thalassia testudinum* in seagrass and *Orbicella faveolata* in forereefs).

We recorded high evenness of the assemblages at regional scale, with the presence of one moderately abundant species and a very long tail of rare species. This pattern agrees with the very high richness and low dominance of mollusk coastal assemblages in Cuban archipelago as reported by other authors [33-1, 34, 365, 387]~~, Hoskins (1964), Capetillo-Piñar et al. (2015), and Armenteros et al. (2012, 2021)~~. The high evenness reported in our study is also enhanced by two features ~~(Kidwell and Bosence 1991)~~[5]: (1) time averaging increases the probability of several shifts in

Formatted: English (United States)

species dominance through time; although we have evidence of limited time averaging in our study (see below). And (2), at regional scale there is more tendency to reflect the share dominance of multiple species within a window of time, resulting in lower dominance.

Mollusk death assemblages: ~~coral r~~Reef sands versus seagrass meadows

Formatted: Font: 16 pt

Habitat type had a strong influence on all the metrics of assemblages: Richness, β-diversity, evenness, taxonomic and functional assemblage structures~~.~~; ~~also~~ ~~Mmultivariate assemblage structure~~PERMANOVA also indicated a statistically significant effect of the habitat type. This supports the strong role of habitat type on the diversity and assemblage structure of dead [10, ~~(Weber and Zuschin 2013, Zuschin and Ebner 2015~~687, ~~Bracchi et al. 2023)~~ 698] and live mollusks ~~(Armenteros et al. 2018)~~[28].

Coral reefs harbor a high richness of mollusks in their different substrates [~~70~~69]~~(Barrientos-Luján et al. 2021 and references therein)~~. The relationship between productivity and disturbance may also explain this pattern ~~(Huston 1994)~~[6~~2~~1]~~-~~ since coral reefs receive high quality organic carbon produced by a diverse group of primary producers that includes microphytobenthos, macroalgae, zooxanthella and phytoplankton ~~(Côté and Knowlton 2013)~~[29]. At the same time, disturbance is strongly related to physical forces from waves and currents, so species may require adaptation against resuspension (e.g., bisally attached bivalves) but promotes oxygen replenishment avoiding hypoxia and generation of sulfur hydrogen in sediments. As opposite, although seagrass harbor a large biodiversity, the balance of productivity-disturbance is likely ~~-~~less favorable. For instance, the

fraction of primary production of seagrass is highly refractory due to the content of lignin, so the most labile fraction (e.g., derived from epiphytic microalgae) is that is mostly used by grazer mollusks, mainly gastropods (Hemminga and Duarte 2000)[710]. Seagrass canopy reduces the physical disturbance and associated resuspension risk of benthic organisms, but at the same time reduces oxygen replenishment causing hypoxia and generation of hydrogen sulfide (Altieri et al. 2021)[665].

Each habitat had a distinctive assemblage structure suggesting that the identity can be preserved in the dead assemblages long after death (Casebolt and Kowalewski 2018)[4]. It is explained by differences in richness (see above) and adaptative traits (see below), but also for a rather limited shell transport between adjacent habitats. The transport of dead shells is an important postmortem process [5, (Kidwell and Bosence 1991, Kidwell 2008)721], ; but likely the physical structure of the habitats in our study canmay reduce it. Seagrass meadows are physically protected environments because canopy and rhizomes reduce the erosion by currents and waves (Hemminga and Duarte 2000)[710], meanwhile spur-and-grooved structures in coral reefs are also physically sheltered by rocky reef structures (Davis et al. 2021)[732]. Albano and Sabelli (2011)[743] reported similar findings about reduced lateral shell transport in seagrass meadows and coral reefs in a study on fidelity of live and death assemblages. These authors explained that the reduced out-of-habitat transport is due byto soft-sediment patches surrounded by corals in coral reefs and by dense leave/rhizome mats in seagrass meadows.

The functional structure changed between the two habitat types reflecting niche preferences. Coral rReef sands were characterized by mollusk assemblages dominated by epifaunal herbivores adapted to graze on the microphytobenthos,; in particular, grazer gastropods represented a major contributed importantly ors to this guild. In sandy spots, microphytobenthos has a large primary productivity and provides high food availability to the benthic consumers [754, (MacIntyre et al. 1996, Miller et

~~al. 1996~~7~~6~~5~~)~~]. Interestingly, bivalve suspension feeders were not dominant in ~~coral~~ reef sands, likely reflecting low availability of suspended particles in the sandy spots. Sedentary species, mostly bisally attached bivalves, also thrived in ~~coral~~ reef sands reflecting the adaptation to the intense hydrodynamic, specifically to the resuspension by currents. In seagrass meadows, chemosynthetic detritivores were more important, suggesting that much of the primary production done in the seagrass canopy reach the bottom as detritus. Seagrass from ~~GB~~ Batabanó and Guanahacabibes~~G~~ differed in the importance of suspension feeders and chemosynthetic bivalves reflecting the influence of hydrodynamic regime on the functional structure. Suspension feeders dominated ~~in GB~~ Batabanó's seagrasses because of intense hydrodynamic regime in the border of the shelf. Current velocity exerts a physical disturbance on grazers, although it is counteracted by the shoot density of the seagrass meadow ~~(Jiménez-Ramos et al. 2019)~~[7~~7~~6]. In ~~GB~~ Batabanó's seagrass, the high shoot density reported by us (Table 1) possibly enhanced the protection against resuspension promoting the dominance of unattached forms. Meanwhile chemosynthetic lucinid bivalves were more important in ~~G~~Guanahacabibes because slower hydrodynamics reduce oxygen replenishment and promote hydrogen sulfide generation in sediments ~~(Altieri et al. 2021)~~ [6~~6~~5]. ~~In addition, i~~Infaunal, actively mobile and unattached forms dominated in all seagrass meadows probably as an adaptation to look for more oxygenated spots from reduced sediments and/or foraging for less refractory detritus.

Mollusk death assemblages at local scale

Formatted: Font: 16 pt

Local species richness reflected the combined influence of the regional pool of species ~~(Witman et al. 2004)~~[787] and habitat type ~~(Armenteros et al. 2018)~~ [28]. In our study, ~~fluctuations~~ variability of species richness from site to site, within same gulf and habitat type, responded to the local

environment. For instance, Tthe highest richness and abundance in GB11 and GG04 responded to local environment conditions that enhanced deposition of shells in sandy spots within spur-and-groove structures.

The total β-diversity at local scale showed moderate shifts from site to site mostly because of differences in abiotic heterogeneity (van der Plas et al. 2023)[6059]. In terms of β-diversity partitions, many different species with potentially different niche breadth may explain the higher replacement partition of β-diversity across sites (Legendre 2014)[18]. Conversely, similar number of niches across sites within the same habitat explains the lower contribution of richness partition. These findings and explanations were based on theoretical grounds about the relationship between the two β-diversity partitions reviewed by Legendre (2014).

Evenness curves indicated two sites subjected to disturbance where few species of lucinids tolerant to deoxygenation were dominant: GG06 and GG09. Historical trends of richness and abundance in sites GG06 and GG09, based on core stratigraphy and ^{210}Pb dating, indicated significant decline of mollusk diversity as result of sedimentation and eutrophication (Armenteros et al. 2021)[387]. The pattern of similarity among replicates, represented in the NMDS, strongly suggests the overarching effect of habitat type on the assemblage structure, which agrees with other studies (e.g., Barrientos-Lujan et al. 2021[7069]). Nevertheless, substantial variability among replicates within a site occurred probably as response of assemblages to small-scale sediment heterogeneity [798-, , which has been reported as well by (Hewitt et al. (2005), Creed and Kinupp (201180), 810]and Davis et al. (2017)). Indeed, SITE explains about one third of the total variance inas indicated by the multivariate assemblage structurePERMANOVA.

Effects of time averaging ofn the diversity outrcomesoutcomes

Formatted: English (United States)
Formatted: English (United States)
Formatted: English (United States)
Formatted: English (United States)
Formatted: Font: 16 pt

The process of time averaging explains the larger richness inof the mollusk death assemblages in our study (Kidwell and Bosence 1991)[5]. . and, iInstead of a bias, the time averagving can be considered as may be analyzed as a scaling post-mortem process that averages the input of shells over time (Kidwell and Tomašových 2013)[2]., Doesso Thus, we can expect that oura death assemblage study may informs more about biodiversityprovidesed -thanmaking thea typicalsingle snap-shot study of a single biological survey ofusing onlyabout the living assemblagemolluskscommunity and time and the affecting the temporal resolution (Kidwell and Tomašových 2013). as a , but also adds a source of uncertainty about the temporal matching between death and live assemblages. The extension of the time averaging depends on some non-exclusive processes such as (Kidwell 2013)[1]: (i) the intrinsic durability of the remains, (ii) the extent of the physical and biological reworking processes that can bury or exhume the remains within the mixed layer of sediments, and (iii) the sedimentation rates.

An accurate estimation of the stratigraphic resolution and time averaging in the Holocene-Anthropocene record ultimately requires that age models are coupled with post-mortem age-frequency distributions of shells dated by amino-acid racemization, ^{14}C or U-Th (Tomašových et al. 2023)[3]. Nevertheless, latter authors also stated that simple age models for cores thus still can be informative about the temporal scale of fossil assemblages even in the absence of postmortem age-frequency data (Tomašových et al. 2023)[3]. On this basis, we analyzed the ^{210}Pb age models published in Armenteros et al (2021)[38] from the same seagrass sites in GGuanahacabibes looking for evidence about time averaging in our study. The age models based solely on sediments and published in Armenteros et al. (2021) [387] have the advantage that samples were taken at short distances (< 100 m) of sites in current study and using techniques (barrel corer) that effectively samples the top ten centimeters of sediment.

Formatted: Font: (Default) Times New Roman, 12 pt, English (United States)

Formatted: Font: (Default) Times New Roman, 12 pt, English (United States)

Formatted: Font: (Default) Times New Roman, 12 pt, English (United States)

Formatted: Font: (Default) Times New Roman, 12 pt, English (United States)

Formatted: Font: (Default) Times New Roman, 12 pt, English (United States)

We estimated that the temporal framework spanned by the death assemblages in our study was about 100 years based on two lines of evidence. First, the top ten centimeters of sediments at sites GG05, GG06, and GG09a spanned a period (relative to 2014, when the sampling was done) of 58, 99, and 81 years, respectively~~ (Armenteros et al. 2021)~~ [3~~8~~7]. Second, time averaging will tend to be low if stratigraphic resolution (and sedimentation rate) is very high, regardless of mixing depths and the rate of disintegration~~ (Tomašových et al. 2023)~~ [3]. In ~~G~~Guanahacabibes, the sedimentation rate was high as assessed from mass accumulation rates and dating. The temporal framework of about 100 years in our study supports the value of death assemblages to analyze the diversity of mollusk assemblages in the ecological time.

Our evidence also suggests that time averaging was lower in seagrass meadows compared to reef sands due to lower reworking and higher sedimentation rate ~~(Kidwell 2013)~~[1]. In reef sands the time averaging probably spanned a longer period because they were like open shelf environments in terms of hydrodynamics with stronger reworking and lower sedimentation rate ~~(Kidwell and Bosence 1991)~~[5]. Even when the time averaging in the ~~coral~~ reef sands was probably higher compared to seagrass, we hypothesize that in both habitat types the assemblage composition have been not long-time averaged due to the high species-, phylogenetic- and functional β-diversities [2, ~~(. Latter has been reported by Kidwell and Tomašových (2013,) and Zuschin and Ebner (2015~~6867~~)~~].

~~Live and death assemblages~~

Formatted: Font: 18 pt

It was interesting to explore the relationship between dead and alive assemblages of data collected from the same samples. This issue is closely related to one taphonomic process: the time averaging. Nevertheless, we did not apply a rigorous live-dead analysis (e.g., as in Tomašových et al. 2023) because of the lack of stratigraphic data and age models (see below).

The living assemblage was largely represented in the death assemblage: 76% of living species occurred in the death assemblages of both gulfs. As stated by Kidwell and Bosence (1991) and in agreement with our findings, death assemblages have higher species richness than any single census of live shelly fauna owing to the time-averaging of successive community states. Addition of allochthonous shells may also increase the species richness of the death assemblage (Kidwell and Bosence 1991), but we think this effect is negligible in our study given the high β-diversity and the concordance between

taxonomic/functional structure and habitat type (Albano and Sabelli 2011, Davis et al. 2018, Casebolt and Kowalewski 2018).

There were a few mollusk species exclusively alive that did not occur in the death assemblages of the same site; but they were detected at other sites in the same gulf; for instance, *Americardia media*, *Caecum regulare*, *Codakia orbicularis*, *Eulithidium thalassicola*, *Granulina ovuliformis*, *Granulina sp*., *Limaria pellucida*, *Rissoina krebsii*, and *Teinostoma parvicallum*. Even more, some species were exclusively alive in one gulf but found in the death assemblage of the other gulf: for instance, *Angulus sp*., *Atys spp*., *Caecum spp*., and *Phlyctiderma semiasperum*. This indicated that the “missed species” in the death assemblage were present in the regional species pool of the area as their geographic distribution includes the Gulf of Mexico and the Caribbean Central America (Mikkelsen and Bieler 2008). But still a few alive species were completely missed from the death assemblages: for

instance, *Amphithalamus rauli*, *Caecum* cf. *debile*, *Cumingia sp.*, *Cylindrobulla beauii*, *Dallocardia muricata*, *Ervilia sp.*, *Euvola ziczac*, *Galeommatidae sp. 1*, *Phacoides pectinatus*, *Solemya occidentalis*, *Teinostoma* sp., and *Thracia sp. 1*. The different preservation potential of shells may explain this finding since some of these species were particularly fragile and postmortem processes such as physical fragmentation and dissolution could remove them from the sedimentary record (Kidwell and Bosence 1991).

Our findings revealed that death assemblage contains a substantial fraction of the whole sympatric assemblage supporting its value to assess the diversity and assemblage structure of the whole assemblage (i.e., live + dead mollusks). Nevertheless, further methodological steps can maximize the available information. For instance, establish a sampling scheme that could cover as much as

possible the region under study and make a carefully preservation, storage, and processing of the shells in the laboratory.

The process of time averaging explains the larger richness in death assemblages (Kidwell and Bosence 1991), but also adds a source of uncertainty about the temporal matching between death and live assemblages. The extension of the time averaging depends on some non-exclusive processes such as (Kidwell 2013): (i) the intrinsic durability of the remains, (ii) the extent of the physical and biological reworking processes that can bury or exhume the remains within the mixed layer of sediments, and (iii) the sedimentation rates.

An accurate estimation of the stratigraphic resolution and time averaging in the Holocene-Anthropocene record ultimately requires that age models are coupled with post-mortem age-frequency distributions of shells dated by amino-acid racemization, ^{14}C or U-Th (Tomašových et al. 2023).

Nevertheless, latter authors also stated that simple age models for cores thus still can be informative about the temporal scale of fossil assemblages even in the absence of postmortem age-frequency data (Tomašových et al. 2023). On this basis, we analyzed the ^{210}Pb age models published in Armenteros et al (2021) from the same seagrass sites in GG looking for evidence about time averaging in our study. The age models based solely on sediments and published in Armenteros et al. (2021) have the advantage that samples were taken at short distances (< 100 m) of sites in current study and using techniques (barrel corer) that effectively samples the top ten centimeters of sediment.

We estimated that the temporal framework spanned by the death assemblages in our study was about 100 years based on two lines of evidence. First, the top ten centimeters of sediments at sites GG05, GG06, and GG09a spanned a period (relative to 2014, when the sampling was done) of 58, 99, and 81 years, respectively

(Armenteros et al. 2021). Second, time averaging will tend to be low if stratigraphic resolution (and sedimentation rate) is very high, regardless of mixing depths and the rate of disintegration (Tomašových et al. 2023). In GG, the sedimentation rate was high as assessed from mass accumulation rates and dating. The temporal framework of about 100 years in our study supports the value of death assemblages to analyze the diversity of mollusk assemblages in the ecological time.

Many species found “dead only” are not exotic or out-of-habitat, but simply have not yet been sampled live because of patchy and/or ephemeral population suggesting that the nature of habitat influences the matching between death and alive assemblages (Kidwell and Bosence 1991). In our study, seagrass meadows showed lower contribution (range: 92%–100%) of death to whole assemblage, when compared with coral reefs (range: 95%–100%) (Table 2). This suggests, in agreement with (Kidwell 2013) that

~~time averaging was lower in seagrass meadows due to lower reworking and higher sedimentation rate. Additional evidence was given by the dominance of *Parvilucina* spp in both alive and death assemblages at seagrass sites GG05, GG06, and GG09. In coral reefs the time averaging probably spanned a longer period because they were like open shelf environments in terms of hydrodynamics with stronger reworking and lower sedimentation rate (Kidwell and Bosence 1991). Even when the time averaging in the coral reefs was probably higher compared to seagrass, we hypothesize that in both habitat types the assemblage composition have been not long-time averaged due to the high species-, phylogenetic- and functional β-diversities. Latter has been reported by Kidwell and Tomašových (2013) and Zuschin and Ebner (2015).~~

~~Shelf and deep-sea species~~

Peraza-Escarrá et al. (2022) reported the occurrence of shelf mollusk species in the northwestern slope (about 1500 m depth) of Cuba. They proposed a downslope transport of materials (including shells) from the shelf to the deep sea. This finding is related to a second taphonomic process: postmortem transport of shells. In death assemblages, transport does tend to select shells of small-bodied, free-living epifauna and very shallow burrowing infauna (Kidwell and Bosence 1991). Given that our samples were dominated by these small-bodied shells, we should evaluate the importance of this process.

Allochthonous mollusk assemblages are most common in steeply sloped or episodically high-energy settings and these deposits are usually identifiable by their distinctive sedimentary structures and shell concentrations (Kidwell and Bosence 1991). Here we obtained evidence of this downslope transport from the Gulf of Guanahacabibes to the deep-sea site off (i.e., SL-38-750).

~~However, the magnitude of this downslope transport is not easy to evaluate because shell abundance in the deep sea was not quantified in Peraza-Escarrá et al. (2022).~~

~~As expected, the distance from the coastal site was a predictor of the occurrence of certain species at the deep-sea site. However, the shelf species detected in the deep sea were not dominant at all in the coastal sampled sites suggesting three non-exclusive explanations: (i) the dominant coastal species were transported as well but were either not detected in our limited deep-sea sampling using multicore; (ii) shells were mostly destroyed by post-mortem processes such as fragmentation and dissolution; and (iii) the transport was truly very limited and therefore affected by high level of randomness. Disentangling the explanation of this pattern requires further sampling for collecting more data in coastal sites in the proximity of the deep-sea sites sampled by Peraza-Escarrá et al. (2022). Also, it deserves further studies to understand the~~

Formatted: Font: 18 pt, English (United States)

Formatted: Font: 18 pt

Formatted: Font: 18 pt, English (United States)

Formatted: Font: 18 pt

~~potential effects of pollutants from the coastal zone (e.g., microplastics, petroleum, heavy metals) into the deep sea.~~

Conclusions

At regional scale, diversity and community structure between gulfs showed many similarities ~~(e.g., richness, β-diversity, taxonomic composition)~~ likely because geological and geographical commonalities. Habitat type had a strong effect on all the diversity metrics of assemblages. ~~Coral r~~Reef sands had higher species, phylogenetic, and functional richness than seagrass meadows probably influenced by more favorable productivity-disturbance balance. The ~~A~~assemblage ~~structure~~taxonomic composition was distinctive for each habitat type suggesting a limited lateral transport of dead shells between adjacent sites. Functional ~~structure~~ composition based on biological traits reflected niche properties of species adapted to each habitat. ~~Coral r~~Reef sands were characterized by epifaunal herbivores (mostly gastropods) adapted to feed on microphytobenthos growing in sandy spots and bisally attached bivalves adapted to intense hydrodynamics. In seagrass meadows, hydrodynamic regime influenced the functional structured, with dominance of suspensivores in exposed sites in ~~GB~~ Batabanó and dominance of chemosynthetic infaunal bivalves in ~~G~~Guanahacabibes where oxygen replenishment is more limited. On a local scale, sites had moderate differences because of small-scale environmental variability. Two seagrass sites ~~(GG06 and GG09)~~ closer to the coastline showed evidence of impact due to eutrophication and sedimentation. Time averaging was likely in the order of 100 years in our samples, with stronger effects in -reef sands compared to seagrass meadows. Our research provides evidence of the

highoutstanding taxonomically and functionally diverseity of Our research provides evidence of a highlyDdeath mollusk assemblages were taxonomically and functionally diverse across the two studied gulfs in tropical coastal sediments as resulted of the influence of scale-related processes and the habitat type.s of the

Death assemblages contained a substantial fraction of the sympatric whole assemblage supporting its value for assessing the diversity and assemblage structure of the whole assemblage (i.e., live + dead mollusks). Time averaging contributed to the large richness in death assemblages reported in our study and it was in the order of 100 years as estimated from ^{210}Pb age models and sedimentation rates in Armenteros et al. (2021). Habitat type (seagrass versus coral reef) likely influenced the magnitude of time averaging reflected by contribution of death to whole assemblage. Assemblages in seagrass meadows had lower death-to-whole contribution, when compared to coral reefs, due to less physical reworking and higher sedimentation rate; both features could reduce the time averaging of death assemblages. There is evidence of shelf-to-slope transport, but strong β-diversity patterns and coherent taxonomic and functional structure suggest that lateral out of habitat transport of shells within both gulfs is rather limited. Distance was a significant predictor of richness of shelf species in the deep sea. The potential downslope transport of pollutants from the coastal area deserves further studies given the fragility of deep-sea ecosystems.

Formatted: English (United States)

Acknowledgements

R Peraza-Escarrá thanks to PhD Graduate Program "Ciencias del Mar y Limnología" at Instituto de Ciencias del Mar y Limnología (UNAM) and to Consejo Nacional de Humanidades, Ciencias y Tecnologías (CONAHCYT) ~~for a PhD scholarship (CVU_1080567)~~. ~~M Armenteros thanks to CONAHCYT for a postdoctoral fellowship (CVU_982475). This research was supported partially by The Ocean Foundation through the "Proyecto Tres Golfos".~~ We acknowledge the crew of the R/V Felipe Poey. We ~~acknowledge~~ thank the Centro de Estudios Ambientales de Cienfuegos, particularly Misael Díaz-Asencio and Lisbet Díaz-Asencio, for sediment analyses. We thank Dania Saladrigas and Lorena González for sample processing, Adrián Martínez for providing the map, and Fernando Bretos and Daria Siciliano for their support in the field and technical assistance. The collection of sediment samples in Cuban waters was made under the permit LH112-AN-(23)-2014 granted by Centro de Inspección y Control Ambiental (CICA). We acknowledge very much the comments by Susan Kidwell and one anonymous reviewer that improved the manuscript.

Formatted: Font: 18 pt, English (United States)

Formatted: English (United States)

Author contribution

RPE: Data Curation, Formal Analysis, Investigation, Writing - Original Draft, Preparation, and Writing - Review & Editing. MA: Conceptualization, Formal Analysis, Funding Acquisition, Resources, Supervision, Writing - Original Draft Preparation, and Writing - Review & Editing. RFG: Data Curation, and Writing - Review & Editing. AG: Conceptualization, Funding Acquisition, Resources, Supervision, Writing - Original Draft Preparation, and Writing - Review & Editing.

Formatted: Font: 18 pt

Formatted: Font: 16 pt

References

Formatted: Font: 18 pt

[1] Kidwell SM. Time- averaging and fidelity of modern death assemblages: building a taphonomic foundation for conservation palaeobiology.- Paleontology. 2013;56: 487–522. doi: 10.1111/pala.12042.

[2] Kidwell SM, Tomašových A. Implications of time-averaged death assemblages for ecology and conservation biology.- Annu Rev Ecol Evol Syst. 2013;44: 539–563. doi: 10.1146/annurev-ecolsys-110512-135838.

[3] Tomašových A, Dominici S, Nawrot R, Zuschin M. Temporal scales, sampling designs, and age distributions in marine conservation palaeobiology.- Geol Soc Spec Publ. 2023;529: SP529–2023. doi: 10.1144/SP529-2022-361.

[4] Casebolt S, Kowalewski M. Mollusk shell assemblages as archives of spatial structuring of benthic communities around subtropical islands. Estuar Coast Shelf Sci. 2018;215: 132–43. doi: 10.1016/j.ecss.2018.09.023.

[5] Kidwell SM, Bosence D. Taphonomy and time-averaging of marine shelly faunas. In: Allison PA, Briggs D (Eds.), Taphonomy: Releasing the data locked in the fossil record. Plenum Publishers. 1991. pp. 115–209.

[6] Zhang Z. Animal Biodiversity: An update of classification and diversity in 2013. Zootaxa. 2013;3707: 5–11. doi: 10.11646/zootaxa.3703.1.3.

[7] WoRMS. World Register of Marine Species. Available from https://www.marinespecies.org at VLIZ. Accessed 2023-04-17. doi: 10.14284/170.

[8] Grill B, Zuschin M. Modern shallow-to deep-water bivalve death assemblages in the Red Sea—ecology and biogeography. Palaeogeogr, Palaeoclimatol, Palaeoecol. 2001; 168: 75–96. doi: 10.1016/S0031-0182(00)00250-9.

[9] Tomašových A, Kidwell SM. Preservation of spatial and environmental gradients by death assemblages. Paleobiology 2009;35: 119–145. doi: 10.1666/07081.1.

[10] Weber K, Zuschin M. Delta-associated molluscan life and death assemblages in the northern Adriatic Sea: implications for paleoecology, regional diversity and conservation. Palaeogeogr Palaeoclimatol Palaeoecol. 2013;370: 77–91. doi: 10.1016/j.palaeo.2012.11.021.

[11] Peraza-Escarrá R, Armenteros M, Fernández-Garcés R, Murawski SA, Gracia A. Mollusk death assemblages from the deep slope off northwestern Cuba (Gulf of Mexico). Bull Mar Sci. 2022;98: 451–470. Doi: 10.5343/bms.2022.0006.

[12] Pavoine S, Bonsall MB. Measuring biodiversity to explain community assembly: A unified approach. Biol Rev. 2011;86: 792–812. Doi: 10.1111/j.1469-185X.2010.00171.x.

[13] Magurran AE. Measuring biological diversity. Oxford: Blackwell; 2004. 257 p.

[14] Magurran AE. Measuring biological diversity. Curr Biol. 2021;31: R1174–1177. Doi: 10.1016/j.cub.2021.07.049.

[15] Tucker CM, Cadotte MW, Carvalho SB, Davies TJ, Ferrier S, Fritz SA, et al. A guide to phylogenetic metrics for conservation, community ecology and macroecology. Biol Rev. 2017;92: 698–715. Doi: 10.1111/brv.12252.

[16] Mammola S, Carmona CP, Guillerme T, Cardoso P. Concepts and applications in functional diversity. Funct Ecol. 2021;35: 1869–1885. Doi: 10.1111/1365-2435.13882.

[17] Anderson MJ, Crist TO, Chase JM, Vellend M, Inouye BD, Freestone AL, et al. Navigating the multiple meanings of β diversity: a roadmap for the practicing ecologist. Ecol Lett. 2011;14: 19–28. Doi: 10.1111/j.1461-0248.2010.01552.x.

[18] Legendre P. Interpreting the replacement and richness difference components of beta diversity. Glob Ecol Biogeogr. 2014; 23: 1324–1334. Doi: 10.1111/geb.12207.

[19] Carvalho JC, Cardoso P, Gomes P. Determining the relative roles of species replacement and species richness differences in generating beta-diversity patterns. Glob Ecol Biogeogr. 2012;21: b760–771. Doi: 10.1111/j.1466-8238.2011.00694.x.

[20] Carvalho JC, Cardoso P, Borges PA V, Schmera D, Podani J. Measuring fractions of beta diversity and their relationships to nestedness: a theoretical and empirical comparison of novel approaches. Oikos. 2013;122: 825–834. Doi: 10.1111/j.1600-0706.2012.20980.x.

[21] Cardoso P, Rigal F, Carvalho JC, Fortelius M, Borges PA V, Podani J, et al. Partitioning taxon, phylogenetic and functional beta diversity into replacement and richness difference components. J Biogeogr. 2014;41: 749–761.

[22] Baselga A. Partitioning the turnover and nestedness components of beta diversity. Glob Ecol Biogeogr. 2010;19: 134–43. dDoi: 10.1111/j.1466-8238.2009.00490.x.

[23] Baselga A. The relationship between species replacement, dissimilarity derived from nestedness, and nestedness. Glob Ecol Biogeogr. 2012;21: 1223–32. d~~D~~oi: 10.1111/j.1466-8238.2011.00756.x.

[24] Baselga A, Leprieur F. Comparing methods to separate components of beta diversity. Methods Ecol Evol. 2015;6: 1069–79. d~~D~~oi: 10.1111/2041-210X.12388.

[25] Chao A, Chiu CH, Jost L. Unifying species diversity, phylogenetic diversity, functional diversity, and related similarity and differentiation measures through Hill numbers. Annu Rev Ecol Evol Syst. 2014;45:297–324. ~~D~~doi: 10.1146/annurev-ecolsys-120213-091540.

[26] Southwood TRE. Habitat, the templet for ecological studies? J Anim Ecol. 1977;46: 337–365.

[27] Ferraro SP. Ecological periodic tables: In principle and practice. Oikos 2013;122: 1541–53.

[28] Armenteros M, Saladrigas D, González-Casuso L, Estevez ED, Kowalewski M. The role of habitat selection on the diversity of ~~macrobenthic61i~~macrobenthic communities in three gulfs of the Cuban Archipelago. Bull Mar Sci. 2018;94: 249–68. ~~D~~doi: 10.5343/bms.2017.1013.

[29] Côté IM, Knowlton N. Coral reef ecosystems: a decade of discoveries. In: Bertness MD, Bruno JF, Silliman BR, Stachowicz JJ, editors. Marine community ecology and conservation. Sunderland: Sinauer Associates; 2013. Pp. 299–314.

[30] Duffy JE, Hughes AR, Moksnes P-O. Ecology of seagrass communities. In: Bertness MD, Bruno JF, Silliman BR, Stachowicz JJ, editors. Marine community ecology and conservation. Sunderland: Sinauer Associates; 2013. Pp. 271–297.

[31] Caballero Aragón H, Armenteros M, Perera Valderrama S, Rey Villiers N, Cobián Rojas D, Campos Verdecia K, Alcolado Menéndez PM. Ecological condition of coral reef assemblages in the Cuban Archipelago. Mar Biol Res. 2019;15: 61–73. dDoi: 10.1080/17451000.2019.1577557.

[32] Martínez-Daranas B, Suárez AM. An overview of Cuban seagrasses. Bull Mar Sci. 2018;94: 269–282. Ddoi: 10.5343/bms.2017.1014.

[33] Hoskins CW. Molluscan biofacies in calcareous sediments, Gulf of Batabanó, Cuba. Bull Am Assoc Pet Geol. 1964;48: 1680–1704.

[34] Capetillo-Piñar N, Villalejo-Fuerte MT, Tripp-Quezada A. Distinción taxonómica de los moluscos de fondos blandos del Golfo de Batabanó, Cuba. Lat Am J Aquat Res. 2015;43: 856–72. Doi: 10.3856/vol43-issue5-fulltext-6.

[365] Armenteros M, Díaz-Asencio M, Fernández-Garcés R, Eriksson M, Alonso-Hernández C, Sánchez-Cabeza J-A. Historical changes of sediments and mollusk assemblages in the Gulf of Batabanó (Caribbean Sea) in the twentieth century. Environ Monit Assess. 2012;184: 4709–4723. Doi: 10.1007/s10661-011-2296-z.

[376] Armenteros, M., Díaz-Asencio, M., Fernández-Garcés, R., Hernández, C. A., Helguera-Pedraza, Y., Bolaños-Alvarez, Y., Agraz-Hernández, C., & Sanchez-Cabeza, J.-A. One-century decline of mollusk diversity as consequence of accumulative anthropogenic disturbance in a

tropical estuary (Cuban Archipelago). Mar Pollut Bull. 2016;113:224–231. Doi: 10.1016/j.marpolbul.2016.09.023.

[3~~8~~7] Armenteros M, Díaz-Asencio M, Peraza-Escarrá R, Fernández-Garcés R, Martínez-Suárez A, Kenney WF, Brenner M. Mollusk death assemblages in ^{210}Pb-dated marine sediment cores reveal recent biotic changes in the Gulf of Guanahacabibes, NW Cuba. Mar Environ Res. 2021;171: 105477. Doi: 10.1016/j.marenvres.2021.105477.

[3~~9~~8] Buesa RJ. Annual benthic irradiance levels at the Northwestern Cuban shelf. Reg Stud Mar Sci. 2020;33: 100969. Doi: 10.1016/j.rsma.2019.100969.

[~~40~~39] Abbott RT. American Seashells. 2nd ed. New York: Van Nostrand Reinhold; 1974. 663 p.

[4~~1~~0] Mikkelsen PM, Bieler R. Seashells of southern Florida. Living marine mollusks of the Florida Keys and adjacent regions. Bivalves. New Jersey: Princeton University Press; 2008.

[4~~2~~1] Tunnell JW, Andrews J, Barrera NC, Moretzson F. Encyclopedia of Texas seashells: identification, ecology, distribution and history. Texas: Texas A&M University Press; 2010.

[4~~3~~2] Redfern C. Bahamian seashells: 1161 species from Abaco, Bahamas. Bahamian seashells. Com, Incorporated; 2013.

[4~~4~~3] Todd J. Bivalves; 2008. Gastropods; 2006. Database: Neogene Marine Biota of Tropical America (NMITA) [Internet]. Available from: https://nmita.rsmas.miami.edu/index.htm

Formatted: Superscript

[4~~5~~4] Rosenberg G, Moretzsohn F, García EF, Felder DL, Camp DK. Gastropoda (Mollusca) of the Gulf of Mexico. In: Felder DL, Camp DK, editors. Gulf of Mexico origins, waters, and biota, Vol. 1. Texas A&M University Press; 2009. Pp. 579–699.

[4~~6~~5] Turgeon DD, Lyons WG, Mikkelsen P, Rosenberg G, Moretzsohn F, Felder DL, Camp DK. Bivalvia (Mollusca) of the Gulf of Mexico. In: Felder DL, Camp DK, editors. Gulf of Mexico origins, waters, and biota, Vol. 1. Texas A&M University Press; 2009. Pp. 711–744.

[4~~7~~6] Hill MO. Diversity and evenness: a unifying notation and its consequences. Ecology 1973;54: 427–32. Doi: 10.2307/1934352.

[4~~8~~7] Hsieh TC, Ma KH, Chao A. iNEXT: an R package for rarefaction and extrapolation of species diversity (Hill numbers). Methods Ecol Evol. 2016;7: 1451–1456. Doi: 10.1111/2041-210X.12613.

[4~~9~~8] Faith DP. Conservation evaluation and phylogenetic diversity. Biol Conserv. 1992;61: 1–10. Doi: 10.1016/0006-3207(92)91201-3.

[~~50~~49] Cardoso P, Rigal F, Carvalho JC. BAT ~~-~~— Biodiversity Assessment Tools, an R package for the measurement and estimation of alpha and beta taxon, phylogenetic and functional diversity. Methods Ecol Evol. 2015;6: 232–236. Doi: 10.1111/2041-210X.12310.

[5~~1~~0] Petchey OL, Gaston KJ. Functional diversity (FD), species richness and community composition. Ecol Lett. 2002;5: 402–411. Doi: 10.1046/j.1461-0248.2002.00339.x.

[5~~2~~1] Legras N, Loiseau N, Gaertner J-C. Functional richness: Overview of indices and underlying concepts. Acta Oecol. 2018;87: 34–44. Doi: 10.1016/j.actao.2018.02.007.

[532] Streit RP, Bellwood DR. To harness traits for ecology, let's abandon 'functionality.' Trends Ecol Evol. 2022;3084: 1–10. Doi: 10.1016/j.tree.2022.11.009.

[543] Cardoso P, Rigal F, Borges PAV, Carvalho JC. A new frontier in biodiversity inventory: A proposal for estimators of phylogenetic and functional diversity. Methods Ecol Evol. 2014;5: 452–461. Doi: 10.1111/2041-210X.12173

[554] Cardoso P, Borges PAV, Veech JA. Testing the performance of beta diversity measures based on incidence data: the robustness to undersampling. Divers Distrib. 2009;15: 1081–1090. Doi: 10.1111/j.1472-4642.2009.00607.x.

[556] Clarke KR, Gorley RN, Somerfield PJ, Warwick RM. Change in marine communities: an approach to statistical analysis and interpretation. 3rd ed. Plymouth: PRIMER-E: Plymouth; 2014. 262 p.

[576] Anderson MJ, Gorley RN, Clarke KR. PERMANOVA+ for PRIMER: Guide to software and statistical methods. Massey: PRIMER-E Ltd; 2008. 218 p.

[587] Wickham H, Averick M, Bryan J, Chang W, McGowan LD, François R, Grolemundet G, et al. Welcome to the Tidyverse. J Open Source Soft. 2019;4: 1686. doi: 10.21105/joss.01686.

[598] Gotelli NJ, Colwell RK. Quantifying biodiversity: procedures and pitfalls in the measurement and comparison of species richness. Ecol Lett. 2001;4: 379–391. doi: 10.1046/j.1461-0248.2001.00230.x.

Formatted: Superscript

[~~60~~59] van der Plas F, Hennecke J, Chase JM, van Ruijven J, Barry KE. Universal beta-diversity – functioning relationships are neither observed nor expected. Trends Ecol Evol. 2023. doi: 10.1016/j.tree.2023.01.008.

[6~~1~~0] Costello MJ, Tsai P, Wong PS, Cheung AKL, Basher Z, Chaudhary C. Marine biogeographic realms and species endemicity. Nature Comm. 2017;8: 1057. doi: 10.1038/s41467-017-01121-2.

[6~~2~~1] Huston M. Biological diversity — The coexistence of species. Cambridge University Press; 1994. 681 p.

[6~~3~~2] Lluis-Riera M. Estudios hidrológicos del Golfo de Batabanó y de las aguas oceánicas adyacentes. Ser Oceanol. 1972;14: 1–46.

[6~~4~~3] Arriaza L, Rodas L, Simanca J, Lorenzo-Sánchez S, Milian DL, Romero P. Contribución a la gestión ambiental del Golfo de Batabanó, Cuba: Modelación numérica de corrientes marinas. Rev Invest Mar. 2008;29: 89–99.

[6~~5~~4] Baisre J. An overview of Cuban commercial marine fisheries: The last 80 years. Bull Mar Sci. 2018;94: 359–375. doi: 10.5343/bms.2017.1015.

[6~~6~~5] Altieri AH, Johnson MD, Swaminathan SD, Nelson HR, Gedan KB. Resilience of tropical ecosystems to ocean deoxygenation. Trends Ecol Evol. 2021;3: 227–238. doi: 10.1016/j.tree.2020.11.003.

[6~~7~~6] Virta L. Diversity of benthic diatoms in the Baltic Sea: alpha and beta diversity, environmental drivers, and diversity-biomass relationships. Mar Ecol Progr Ser. 2023;712: 35–47. doi: 10.3354/meps14320.

[6~~8~~7] Zuschin M, Ebner C. Compositional fidelity of death assemblages from a coral reef-associated tidal-flat and shallow subtidal lagoon in the northern Red Sea. Palaios. 2015;30(3): 181–191. doi: 10.2110/palo.2014.032.

[6~~9~~8] Bracchi VA, Meroni AN, Epis V, Basso D. Mollusk thanatocoenoses unravel the diversity of heterogeneous rhodolith beds (Italy, Tyrrhenian Sea). Diversity. 2023;15: 526. doi: 10.3390/d15040526.

[~~70~~69] Barrientos-Luján NA, Rodríguez-Zaragoza FA, and López-Pérez A. Richness, abundance and spatial heterogeneity of gastropods and bivalves in coral ecosystems across the Mexican tropical Pacific. J Molluscan Stud. 2021;87: eyab004. doi: 10.1093/mollus/eyab004.

[7~~1~~0] Hemminga MA, Duarte CM. Seagrass ecology. Cambridge University Press; 2000. 298 p.

[7~~2~~1] Kidwell SM. Ecological fidelity of open marine molluscan death assemblages: effects of post- mortem transportation, shelf health, and taphonomic inertia. Lethaia. 2008;4: 199–217. doi: 10.1111/j.1502-3931.2007.00050.x.

[7~~3~~2] Davis KA, Pawlak G, Monismith SG. Turbulence and coral reefs. Annu Rev Mar Sci. 2021;13: 343–373. doi: 10.1146/annurev-marine-042120-071823.

[743] Albano PG, Sabelli B. Comparison between death and living molluscs assemblages in a Mediterranean infralittoral off-shore reef. Palaeogeogr Palaeoclimatol Palaeoecol. 2011;310: 206–215. doi: 10.1016/j.palaeo.2011.07.012.

[754] MacIntyre HL, Geider RJ, Miller DC. 1996. Microphytobenthos: The ecological role of the “secret garden” of unvegetated, shallow-water marine habitats. I. Distribution, abundance and primary production. Estuaries 1996;19: 186–201.

[765] Miller DC, Geider RJ, MacIntyre HL. 1996. Microphytobenthos: The ecological role of the "secret garden” of unvegetated, shallow-water marine habitats. II. Role in sediment stability and shallow-water food webs. Estuaries 1996;19: 202–212.

[776] Jiménez-Ramos, R., Egea, L. G., Vergara, J. J., Bouma, T. J., & Brun, F. G. (2019). The role of flow velocity combined with habitat complexity as a top–down regulator in seagrass meadows. Oikos, 128, 64–76. doi: 10.1111/oik.05452.

[787] Witman JD, Etter RJ, Smith F. The relationship between regional and local species diversity in marine benthic communities: A global perspective. Proc Nat Acad Sci USA. 2004;101: 15664–15669. doi: 10.1073/pnas.0404300101.

[798] Hewitt JE, Thrush SF, Halliday J, Duffy C. The importance of small-scale habitat structure for maintaining beta diversity. Ecology 2005;86: 1619–1626. doi: 10.1890/04-1099.

-[8079] Creed JC, Kinupp M. Small scale change in mollusk diversity along a depth gradient in a seagrass bed off Cabo Frio, (Southeast Brazil). Braz J Oceanogr. 2011;59: 267–276.

[8~~1~~0] Davis T, Harasti D, Kelaher B, Smith SDA. Spatial and temporal variation in subtidal molluscan diversity amongst temperate estuarine habitats. Mar Ecol. 2017; 38: e12428. doi: 10.1111/maec.12428.

Formatted: Indent: Left: 0", Hanging: 0.25", Space Before: 6 pt, After: 0 pt, Line spacing: Double

[40] Abbott RT. American Seashells. 2nd ed. New York: Van Nostrand Reinhold; 1974. 663 p.

[74] Albano PG, Sabelli B. Comparison between death and living molluscs assemblages in a Mediterranean infralittoral off-shore reef. Palaeogeogr Palaeoclimatol Palaeoecol. 2011;310: 206–215. doi: 10.1016/j.palaeo.2011.07.012.

[66] Altieri AH, Johnson MD, Swaminathan SD, Nelson HR, Gedan KB. Resilience of tropical ecosystems to ocean deoxygenation. Trends Ecol Evol. 2021;3: 227–238. doi: 10.1016/j.tree.2020.11.003.

[17] Anderson MJ, Crist TO, Chase JM, Vellend M, Inouye BD, Freestone AL, et al. Navigating the multiple meanings of β diversity: a roadmap for the practicing ecologist. Ecol Lett. 2011;14: 19–28. doi: 10.1111/j.1461-0248.2010.01552.x.

[57] Anderson MJ, Gorley RN, Clarke KR. PERMANOVA+ for PRIMER: Guide to software and statistical methods. Massey: PRIMER-E Ltd; 2008. 218 p.

[36] Armenteros M, Díaz-Asencio M, Fernández-Garcés R, Eriksson M, Alonso-Hernández C, Sánchez-Cabeza J-A. Historical changes of sediments and mollusk assemblages in the Gulf of Batabanó (Caribbean Sea) in the twentieth century. Environ Monit Assess. 2012;184: 4709–4723. doi: 10.1007/s10661-011-2296-z.

[28] Armenteros M, Saladrigas D, Lorena González-Casuso L, Estevez ED, Kowalewski M. The role of habitat selection on the diversity of macrobenthic communities in three gulfs of the Cuban Archipelago. Bull Mar Sci. 2018;94: 249–68. doi: 10.5343/bms.2017.1013.

Formatted: English (United States)

Formatted: English (United States)

Field Code Changed

Formatted: English (United States)

Formatted: Spanish (Mexico)

[37] Armenteros, M., Díaz-Asencio, M., Fernández-Garcés, R., Hernández, C. A., Helguera-Pedraza, Y., Bolaños-Alvarez, Y., Agraz-Hernández, C., & Sanchez-Cabeza, J.-A. (2016). One-century decline of mollusk diversity as consequence of accumulative anthropogenic disturbance in a tropical estuary (Cuban Archipelago). Marine Pollution Bulletin. 2016, ;113(:224–231). https://doi.org/10.1016/j.marpolbul.2016.09.023

[38] Armenteros M, Díaz-Asencio M, Peraza-Escarrá R, Fernández-Garcés R, Martínez-Suárez A, Kenney WF, Brenner M. Mollusk death assemblages in ^{210}Pb-dated marine sediment cores reveal recent biotic changes in the Gulf of Guanahacabibes, NW Cuba. Mar Environ Res. 2021;171: 105477. doi: 10.1016/j.marenvres.2021.105477.

[64] Arriaza L, Rodas L, Simanca J, Lorenzo-Sánchez S, Milian DL, Romero P. Contribución a la gestión ambiental del Golfo de Batabanó, Cuba: Modelación numérica de corrientes marinas. Rev Invest Mar. 2008;29: 89–99.

[65] Baisre J. An overview of Cuban commercial marine fisheries: The last 80 years. Bull Mar Sci. 2018;94: 359–375. doi: 10.5343/bms.2017.1015.

[70] Barrientos-Luján NA, Rodríguez-Zaragoza FA, and López-Pérez A. Richness, abundance and spatial heterogeneity of gastropods and bivalves in coral ecosystems across the Mexican tropical Pacific. J Molluscan Stud. 2021;87: eyab004. doi: 10.1093/mollus/eyab004.

[22] Baselga A. Partitioning the turnover and nestedness components of beta diversity. Glob Ecol Biogeogr. 2010;19: 134–43. doi: 10.1111/j.1466-8238.2009.00490.x.

[23] Baselga A. The relationship between species replacement, dissimilarity derived from nestedness, and nestedness. Glob Ecol Biogeogr. 2012;21: 1223–32. doi: 10.1111/j.1466-8238.2011.00756.x.

Formatted: Superscript

Formatted: English (United States)

[24] Baselga A, Leprieur F. Comparing methods to separate components of beta diversity. Methods Ecol Evol. 2015;6: 1069–79. doi: 10.1111/2041-210X.12388.

[69] Bracchi VA, Meroni AN, Epis V, Basso D. Mollusk Thanatocoenoses thanatocoenoses Unravel unravel the Diversity diversity of Heterogeneous heterogeneous Rhodolith rhodolith Beds beds (Italy, Tyrrhenian Sea). Diversity. 2023;15: 526. doi: .org/10.3390/d15040526.

[39] Buesa RJ. Annual benthic irradiance levels at the Northwestern Cuban shelf. Reg Stud Mar Sci. 2020;33: 100969. doi: 10.1016/j.rsma.2019.100969.

[31] Caballero Aragón H, Armenteros M, Perera Valderrama S, Rey Villiers N, Cobián Rojas D, Campos Verdecia K, Alcolado Menéndez PM. Ecological condition of coral reef assemblages in the Cuban Archipelago. Mar Biol Res. 2019;15: 61–73. doi: 10.1080/17451000.2019.1577557.

[34] Capetillo-Piñar N, Villalejo-Fuerte MT, Tripp-Quezada A. Distinción taxonómica de los moluscos de fondos blandos del Golfo de Batabanó, Cuba. Lat Am J Aquat Res. 2015;43: 856–72. doi: 10.3856/vol43-issue5-fulltext-6.

[55] Cardoso P, Borges PAV, Veech JA. Testing the performance of beta diversity measures based on incidence data: the robustness to undersampling. Divers Distrib. 2009;15: 1081–1090. doi: 10.1111/j.1472-4642.2009.00607.x.

[54] Cardoso P, Rigal F, Borges PAV, Carvalho JC. A new frontier in biodiversity inventory: A proposal for estimators of phylogenetic and functional diversity. Methods Ecol Evol. 2014a;5: 452–461. doi: 10.1111/2041-210X.12173

[21] Cardoso P, Rigal F, Carvalho JC, Fortelius M, Borges PA V, Podani J, et al. Partitioning taxon, phylogenetic and functional beta diversity into replacement and richness difference components. J Biogeogr. 2014b;41: 749–761.

Field Code Changed

Formatted: Portuguese (Brazil)

Formatted: Portuguese (Brazil)

Formatted: Portuguese (Brazil)

Formatted: Portuguese (Brazil)

Formatted: Portuguese (Brazil)

Formatted: Portuguese (Brazil)

[50] Cardoso P, Rigal F, Carvalho JC. BAT - Biodiversity Assessment Tools, an R package for the measurement and estimation of alpha and beta taxon, phylogenetic and functional diversity. Methods Ecol Evol. 2015;6: 232–236. doi: 10.1111/2041-210X.12310

[19] Carvalho JC, Cardoso P, Gomes P. Determining the relative roles of species replacement and species richness differences in generating beta-diversity patterns. Glob Ecol Biogeogr. 2012;21: b760–771. doi: 10.1111/j.1466-8238.2011.00694.x.

[20] Carvalho JC, Cardoso P, Borges PA V, Schmera D, Podani J. Measuring fractions of beta diversity and their relationships to nestedness: a theoretical and empirical comparison of novel approaches. Oikos. 2013;122: 825–834. doi: 10.1111/j.1600-0706.2012.20980.x.

[4] Casebolt S, Kowalewski M. Mollusk shell assemblages as archives of spatial structuring of benthic communities around subtropical islands. Estuar Coast Shelf Sci. 2018;215: 132–43. doi: 10.1016/j.ecss.2018.09.023.

[25] Chao A, Chiu CH, Jost L. Unifying species diversity, phylogenetic diversity, functional diversity, and related similarity and differentiation measures through Hill numbers. Annu Rev Ecol Evol Syst. 2014;45:297–324. doi: 10.1146/annurev-ecolsys-120213-091540

[56] Clarke KR, Gorley RN, Somerfield PJ, Warwick RM. Change in marine communities: an approach to statistical analysis and interpretation. 3rd ed. Plymouth: PRIMER-E: Plymouth; 2014. 262 p.

[61] Costello MJ, Tsai P, Wong PS, Cheung AKL, Basher Z, Chaudhary C. Marine biogeographic realms and species endemicity. Nature Comm. 2017;8: 1057. doi: 10.1038/s41467-017-01121-2.

Formatted: English (United States)

[29] Côté IM, Knowlton N. Coral reef ecosystems: a decade of discoveries. In: Bertness MD, Bruno JF, Silliman BR, Stachowicz JJ, editors. Marine community ecology and conservation. Sunderland: Sinauer Associates; 2013. pp. 299–314.

[80] Creed JC, Kinupp M. Small scale change in mollusk diversity along a depth gradient in a seagrass bed off Cabo Frio, (Southeast Brazil). Braz J Oceanogr. 2011;59: 267–276.

[73] Davis KA, Pawlak G, Monismith SG. Turbulence and coral reefs. Annu Rev Mar Sci. 2021;13: 343–373. doi: 10.1146/annurev-marine-042120-071823.

[81] Davis T, Harasti D, Kelaher B, Smith SDA. Spatial and temporal variation in subtidal molluscan diversity amongst temperate estuarine habitats. Mar Ecol. 2017; 38: e12428. doi: 10.1111/maec.12428.

[30] Duffy JE, Hughes AR, Moksnes P-O. Ecology of seagrass communities. In: Bertness MD, Bruno JF, Silliman BR, Stachowicz JJ, editors. Marine community ecology and conservation. Sunderland: Sinauer Associates; 2013. pp. 271–297.

[49] Faith DP. Conservation evaluation and phylogenetic diversity. Biol Conserv. 1992;61: 1–10. doi: 10.1016/0006-3207(92)91201-3.

[27] Ferraro SP. Ecological periodic tables: In principle and practice. Oikos 2013;122: 1541–53.

[59] Gotelli NJ, Colwell RK. Quantifying biodiversity: procedures and pitfalls in the measurement and comparison of species richness. Ecol Lett. 2001;4: 379–391. doi: org/10.1046/j.1461-0248.2001.00230.x.

[8] Grill B, Zuschin M. Modern shallow-to deep-water bivalve death assemblages in the Red Sea — ecology and biogeography. Palaeogeogr, Palaeoclimatol, Palaeoecol. 2001; 168: 75–96. doi: 10.1016/S0031-0182(00)00250-9.

Formatted: French (France)

Formatted: Portuguese (Brazil)

Formatted: Portuguese (Brazil)

Formatted: English (United States)

Formatted: Portuguese (Brazil)

Formatted: Portuguese (Brazil)

Field Code Changed

Formatted: Portuguese (Brazil)

Field Code Changed

Formatted: Portuguese (Brazil)

Formatted: Portuguese (Brazil)

[71] Hemminga MA, Duarte CM. Seagrass ecology. Cambridge University Press; 2000. 298 p.

[79] Hewitt JE, Thrush SF, Halliday J, Duffy C. The importance of small-scale habitat structure for maintaining beta diversity. Ecology 2005;86: 1619–1626. doi: 10.1890/04-1099.

[47] Hill MO. Diversity and evenness: a unifying notation and its consequences. Ecology 1973;54: 427–32. doi: 10.2307/1934352.

[33] Hoskins CW. Molluscan biofacies in calcareous sediments, Gulf of Batabanó, Cuba. Bull Am Assoc Pet Geol. 1964;48: 1680–1704.

[48] Hsieh TC, Ma KH, Chao A. iNEXT: an R package for rarefaction and extrapolation of species diversity (Hill numbers). Methods Ecol Evol. 2016;7: 1451–1456. doi: 10.1111/2041-210X.12613.

[62] Huston M. Biological diversity — The coexistence of species. Cambridge University Press; 1994. 681 p.

[77] Jiménez-Ramos, R., Egea, L. G., Vergara, J. J., Bouma, T. J., & Brun, F. G. (2019). The role of flow velocity combined with habitat complexity as a top–down regulator in seagrass meadows. Oikos, 128, 64–76. dDoi: 10.1111/oik.05452

[72] Kidwell SM. Time-averaged molluscan death assemblages: Palimpsests of richness, snapshots of abundance. Geology. 2002;30: 803–806. doi: 10.1130/0091-7613(2002)030%3C0803:TAMDAP%3E2.0.CO;2.

Kidwell SM. Ecological fidelity of open marine molluscan death assemblages: effects of post-mortem transportation, shelf health, and taphonomic inertia. Lethaia. 2008;4: 199–217. doi: 10.1111/j.1502-3931.2007.00050.x.

Formatted: Portuguese (Brazil)

Field Code Changed

[1] Kidwell SM. Time- averaging and fidelity of modern death assemblages: building a taphonomic foundation for conservation palaeobiology. Paleontology. 2013;56: 487–522. doi: 10.1111/pala.12042.

[5] Kidwell SM, Bosence D. Taphonomy and time-averaging of marine shelly faunas. In: Allison PA, Briggs D (Eds.), Taphonomy: Releasing the data locked in the fossil record. Plenum Publishers. 1991. pp. 115–209.

[2] Kidwell SM, Tomašových A. Implications of time-averaged death assemblages for ecology and conservation biology. Annu Rev Ecol Evol Syst. 2013;44: 539–563. doi: 10.1146/annurev-ecolsys-110512-135838.

[18] Legendre P. Interpreting the replacement and richness difference components of beta diversity. Glob Ecol Biogeogr. 2014; 23: 1324–1334. doi: 10.1111/geb.12207.

[52] Legras N, Loiseau N, Gaertner J-C. Functional richness: Overview of indices and underlying concepts. Acta Oecol. 2018;87: 34–44. doi: 10.1016/j.actao.2018.02.007.

[63] Lluis-Riera M. Estudios hidrológicos del Golfo de Batabanó y de las aguas oceánicas adyacentes. Ser Oceanol. 1972;14: 1–46.

[75] MacIntyre HL, Geider RJ, Miller DC. 1996. Microphytobenthos: The ecological role of the “secret garden” of unvegetated, shallow-water marine habitats. I. Distribution, abundance and primary production. Estuaries 1996;19: 186–201.

[13] Magurran AE. Measuring biological diversity. Oxford: Blackwell; 2004. 257 p.

[14] Magurran AE. Measuring biological diversity. Curr Biol. 2021;31: R1174–1177. doi: 10.1016/j.cub.2021.07.049.

Field Code Changed

Field Code Changed

Formatted: Portuguese (Brazil)

Formatted: Portuguese (Brazil)

[16] Mammola S, Carmona CP, Guillerme T, Cardoso P. Concepts and applications in functional diversity. Funct Ecol. 2021;35: 1869–1885. doi: 10.1111/1365-2435.13882.

[32] Martínez-Daranas B, Suárez AM (2018). An overview of Cuban seagrasses. Bulletin of Marine Science,. 2018;94(2),: 269–282. https://doi.org/10.5343/bms.2017.1014

[41] Mikkelsen PM, Bieler R. Seashells of southern Florida. Living marine mollusks of the Florida Keys and adjacent regions. Bivalves. New Jersey: Princeton University Press; 2008.

[76] Miller DC, Geider RJ, MacIntyre HL. 1996. Microphytobenthos: The ecological role of the "secret garden" of unvegetated, shallow-water marine habitats. II. Role in sediment stability and shallow-water food webs. Estuaries 1996;19: 202–212.

[12] Pavoine S, Bonsall MB. Measuring biodiversity to explain community assembly: A unified approach. Biol Rev. 2011;86: 792–812. doi: 10.1111/j.1469-185X.2010.00171.x.

[11] Peraza-Escarrá R, Armenteros M, Fernández-Garcés R, Murawski SA, Gracia A. Mollusk death assemblages from the deep slope off northwestern Cuba (Gulf of Mexico). Bull Mar Sci. 2022;98: 451–470. doi: 10.5343/bms.2022.0006.

[51] Petchey OL, Gaston KJ. Functional diversity (FD), species richness and community composition. Ecol Lett. 2002;5: 402–411. doi: 10.1046/j.1461-0248.2002.00339.x.

[43] Redfern C. Bahamian seashells: 1161 species from Abaco, Bahamas. Bahamian seashells. com, Incorporated; 2013.

[45] Rosenberg G, Moretzsohn F, García EF, Felder DL, Camp DK. Gastropoda (Mollusca) of the Gulf of Mexico. In: Felder DL, Camp DK, editors. Gulf of Mexico origins, waters, and biota, Vol. 1. Texas A&M University Press; 2009. pp. 579–699.

[26] Smith SD. Interpreting molluscan death assemblages on rocky shores: are they representative of the regional fauna? J Exp Mar Biol Ecol. 2008;366: 151–159. doi: 10.1016/j.jembe.2008.07.019.

Southwood TRE. Habitat, the templet for ecological studies? J Anim Ecol. 1977;46: 337–365.

[53] Streit RP, Bellwood DR. To harness traits for ecology, let's abandon 'functionality.' Trends Ecol Evol. 2022;3084: 1–10. doi: 10.1016/j.tree.2022.11.009.

[9] Tomašových A, Kidwell SM. Preservation of spatial and environmental gradients by death assemblages. Paleobiology 2009;35: 119–145. doi: 10.1666/07081.1.

[3] Tomašových A, Dominici S, Nawrot R, Zuschin M. Temporal scales, sampling designs, and age distributions in marine conservation palaeobiology. Geol Soc Spec Publ. 2023;529: SP529–20223. doi: 10.1144/SP529-2022-361.

[15] Tucker CM, Cadotte MW, Carvalho SB, Davies TJ, Ferrier S, Fritz SA, et al. A guide to phylogenetic metrics for conservation, community ecology and macroecology. Biol Rev. 2017;92: 698–715. doi: 10.1111/brv.12252.

[42] Tunnell JW, Andrews J, Barrera NC, Moretzson F. Encyclopedia of Texas seashells: identification, ecology, distribution and history. Texas: Texas A&M University Press; 2010.

[44] Todd J. Bivalves; 2008. Gastropods; 2006. Database: Neogene Marine Biota of Tropical America (NMITA) [Internet]. Available from: https://nmita.rsmas.miami.edu/index.htm . Bivalves. Gastropods.

[46] Turgeon DD, Lyons WG, Mikkelsen P, Rosenberg G, Moretzsohn F, Felder DL, Camp DK. Bivalvia (Mollusca) of the Gulf of Mexico. In: Felder DL, Camp DK, editors. Gulf of Mexico origins, waters, and biota, Vol. 1. Texas A&M University Press; 2009. pp. 711–744.

Field Code Changed

Field Code Changed

Field Code Changed

Field Code Changed

Formatted: English (United States)

[60] van der Plas F, Hennecke J, Chase JM, van Ruijven J, Barry KE. Universal beta-diversity–functioning relationships are neither observed nor expected. Trends Ecol Evol. 2023. doi: 10.1016/j.tree.2023.01.008.

[67] Virta L. Diversity of benthic diatoms in the Baltic Sea: alpha and beta diversity, environmental drivers, and diversity-biomass relationships. Mar Ecol Progr Ser. 2023;712: 35–47. doi: 10.3354/meps14320.

[10] Weber K, Zuschin M. Delta-associated molluscan life and death assemblages in the northern Adriatic Sea: implications for paleoecology, regional diversity and conservation. Palaeogeogr Palaeoclimatol Palaeoecol. 2013;370: 77–91. doi: 10.1016/j.palaeo.2012.11.021.

[58] Wickham H, Averick M, Bryan J, Chang W, McGowan LD, François R, Grolemundet G, et al. Welcome to the Tidyverse. J Open Source Soft. 2019;4: 1686. doi: 10.21105/joss.01686

[78] Witman JD, Etter RJ, Smith F. The relationship between regional and local species diversity in marine benthic communities: A global perspective. Proc Nat Acad Sci USA. 2004;101: 15664–15669. doi: 10.1073/pnas.0404300101.

[7] WoRMS. World Register of Marine Species. Available from https://www.marinespecies.org at VLIZ. Accessed 2023-04-17. doi: 10.14284/170.

[6] Zhang Z. Animal Biodiversity: An update of classification and diversity in 2013. Zootaxa. 2013;3707: 5–11. doi: 10.11646/zootaxa.3703.1.3.

[68] Zuschin M, Ebner C. Compositional fidelity of death assemblages from a coral reef-associated tidal-flat and shallow subtidal lagoon in the northern Red Sea. Palaios. 2015;30(3): 181–191. doi: 10.2110/palo.2014.032.

Field Code Changed

Formatted: English (United States)

Field Code Changed

Supporting Information

S1 Table. **Matrix of biological traits of mollusk species.**

S2 Table. **Matrix of mollusk species per ~~samples~~sample.** The replicates are denoted as R1–3.

~~S2~~ S3 Fig. Species richness and β-diversity accumulation curves. (a) Regional species richness. (b) Regional β-diversity. (c) Habitat species richness. (d) Habitat β-diversity. (e) Local species richness Dots indicate the observed richness, shaded areas the 0.95 CIs.

~~S3. Fig. Curves of accumulation of species richness~~

S4~~.~~ Fig. Regional composition. Heat map of relative abundance (in %) of those typical species that contribute to the 70% of similarity within each gulf. Note that species were ordered by their similarity across the gulfs.

S5 Table. ~~.~~ Results of the Permutational Analysis of Variance (PERMANOVA)~~, on the multivariate structure of the death mollusks assemblages~~

Formatted: Font: 18 pt

Formatted: Font: Bold

Formatted: Font: Not Bold

Formatted: Font: Bold, Font color: Auto

Formatted: Font: Bold

Formatted: Font color: Auto

Formatted: English (United States)

Formatted: English (United States)

Formatted: Font color: Auto

Response to reviewers

Dear editor and reviewers:

Thank you very much for the sound evaluation of our paper. We appreciate your suggestions and criticisms very much, we carefully read all of them and accepted the majority. In agreement with reviewer # 2, the paper has been shortened by one third approximately. Also based on suggestions from both reviewers, we removed specific objectives 2 and 3 because of the small number of living mollusks and single deep-sea site, respectively.
Below, you can find a detailed response to each point. For easier reading, we have split paragraphs with a hyphen when specific responses are required, and our responses start with R/.

Looking forward for a positive response for this new version,

Rosely Peraza (on behalf of other authors)

Review Comments to the Author

Please use the space provided to explain your answers to the questions above. You may also include additional comments for the author, including concerns about dual publication, research ethics, or publication ethics. (Please upload your review as an attachment if it exceeds 20,000 characters)

Reviewer #1:
- This ms is well-organized and well-written ms, and addresses some important, mostly still-understudied issues in the application of molluscan dead-shell assemblages to the analysis of marine biodiversity. It includes:

R/ Thank you very much for your opinion, it means a lot to us.

- (1) a spatially-nested analysis of the diversity (several measures) and structure (not fully clear = relative abundance distribution?) of death assemblages; really nice design, and so great to see new field data from this important part of the Caribbean, on top of the general issues;

R/ Thank you. We revised all instances of the use of “community structure” in the manuscript to avoid confusion, if possible, we used a more specific term (e.g., composition).

- (2) an analysis of molluscan death assemblages as a surrogate of the combined dead+living molluscan assemblage; analogous to how benthic foram people approach field samples owing to the challenges of differentiating living and dead individuals, not clear why the authors would do this rather than a more conventional dead versus live comparison, or at least to the exclusion of a more conventional analysis (were sample sizes of living mollusks too small?); and

R/ Indeed, that was the case, living mollusks were too few, then the comparison dead vs live was highly biased because the disproportionate sample size of the two data sets. Based on your comments and the ones by reviewer # 2 about the length of the manuscript, we decided to remove the analysis of dead versus live mollusks (specific objective 2).

- (3) a test and quantification of the extent of down-slope transport of shelf-dwelling species into deep-sea, i.e. slope, sediments; wonderful to have, for comparison with the few other strong

tests of this, most notably field data from Bouchet in New Caledonia, and then a zillion anecdotal-quality observations (summarized 30y ago in Kidwell/Bosence).

R/ We agree with reviewer # 2 about that the extent of down-slope transport based on a single deep-sea site is hard to evaluate beyond doubt. Therefore, we removed the specific objective 3.

- The paper thus covers a *lot of intellectual territory and has a rather large number of figures (some could probably go into an appendix, and simply be compared using text to a smaller number of figs published in the main text). But it is well-referenced throughout, connecting the findings well with the large existing literature, both observational and theoretical.

R/ We appreciate and acknowledge your respected opinion. Following your advice, we moved those figures do not convey core information to supplementary material.

- As above, my concerns and suggestions are interspersed below among features that I really like, and were written as I initially read the ms. Overall, I think this would make an excellent paper in Plos-1 and recommend acceptance with only modest revisions, should the editor agree.
Reviewed by Susan Kidwell, thanks for the opportunity.

R/ Thanks again for your encouraging words, your work has inspired us in our studies about mollusk death assemblages.

- Methods = field collection methods were actually better than the 'usual', with authors using box cores rather than van Veens, which are the standard, although, in the end, the depth of penetration of this gear was still only ~10 cm. They thus likely missed collecting many larger-bodied, adult, and especially deeper-burrowing taxa – this is a common problem, should be mentioned either here in Methods or in the Discussion, as a 'caveat', but the authors need not be defensive about it – although it biases the sample, it is an almost universal bias, and thus permits comparison to the many many other, similarly-limited datasets (see Lees 2021 Neth J Sea Res, and Powell &Mann 2016 Cont Shelf Res for discussions). I especially admire that they acquired these samples on SCUBA, allowing for careful ad known placement… and 3 replicates per site.

R/ Agree. We added the following sentence in the Sampling section: "A potential caveat with this sampling is that it may miss deep burrowing species inhabiting below 10 cm deep".

- Description of field study areas is exemplary – these authors really describe the areas in useful ways, including extent of physical disturbance and, of course, likely human effects, drawing on earlier work (includes the admirable Table 1)… thus well-chose field areas. The 0.5mm sieve is much finer than is usual (1mm, and in some live-dead studies by geologists/paleoecologists, a 2mm or coarser sieve is used) – only a concern because this likely included juvenile individuals that can be hard to identify AND have unlikely persistence, both as living individuals (persistence to adulthood) and as dead shells (postmortem) – although I appreciate that tropical faunas can have many many 'micromollusks' that might be largely missed with a 1 mm mesh. Just something worth mentioning, as a guide to other researchers thinking of repeating this kind of analysis elsewhere.

R/ Full agreement, we chose 0.5-mm mesh size based on previous reports about the huge diversity of micro-mollusks in Cuban waters, as there are many new species described by Espinosa et al. from both studied gulfs.

- Live data have already been reported, in Armenteros et al 2018 – those results should be provided in tabular form here because the sample sizes of this living assemblage is critical to evaluating live-dead agreement.

R/ Noted. We removed objective 2 from the paper, therefore the data from Armenteros et al. are not needed any more in the paper.

- Analysis – Richness: I really like the authors' approach for taxonomic richness, strongly prefer accumulation curves rather than post-hoc rarefaction fittings, AND I like using effective numbers of species for richness – super. Phylo richness – not familiar, feels like a stretch to add this, but no objection. Functional richness – good use of Todd & Mikkelson/Bieler for coding taxa; although the N of categories is (slightly) smaller than the S of species, could still use the same metrics; but I like bringing in these FD metrics from biogeography/macroecology. Beta – many ways to do it, they should also consider (and in discussion compare their results to) the methods used in Tomasovych/Kidwell 2009-2011 (e.g., diversity transfer from alpha to beta, spatial gradient capture, richness/evenness at alpha) – a series of papers by those authors (summarized in Kidwell Tomasovych 2013) explore the effects of time-averaging on the suite of metrics used here, seems to be one part of the paleo/taph literature missed.

R/ We agree that there are many ways to approach this sort of data. This is in part the reason for a paragraph of the introduction devoted to the most relevant (in our opinion) and modern techniques for analysis of diversity. We do not wish to enlarge much the manuscript with another set of metrics and prefer to keep it as it is. Indeed, reviewer # 2 is requesting to shorten the manuscript's length.

- Evenness – would prefer to see a quantitative metric of evenness, such as the sample-size-independent Hurlbert's PIE, rather than only a graphical depiction; N singletons and doubletons is a measure of the proportion of rare taxa, which is useful/important, but not the same as dominance/evenness that focus on the proportional abundances of the top few taxa and overall shape of the cumulative richness curve. PIE is now widely used in live-dead analysis.

R/ We disagree. We prefer to use graphical devices (dominance curves) instead of reducing all the information to a single index.

- Ah—structure = simper analysis, not what I'd call structure, but rather inter-sample similarities, series of pairwise comparisons…

R/ Agree. We change the term "structure" by composition, which is clearer.

- Comparison of live-dead – using the ancient, very simple metrics of Kidwell & Bosence 1991. That's OK as long as include a table with the raw N's being compared (e.g., how similar is the N of living and dead individuals?), because those ancient authors found that the small-sample size of the living assemblages in most studies was the most important factor explaining (correlated with) the low apparent live-dead agreements (which the later modeling of Kidwell and others showed was true). But a full live-dead nowadays would explore live-dead using standard ecological measures such as (dis)similarity of paired live-dead species lists for a site/habitat/region, ditto in rank-abundance of taxa live&dead, nmds etc. [I do note a NMDS lines469; another use of the word 'structure', here meant to indicate *spatial variation in species composition of assemblages, whereas structure also used to indicate RADs… be careful, would be good to sharpen language. **I fear that the omission of such measures owes to very low abundances of living individuals, an especially large challenge in tropical settings in my experience… typically we have to process far more sediment for living than for dead to achieve statistical power (see Powell et al from the early 1980s for the first time this was recognized and acted upon; Gilad et al 2018 did the same thing, sieving many-x more volume of sediment for live than dead, in tropical shelf habitats for the same reason). If this is indeed the situation, the authors, again, do not need to be defensive but simply straightforward and 'up-front', that is

explain from the start that there are limits to what they can do analytically given extremely disparate live-dead sample sizes (and probably extremely small Ns of living in absolute terms, e.g. anything <50 inds and especially <20 inds).

R/ We removed the comparison live-dead, which corresponds to objective 2, because the very low abundance of living individuals, for most of the sites, abundance was < 20 individuals. We changed the term “structure”.

- Results – first para on general features is where the authors should immediately start differentiating their living and death assemblages – e.g., of these 7113 inds, how many dead & how many alive, ditto the S of species orders etc. If all of these numbers and others in the Results section are going to refer exclusively to the death assemblages, then this also needs to be made explicitly clear here, e.g. “All results refer only to dead-shell assemblages unless otherwise noted (e.g., section xx on dead vs total comparison).” I find it hard to proceed without knowing this basic information.

R/ We agree and follow your advice, so in the first paragraph of the results we mention that we identified 7113 dead individuals.

- Line 333 – ‘fluctuations’ typically would refer to variability in time, whereas here I think the authors mean variability among spatial replicates at a site etc collected during the same season… watch for this.

R/ Agree. We changed fluctuation × variability.

- Line 514 Table 2 – Finally! Info on N live and N dead… and indeed the disparity is super high, not shocking for a tropical shallow marine system but very troubling for live-dead comparison. My suspicion re why the authors chose to compare dead versus live+dead are confirmed. This is not a scientifically fair/appropriate test of the ability of the dead to serve as a surrogate of the usual, entirely-live-collected kind of data that biologists typically have – and moreover flips the usual disparity (usually the ‘whole’ is much larger/broader/more inclusive sampling of the target than is the subset being tests as a ‘surrogate’/proxy of i). The results are of course interesting, but mostly for enlightening readers about the challenges of live-dead analysis in such settings. I think the discussion and conclusions from this section will thus need to be very modest, very modulated. ** For the editor: The disparities are e.g. 1500 dead inds versus 5 living ones, etc, with only two sites yielding >20 individuals, with 20 being an absolute minimum N of individuals to do any kind of nmds etc.

R/ Agree. We acknowledge that the disparity between the two kind of assemblages is too large for a fair comparison, and most of the sites has < 20 alive individuals. Thus, we removed from the paper the objective 2 about comparison live-death, that necessarily implies to change the title. However, we moved the information about the abundance and observed richness of dead individuals to the table 1.

- In this table 2, I’m most intrigued by the (to me) large number of taxa found exclusively alive, i.e. “alive-only” – I would not expect this given the huge disparity in sample size, i.e. that any taxa would not be encountered among the dead. 2 explanations = (1) new, invasive taxa, populations still small, little opportunity for dead shells to accrue to measurable levels in the death assemblage [given the species, I doubt this, but authors should (in the discussion) indicate if these are all natives or otherwise commonly encountered already by others in Cuba; or (2) taphonomic bias, i.e. these are all taxa with truly exceptionally low preservation potential, e.g. from their especially small/thin-shelled bodies, and/or super-rarity, and/or high-

organic aragonitic shell types, and/or life-habit (e.g. I find that commensals have very low preservation potential). From my experience, Solemya has a super-low pres pot (as do lyonsiids, pinnids, some pandoriids), and I commonly find that Bittium, Caecum, and Rissoids/small parasitic & minute herbivores are strongly underrepresented in the dead (but not actually absent) Limaria and Americardia & Codakia should definitely not fall into this category… anyway, a surprising and super-interesting result

R/ We removed this part of the paper, although we added the abundance and number of observed species of dead mollusks in the table 1, as basic numbers for diversity assessment and sample size information.

- 523 contribution of shelf species to deep-sea death assemblages – wonderful, super useful observations. It would be super-helpful to provide the typical or maximum known body sizes of these taxa found off shelf, and/or, even better, the typical or max body sizes observed among thte individuals actually collected. Would make a more powerful discussion/conclusions

R/ These observations are valuable, but as indicated by reviewer # 2, the occurrence of a single deep-sea site (with only data of presence/absence) is a main weakness of this part. We preferred to remove objective 3 from the paper.

- Table 3 this section – Please provide info at the bottom of each column on the total N of the death assemblage so that readers can get full value from these prop abundance values. E.g. does 100% (“1”) mean 10 out of 10, 100 out of 100 inds, or 1 out of 1? The data on raw N of dead shells from a tropical slope would be valuable in itself.
  =Interesting from a taphonomic perspective that all are gastropod species w one exception… because epifaunal, which is long thought to increase the potential for postmortem transport (because they die above the sediment-water interface—idea probably in KidwellBosence)? Some info on life habit, not just gastro-bivalve class membership, would be nice to add to this table, or at least to bring into discussion.

R/ We removed table 3 from the paper.

Discussion –

- 549 hyper diverse, may never reach an asymptote – probably true, although still not a popular idea, but that’s because most people have never encountered the tropics in a first-hand way. Thus I am glad to see you say this, is a perfectly good description of your curves!

R/ Thanks!

- Regional scale – agree w your interpretation
  579 – again, people generally don’t like to talk about high productivity and coral reefs, or the role of upwelling, but am sure this is a factor in a lot of places and here you have independent evidence of upwelling to promote it; nice regional insight

R/ Thanks again!

- 614 – yes Kidwell/Bosence for the even-ing effects of time-averaging (also see the older Peterson paper they cite), but there’s more explicit, rigorous testing of this effect in younger literature, e.g. Olszewski/Kidwell 2007, var Tomasovych/Kidwell (it’s also in SK/ATomas 2013).

R/ We removed this part of the manuscript.

- 620 habitat – by the way, when I first saw ‘coral reef’ in the abstract, I assumed this would be an analysis of mollusks in the reef framework itself (with all of those huge challenges, e.g. see many nice papers by Zuschin). So I was happy/relieved to see that the authors actually meant the sandy seabeds between reef tongues, i.e. the grooves… be sure that this is clear in the abstract and intro, and then again remind reader in the discussion. Lots of potential for shells washed in from adjacent hard substrata/reef framework, but also plenty of taxa that live (and die) in the sands themselves.

R/ Agree. We made clear in the introduction that we are analyzing shells from sedimentary environments within each habitat type. Indeed, we change coral reef × reef sand across the manuscript to make clear that we deal here with sediment assemblages (and no hard-bottom assemblages).

- 640ll – yes, transport *can be important, but commonly less that folks worry about it being; thanks for having read the SK/Bosence paper carefully enough to realize that we concluded it was a relatively minor issue in most level-bottom subtidal settings (ditto even on steep shelves per SK 2008, right!).

R/ Agree.

- 657 – indeed, it *is interesting that susp feeders are as unimportant as they are in these sandy inter-reef areas ◊oh, that’s a term you could use, “inter-reef sand” rather than “coral reef”

R/ Agree. We changed the term for “reef sands”.

- To 672 – I really love all the detailed info and thinking about the natural history of your settings, it’s a usefully thoughtful discussion.

R/ Thank you very much.

- 687 – evenness curves – OK, not crazy about this approach.

R/ Dominance curves are a powerful and widely used technique to detect environmental disturbance and grossly inform about how many species are dominant, moderately dominant, and rare in the assemblages. Indeed, these curves indicated in our study the two sites subjected to local impact.

- 693 – ‘substantial inter-replicate variability, i.e. small-scale spatial heterogeneity – I find this, too, in tropical shelves… the world is patchier than we imagine, even with time-averaging. Am glad that the authors are being ‘true’, i.e. fully honest about their findings, are not trying to smooth it over.

R/ Thanks.

- Tomaso et al 2023 – no need to be defensive, this is an ‘ideal’ work plan, very difficult and expensive to achieve! BTW Adam and I try very hard to get other workers to appreciate ‘time averaging’ as a scaling process, which operates post-mortem, but NOT as a taphonomic process the way people typically mean that. T-avg does not ‘bias’ the record, but rather simply averages the input of shells over time, with a series of predictable effects (as summarized SK/AT 2013). It provides a window of opportunity for taph processes such as transport, fragmentation, bioerosion, etc to genuinely bias the composition of that time-averaged assemblage. But time averaging simply means we’re sampling bio over a period of time longer than the typical snap-shot of a biological survey – if one were to sum many live-collections over time, the effects onn richness, evenness would be the same, *unless one biased that assemblage by e.g. removing

many of the fragile-shelled species. Sorry to be this pushy, but the authors are careful thinkers and ambitious, so it would be great to have the time-averaging vs taphonomic-bias language sharpened in this ms.

R/ We agree with this approach considering time-averaging as a process through the time with predictable outcomes (e.g., increase the richness) instead of a bias. We added a sentence in the introduction to shift away the approach from bias to a time-integrated process. Also, we created a final section in the discussion to discuss the effects of time averaging on our diversity outcomes.

- 710—ah, Table 2 discussion! Ok, I see your very good discussion, thanks.

R/ Thanks.

- 737 – delighted to see this thoughtful discussion of time averaging, AND that the authors proceed to think-through their local situation given the 210Pb data at hand. Logical, fine job.

R/ We relabeled this part as a subsection in the discussion. Thanks for the encouraging opinion!!

- 757 – and dead-only treatment, good. I think they're completely right… although Kidwell/Bosence is really old, we hit upon a fundamental truth

R/ We removed this part since our evidence is rather weak given the small number of alive individuals we could analyze.

Shelf-deep-sea spp –

- I wondered about changing "deep-sea" to "slope", throughout the ms, but 1500m is a really deep part of a slope, so just leave it alone. Nice discussion.

R/ Thanks. Unfortunately, we had only a single deep-sea site, and we do not have enough evidence to support any of the three ad hoc explanations. In face of such uncertainty, we prefer to remove the analysis of shell transport between shelf and deep sea.

- Congratulations on a really nice piece of work – a *lot of fieldwork, a *lot of lab work to pick/sort/count these dead individuals down to such a small body size… and then looking at so many metrics including some still-novel ones for live-dead analysis, e.g. FD and Phylo-D.

R/ Thank you very much for your wise advices and careful revision.

With best wishes, SK

Reviewer #2:

- The manuscript " Diversity patterns of mollusk death assemblages in coral reefs and seagrass meadows and a comparison with living assemblages." authored by Peraza-Escarrá and collaborators evaluated the diversity patterns of mollusks assemblages in two gulfs and two different habitats. The main questions of the study are to (1) investigate the taxonomic, phylogenetic, and functional diversities of mollusk death assemblages at regional and local scales in 19 coral reefs and seagrass meadows; (2) evaluate death assemblages as surrogates of the whole mollusk assemblages (alive + dead); and (3) explore the downslope transport of shelf species to the deep sea. The manuscript is relatively well-written but excessively lengthy. The topic is very interesting to the scientific community, but some issues should be addressed, and missing information should be included before publication.

R/ Thanks to the reviewer for the careful revision of our paper. We will check point by point your recommendations and criticisms.

- My main concerns are about how the manuscript is organized, it gives the impression of being organized more like a chapter of a Ph.D. thesis than a scientific article, including too many details often irrelevant to the manuscript's fundamental topic. The main goal is confusing since the title highlights the "mollusk death assemblages in coral reefs and seagrass meadows and a comparison with living assemblages" and there is no specific development of this idea/topic. For example, different analyses with alive and death assemblages separately could be interesting for differentiating them and could be interesting in terms of current ecological differences between seagrass meadows and coral reef mollusks.

R/ We agree with the reviewer's criticism regarding the length of the manuscript. We also have in account the remarks by reviewer # 1 about the small sample size of the living mollusk database. Therefore, we removed the specific objectives 2 and 3, that contribute together about 1/3 to the whole paper and are based on rather limited evidence due to small sample size (objective 2, living assemblages) and reduced spatial coverage with single deep-sea site (objective 3, shelf-slope transport of shells). We note that given the large amount of information contained in objective 1, the paper still has 2/3 of the original length/content.

- Finally, for the proposed experimental design is also necessary to provide more specific details that are not yet included.

R/ We provided more details in this section.

- The paper in its current form needs major revision before publication. Below I provide some suggestions to improve the manuscript.

R/ Thanks. We addressed all the suggestions.

Specific comments

- The authors should carefully review and choose the article's topic and then select the important and relevant information on that topic (seagrass meadows vs coral reef?), discarding the rest. In its current form, I consider the manuscript to be too lengthy, containing a wealth of information without a clear guiding thread. On the other hand, it appears to be a mix of data and interpretations in which, at times, it is difficult to find a common thread. Once the authors focus on the objective of what they want to convey in the article, they should direct the discussion to that topic. It's worth noting that the bulk of the work has already been done, as this should only involve a readjustment and reorganization of content.

R/ We agree. As stated above, we removed objectives 2 and 3 and focused on the diversity patterns of dead assemblages.

- A thorough review of the English is necessary to correct mistakes.

R/ We revised the English usage.

- Abstract. Authors should integrate better the sentences, sometimes seems to be a succession of sentences without links between them.

R/ Agree. We revised the abstract.

Introduction

- The authors should rearrange the introduction. I consider it very important to include information about coral reefs and seagrass meadows in the ocean and specifically the mollusk assemblages in those habitats, and why is very important to study them.

R/ Agree. We added a couple of sentences about the two habitats in the introduction, including a statement about the specific biotope sampled (sediments).

- Authors should reorganize the introduction from the most general to the most specific.

R/ Agree. Introduction covers some topics that necessarily must be started de novo, for instance, dead assemblages, habitat, diversity, and Cuba (regional and local settings). However, we improved the connection between paragraphs, keeping the order from general to specific.

Material and Methods

- Why isn't there the same number of replicates in all cases? Because in the case of GB, we have two seagrass sites and two coral reef sites, but in the case of GG, there are 3 and 3? Why wasn't the same number of replicates (n=3) maintained in all cases? Justify it.

R/ The number of replicates (box cores) was n = 3 for eight out of 11 sites, only in three sites we could not collect 3 replicates because diving time was not enough for it. We note that the number of studied sites was different because of limits imposed by the distance of navigation, and amount of material to be processed/identified. We believe that even with this unbalanced setting, our data is valuable and robust.

- Give more details of the deep-sea sampling.

R/ As suggested by the reviewer, this part of the manuscript was removed.

- Is a single slope replicate sufficient to address the differences between the communities of the platform and the slope? Shouldn't the study be balanced? Justify it because this fact could compromise the entire comparison.

R/ Agree. The evidence here is rather limited due to a single deep-sea site being studied. Therefore, we removed this part from the manuscript.

- Authors should also consider to conduct multivariate analyses such as PERMANOVA that assess the entire community and separately for living and dead mollusk assemblages.

R/ The disparate sample size between dead and living assemblages is a main shortage for a fair comparison. Therefore, we removed the section of the manuscript related to the living assemblages. However, we made a PERMANOVA for complementing the results of the assemblage structure because in this case we can use the replicates in the statistical analysis.

- It should be considered by the authors to give more details of the experimental design used, specifying the fixed factors, random factors, nested and orthogonal factors.

R/ Agree. More information was added to the manuscript.

Results

- At times, it is stated that there are significant differences between certain factors. What statistical tests have been conducted to assert the existence of such differences?

R/ …In the manuscript, subsection of data analysis we stated: "In general, the criterion for testing differences between groups was the overlapping of the 0.95 CIs calculated on basis of

permutations". We note here that for the analysis of richness, the most appropriate approach is to sum all the replicates of the sample, it means that no replication exists for making a traditional test such as analysis of variance. In this case, the way to generate a statistical device for the comparison is the calculation of confidence intervals (CIs) based on permutations and to use, as criterion of significance, the overlapping (or not) of the 0.95 CIs.

- The title refers to the comparison of living mollusks with the dead ones, but this comparison is not explicitly addressed except in a small section discussing percentages. Furthermore, throughout the manuscript, there is no mention of a separate matrix that differentiates these two distinct communities

R/ We agree. We removed the part of the comparison between living and dead, and modified the title in consequence.

- Some parts of the results are redundant and some are unnecessary (depending on the topic of the manuscript); for instance, the information provided in Figures 4 and 7 is essentially the same. It would make more biological sense to treat it separately by habitats as it appears in Fig 7 (seagrass and coral reefs) according to the indicated in the title of the work, rather than jointly in GB and GG. The authors should review this idea and provide only the necessary results for the study's topic.

R/ We disagree. The spatial scale is a very important issue in diversity studies; therefore, we consider it very important to describe the regional (i.e., between gulfs) and local (i.e., between sites) patterns.

- Perhaps it's a good idea to analyze the data in a more general manner without delving into details of differences between replicates and specific gulfs. If the goal is to contribute to the broader scientific community, it might be more important to focus on observable differences in mollusk assemblages between habitats (seagrass vs. coral reef). This approach could involve explaining differences in species number, abundance, and functional traits rather than detailing local differences between the two. With this shift, the almost exclusive local focus of the study can be broadened to a much more global interest

R/ We disagree. As stated above, the comparison at two spatial scales is relevant to current biodiversity research. The two large spatial units compared here (GG and GB) belong to two biomes of paramount importance in the tropical Atlantic Ocean: Gulf of Mexico and Caribbean Sea, respectively. In our opinion, there is no way that the comparison between these units be local or not interesting. This sort of comparison is the foundation of biogeography, and we believe they must be analyzed in the paper as is. In addition, to analyze diversity at local scale allows to disentangle the role of local processes on the difference at level of habitat. Indeed, the nested analysis indicates that the local differences are very important to explain the large variability within the same habitat.

Discussion

- Considering the aforementioned points, it might be advantageous to reframe the discussion. Instead of delving into intricate details of differences between replicas and specific gulf regions, a more effective approach could be to analyze the data in a broader context. Focusing on the overarching differences in mollusk assemblages between habitats, specifically comparing seagrass and coral reef environments, would align better with a global scientific community. This approach would involve elucidating variations in species number, abundance, and functional traits, steering away from intricate local disparities. By adopting this perspective, the

study's interest could transition from predominantly local to a more globally significant contribution

R/ This comment is in the same spirit as the previous one and we disagree again. We do not see why global community would be interested to compare between seagrass and coral reefs, and not equally interested to compare spatial units between Gulf of Mexico and Caribbean Sea. Also, the role of local processes is very important to interpret diversity variation, based on the suggested point of view, no study using analysis between sites should be published. We think that the articulation in a nested design of spatial units (gulfs, sites) and habitats is a methodological strength of our study.

Figures and Tables

- The authors should integrate the Figures and Tables in the manuscript as closely as possible to the reference in the text to facilitate the reading of the manuscript.

R/ We followed the journal's guideline, that is only figure captions are within the text, and figures at the end.

- The authors should keep only relevant figures and tables, taking in mind that this is a scientific paper rather than a chapter where considering and including all the possible information is necessary and mandatory.

R/ Agree. After the reorganization of the paper, the number of figures was reduced from 11 to 10, and the tables from 3 to 1. Two figures were simplified, and part of the information was sent to supplementary material.
