# Supplementary material for: Taxonomic, phylogenetic, and functional diversity of mollusk death assemblages in coral reef and seagrass sediments from two shallow gulfs in Western Cuban Archipelago
Source: PLoS One. 2024 May 14;19(5):e0303539. doi: 10.1371/journal.pone.0303539 (PMC11093297; doi:10.1371/journal.pone.0303539)
Supplement: Attachment — Submitted filename: Response_to_reviewers.docx [file pone.0303539.s006.docx]

**Response to reviewers**

Dear editor and reviewers:

Thank you very much for the sound evaluation of our paper. We appreciate your suggestions and criticisms very much, we carefully read all of them and accepted the majority. In agreement with reviewer # 2, the paper has been shortened by one third approximately. Also based on suggestions from both reviewers, we removed specific objectives 2 and 3 because of the small number of living mollusks and single deep-sea site, respectively.

Below, you can find a detailed response to each point. For easier reading, we have split paragraphs with a hyphen when specific responses are required, and our responses start with R/.

Looking forward for a positive response for this new version,

Rosely Peraza (on behalf of other authors)

Review Comments to the Author

Please use the space provided to explain your answers to the questions above. You may also include additional comments for the author, including concerns about dual publication, research ethics, or publication ethics. (Please upload your review as an attachment if it exceeds 20,000 characters)

**Reviewer #1:**

- This ms is well-organized and well-written ms, and addresses some important, mostly still-understudied issues in the application of molluscan dead-shell assemblages to the analysis of marine biodiversity. It includes:

R/ Thank you very much for your opinion, it means a lot to us.

- (1) a spatially-nested analysis of the diversity (several measures) and structure (not fully clear = relative abundance distribution?) of death assemblages; really nice design, and so great to see new field data from this important part of the Caribbean, on top of the general issues;

R/ Thank you. We revised all instances of the use of “community structure” in the manuscript to avoid confusion, if possible, we used a more specific term (e.g., composition).

- (2) an analysis of molluscan death assemblages as a surrogate of the combined dead+living molluscan assemblage; analogous to how benthic foram people approach field samples owing to the challenges of differentiating living and dead individuals, not clear why the authors would do this rather than a more conventional dead versus live comparison, or at least to the exclusion of a more conventional analysis (were sample sizes of living mollusks too small?); and

R/ Indeed, that was the case, living mollusks were too few, then the comparison dead vs live was highly biased because the disproportionate sample size of the two data sets. Based on your comments and the ones by reviewer # 2 about the length of the manuscript, we decided to remove the analysis of dead versus live mollusks (specific objective 2).

- (3) a test and quantification of the extent of down-slope transport of shelf-dwelling species into deep-sea, i.e. slope, sediments; wonderful to have, for comparison with the few other strong tests of this, most notably field data from Bouchet in New Caledonia, and then a zillion anecdotal-quality observations (summarized 30y ago in Kidwell/Bosence).

R/ We agree with reviewer # 2 about that the extent of down-slope transport based on a single deep-sea site is hard to evaluate beyond doubt. Therefore, we removed the specific objective 3.

- The paper thus covers a *lot of intellectual territory and has a rather large number of figures (some could probably go into an appendix, and simply be compared using text to a smaller number of figs published in the main text). But it is well-referenced throughout, connecting the findings well with the large existing literature, both observational and theoretical.

R/ We appreciate and acknowledge your respected opinion. Following your advice, we moved those figures do not convey core information to supplementary material.

- As above, my concerns and suggestions are interspersed below among features that I really like, and were written as I initially read the ms. Overall, I think this would make an excellent paper in Plos-1 and recommend acceptance with only modest revisions, should the editor agree.
Reviewed by Susan Kidwell, thanks for the opportunity.

R/ Thanks again for your encouraging words, your work has inspired us in our studies about mollusk death assemblages.

- Methods = field collection methods were actually better than the ‘usual’, with authors using box cores rather than van Veens, which are the standard, although, in the end, the depth of penetration of this gear was still only ~10 cm. They thus likely missed collecting many larger-bodied, adult, and especially deeper-burrowing taxa – this is a common problem, should be mentioned either here in Methods or in the Discussion, as a ‘caveat’, but the authors need not be defensive about it – although it biases the sample, it is an almost universal bias, and thus permits comparison to the many many other, similarly-limited datasets (see Lees 2021 Neth J Sea Res, and Powell &Mann 2016 Cont Shelf Res for discussions). I especially admire that they acquired these samples on SCUBA, allowing for careful ad known placement… and 3 replicates per site.

R/ Agree. We added the following sentence in the Sampling section: “A potential caveat with this sampling is that it may miss deep burrowing species inhabiting below 10 cm deep”.

- Description of field study areas is exemplary – these authors really describe the areas in useful ways, including extent of physical disturbance and, of course, likely human effects, drawing on earlier work (includes the admirable Table 1)… thus well-chose field areas. The 0.5mm sieve is much finer than is usual (1mm, and in some live-dead studies by geologists/paleoecologists, a 2mm or coarser sieve is used) – only a concern because this likely included juvenile individuals that can be hard to identify AND have unlikely persistence, both as living individuals (persistence to adulthood) and as dead shells (postmortem) – although I appreciate that tropical faunas can have many many ‘micromollusks’ that might be largely missed with a 1 mm mesh. Just something worth mentioning, as a guide to other researchers thinking of repeating this kind of analysis elsewhere.

R/ Full agreement, we chose 0.5-mm mesh size based on previous reports about the huge diversity of micro-mollusks in Cuban waters, as there are many new species described by Espinosa et al. from both studied gulfs.

- Live data have already been reported, in Armenteros et al 2018 – those results should be provided in tabular form here because the sample sizes of this living assemblage is critical to evaluating live-dead agreement.

R/ Noted. We removed objective 2 from the paper, therefore the data from Armenteros et al. are not needed any more in the paper.

- Analysis – Richness: I really like the authors’ approach for taxonomic richness, strongly prefer accumulation curves rather than post-hoc rarefaction fittings, AND I like using effective numbers of species for richness – super. Phylo richness – not familiar, feels like a stretch to add this, but no objection. Functional richness – good use of Todd & Mikkelson/Bieler for coding taxa; although the N of categories is (slightly) smaller than the S of species, could still use the same metrics; but I like bringing in these FD metrics from biogeography/macroecology. Beta – many ways to do it, they should also consider (and in discussion compare their results to) the methods used in Tomasovych/Kidwell 2009-2011 (e.g., diversity transfer from alpha to beta, spatial gradient capture, richness/evenness at alpha) – a series of papers by those authors (summarized in Kidwell Tomasovych 2013) explore the effects of time-averaging on the suite of metrics used here, seems to be one part of the paleo/taph literature missed.

R/ We agree that there are many ways to approach this sort of data. This is in part the reason for a paragraph of the introduction devoted to the most relevant (in our opinion) and modern techniques for analysis of diversity. We do not wish to enlarge much the manuscript with another set of metrics and prefer to keep it as it is. Indeed, reviewer # 2 is requesting to shorten the manuscript’s length.

- Evenness – would prefer to see a quantitative metric of evenness, such as the sample-size-independent Hurlbert’s PIE, rather than only a graphical depiction; N singletons and doubletons is a measure of the proportion of rare taxa, which is useful/important, but not the same as dominance/evenness that focus on the proportional abundances of the top few taxa and overall shape of the cumulative richness curve. PIE is now widely used in live-dead analysis.

R/ We disagree. We prefer to use graphical devices (dominance curves) instead of reducing all the information to a single index.

- Ah—structure = simper analysis, not what I’d call structure, but rather inter-sample similarities, series of pairwise comparisons…

R/ Agree. We change the term “structure” by composition, which is clearer.

- Comparison of live-dead – using the ancient, very simple metrics of Kidwell & Bosence 1991. That’s OK as long as include a table with the raw N’s being compared (e.g., how similar is the N of living and dead individuals?), because those ancient authors found that the small-sample size of the living assemblages in most studies was the most important factor explaining (correlated with) the low apparent live-dead agreements (which the later modeling of Kidwell and others showed was true). But a full live-dead nowadays would explore live-dead using standard ecological measures such as (dis)similarity of paired live-dead species lists for a site/habitat/region, ditto in rank-abundance of taxa live&dead, nmds etc. [I do note a NMDS lines469; another use of the word ‘structure’, here meant to indicate *spatial variation in species composition of assemblages, whereas structure also used to indicate RADs… be careful, would be good to sharpen language. **I fear that the omission of such measures owes to very low abundances of living individuals, an especially large challenge in tropical settings in my experience… typically we have to process far more sediment for living than for dead to achieve statistical power (see Powell et al from the early 1980s for the first time this was recognized and acted upon; Gilad et al 2018 did the same thing, sieving many-x more volume of sediment for live than dead, in tropical shelf habitats for the same reason). If this is indeed the situation, the authors, again, do not need to be defensive but simply straightforward and ‘up-front’, that is explain from the start that there are limits to what they can do analytically given extremely disparate live-dead sample sizes (and probably extremely small Ns of living in absolute terms, e.g. anything <50 inds and especially <20 inds).

R/ We removed the comparison live-dead, which corresponds to objective 2, because the very low abundance of living individuals, for most of the sites, abundance was < 20 individuals. We changed the term “structure”.

- Results – first para on general features is where the authors should immediately start differentiating their living and death assemblages – e.g., of these 7113 inds, how many dead & how many alive, ditto the S of species orders etc. If all of these numbers and others in the Results section are going to refer exclusively to the death assemblages, then this also needs to be made explicitly clear here, e.g. “All results refer only to dead-shell assemblages unless otherwise noted (e.g., section xx on dead vs total comparison).” I find it hard to proceed without knowing this basic information.

R/ We agree and follow your advice, so in the first paragraph of the results we mention that we identified 7113 dead individuals.

- Line 333 – ‘fluctuations’ typically would refer to variability in time, whereas here I think the authors mean variability among spatial replicates at a site etc collected during the same season… watch for this.

R/ Agree. We changed fluctuation × variability.

- Line 514 Table 2 – Finally! Info on N live and N dead… and indeed the disparity is super high, not shocking for a tropical shallow marine system but very troubling for live-dead comparison. My suspicion re why the authors chose to compare dead versus live+dead are confirmed. This is not a scientifically fair/appropriate test of the ability of the dead to serve as a surrogate of the usual, entirely-live-collected kind of data that biologists typically have – and moreover flips the usual disparity (usually the ‘whole’ is much larger/broader/more inclusive sampling of the target than is the subset being tests as a ‘surrogate’/proxy of i). The results are of course interesting, but mostly for enlightening readers about the challenges of live-dead analysis in such settings. I think the discussion and conclusions from this section will thus need to be very modest, very modulated. ** For the editor: The disparities are e.g. 1500 dead inds versus 5 living ones, etc, with only two sites yielding >20 individuals, with 20 being an absolute minimum N of individuals to do any kind of nmds etc.

R/ Agree. We acknowledge that the disparity between the two kind of assemblages is too large for a fair comparison, and most of the sites has < 20 alive individuals. Thus, we removed from the paper the objective 2 about comparison live-death, that necessarily implies to change the title. However, we moved the information about the abundance and observed richness of dead individuals to the table 1.

- In this table 2, I’m most intrigued by the (to me) large number of taxa found exclusively alive, i.e. “alive-only” – I would not expect this given the huge disparity in sample size, i.e. that any taxa would not be encountered among the dead. 2 explanations = (1) new, invasive taxa, populations still small, little opportunity for dead shells to accrue to measurable levels in the death assemblage [given the species, I doubt this, but authors should (in the discussion) indicate if these are all natives or otherwise commonly encountered already by others in Cuba; or (2) taphonomic bias, i.e. these are all taxa with truly exceptionally low preservation potential, e.g. from their especially small/thin-shelled bodies, and/or super-rarity, and/or high-organic aragonitic shell types, and/or life-habit (e.g. I find that commensals have very low preservation potential). From my experience, Solemya has a super-low pres pot (as do lyonsiids, pinnids, some pandoriids), and I commonly find that Bittium, Caecum, and Rissoids/small parasitic & minute herbivores are strongly underrepresented in the dead (but not actually absent) Limaria and Americardia & Codakia should definitely not fall into this category… anyway, a surprising and super-interesting result

R/ We removed this part of the paper, although we added the abundance and number of observed species of dead mollusks in the table 1, as basic numbers for diversity assessment and sample size information.

- 523 contribution of shelf species to deep-sea death assemblages – wonderful, super useful observations. It would be super-helpful to provide the typical or maximum known body sizes of these taxa found off shelf, and/or, even better, the typical or max body sizes observed among thte individuals actually collected. Would make a more powerful discussion/conclusions

R/ These observations are valuable, but as indicated by reviewer # 2, the occurrence of a single deep-sea site (with only data of presence/absence) is a main weakness of this part. We preferred to remove objective 3 from the paper.

- Table 3 this section – Please provide info at the bottom of each column on the total N of the death assemblage so that readers can get full value from these prop abundance values. E.g. does 100% (“1”) mean 10 out of 10, 100 out of 100 inds, or 1 out of 1? The data on raw N of dead shells from a tropical slope would be valuable in itself.
  =Interesting from a taphonomic perspective that all are gastropod species w one exception… because epifaunal, which is long thought to increase the potential for postmortem transport (because they die above the sediment-water interface—idea probably in KidwellBosence)? Some info on life habit, not just gastro-bivalve class membership, would be nice to add to this table, or at least to bring into discussion.

R/ We removed table 3 from the paper.

Discussion –

- 549 hyper diverse, may never reach an asymptote – probably true, although still not a popular idea, but that’s because most people have never encountered the tropics in a first-hand way. Thus I am glad to see you say this, is a perfectly good description of your curves!

R/ Thanks!

- Regional scale – agree w your interpretation
  579 – again, people generally don’t like to talk about high productivity and coral reefs, or the role of upwelling, but am sure this is a factor in a lot of places and here you have independent evidence of upwelling to promote it; nice regional insight

R/ Thanks again!

- 614 – yes Kidwell/Bosence for the even-ing effects of time-averaging (also see the older Peterson paper they cite), but there’s more explicit, rigorous testing of this effect in younger literature, e.g. Olszewski/Kidwell 2007, var Tomasovych/Kidwell (it’s also in SK/ATomas 2013).

R/ We removed this part of the manuscript.

- 620 habitat – by the way, when I first saw ‘coral reef’ in the abstract, I assumed this would be an analysis of mollusks in the reef framework itself (with all of those huge challenges, e.g. see many nice papers by Zuschin). So I was happy/relieved to see that the authors actually meant the sandy seabeds between reef tongues, i.e. the grooves… be sure that this is clear in the abstract and intro, and then again remind reader in the discussion. Lots of potential for shells washed in from adjacent hard substrata/reef framework, but also plenty of taxa that live (and die) in the sands themselves.

R/ Agree. We made clear in the introduction that we are analyzing shells from sedimentary environments within each habitat type. Indeed, we change coral reef × reef sand across the manuscript to make clear that we deal here with sediment assemblages (and no hard-bottom assemblages).

- 640ll – yes, transport *can be important, but commonly less that folks worry about it being; thanks for having read the SK/Bosence paper carefully enough to realize that we concluded it was a relatively minor issue in most level-bottom subtidal settings (ditto even on steep shelves per SK 2008, right!).

R/ Agree.

- 657 – indeed, it *is interesting that susp feeders are as unimportant as they are in these sandy inter-reef areas ◊oh, that’s a term you could use, “inter-reef sand” rather than “coral reef”

R/ Agree. We changed the term for “reef sands”.

- To 672 – I really love all the detailed info and thinking about the natural history of your settings, it’s a usefully thoughtful discussion.

R/ Thank you very much.

- 687 – evenness curves – OK, not crazy about this approach.

R/ Dominance curves are a powerful and widely used technique to detect environmental disturbance and grossly inform about how many species are dominant, moderately dominant, and rare in the assemblages. Indeed, these curves indicated in our study the two sites subjected to local impact.

- 693 – ‘substantial inter-replicate variability, i.e. small-scale spatial heterogeneity – I find this, too, in tropical shelves… the world is patchier than we imagine, even with time-averaging. Am glad that the authors are being ‘true’, i.e. fully honest about their findings, are not trying to smooth it over.

R/ Thanks.

- Tomaso et al 2023 – no need to be defensive, this is an ‘ideal’ work plan, very difficult and expensive to achieve! BTW Adam and I try very hard to get other workers to appreciate ‘time averaging’ as a scaling process, which operates post-mortem, but NOT as a taphonomic process the way people typically mean that. T-avg does not ‘bias’ the record, but rather simply averages the input of shells over time, with a series of predictable effects (as summarized SK/AT 2013). It provides a window of opportunity for taph processes such as transport, fragmentation, bioerosion, etc to genuinely bias the composition of that time-averaged assemblage. But time averaging simply means we’re sampling bio over a period of time longer than the typical snap-shot of a biological survey – if one were to sum many live-collections over time, the effects onn richness, evenness would be the same, *unless one biased that assemblage by e.g. removing many of the fragile-shelled species. Sorry to be this pushy, but the authors are careful thinkers and ambitious, so it would be great to have the time-averaging vs taphonomic-bias language sharpened in this ms.

R/ We agree with this approach considering time-averaging as a process through the time with predictable outcomes (e.g., increase the richness) instead of a bias. We added a sentence in the introduction to shift away the approach from bias to a time-integrated process. Also, we created a final section in the discussion to discuss the effects of time averaging on our diversity outcomes.

- 710—ah, Table 2 discussion! Ok, I see your very good discussion, thanks.

R/ Thanks.

- 737 – delighted to see this thoughtful discussion of time averaging, AND that the authors proceed to think-through their local situation given the 210Pb data at hand. Logical, fine job.

R/ We relabeled this part as a subsection in the discussion. Thanks for the encouraging opinion!!

- 757 – and dead-only treatment, good. I think they’re completely right… although Kidwell/Bosence is really old, we hit upon a fundamental truth

R/ We removed this part since our evidence is rather weak given the small number of alive individuals we could analyze.

Shelf-deep-sea spp –

- I wondered about changing “deep-sea” to “slope”, throughout the ms, but 1500m is a really deep part of a slope, so just leave it alone. Nice discussion.

R/ Thanks. Unfortunately, we had only a single deep-sea site, and we do not have enough evidence to support any of the three ad hoc explanations. In face of such uncertainty, we prefer to remove the analysis of shell transport between shelf and deep sea.

- Congratulations on a really nice piece of work – a *lot of fieldwork, a *lot of lab work to pick/sort/count these dead individuals down to such a small body size… and then looking at so many metrics including some still-novel ones for live-dead analysis, e.g. FD and Phylo-D.

R/ Thank you very much for your wise advices and careful revision.

With best wishes, SK

**Reviewer #2**:

- The manuscript " Diversity patterns of mollusk death assemblages in coral reefs and seagrass meadows and a comparison with living assemblages." authored by Peraza-Escarrá and collaborators evaluated the diversity patterns of mollusks assemblages in two gulfs and two different habitats. The main questions of the study are to (1) investigate the taxonomic, phylogenetic, and functional diversities of mollusk death assemblages at regional and local scales in 19 coral reefs and seagrass meadows; (2) evaluate death assemblages as surrogates of the whole mollusk assemblages (alive + dead); and (3) explore the downslope transport of shelf species to the deep sea. The manuscript is relatively well-written but excessively lengthy. The topic is very interesting to the scientific community, but some issues should be addressed, and missing information should be included before publication.

R/ Thanks to the reviewer for the careful revision of our paper. We will check point by point your recommendations and criticisms.

- My main concerns are about how the manuscript is organized, it gives the impression of being organized more like a chapter of a Ph.D. thesis than a scientific article, including too many details often irrelevant to the manuscript's fundamental topic. The main goal is confusing since the title highlights the “mollusk death assemblages in coral reefs and seagrass meadows and a comparison with living assemblages” and there is no specific development of this idea/topic. For example, different analyses with alive and death assemblages separately could be interesting for differentiating them and could be interesting in terms of current ecological differences between seagrass meadows and coral reef mollusks.

R/ We agree with the reviewer’s criticism regarding the length of the manuscript. We also have in account the remarks by reviewer # 1 about the small sample size of the living mollusk database. Therefore, we removed the specific objectives 2 and 3, that contribute together about 1/3 to the whole paper and are based on rather limited evidence due to small sample size (objective 2, living assemblages) and reduced spatial coverage with single deep-sea site (objective 3, shelf-slope transport of shells). We note that given the large amount of information contained in objective 1, the paper still has 2/3 of the original length/content.

- Finally, for the proposed experimental design is also necessary to provide more specific details that are not yet included.

R/ We provided more details in this section.

- The paper in its current form needs major revision before publication. Below I provide some suggestions to improve the manuscript.

R/ Thanks. We addressed all the suggestions.

Specific comments

- The authors should carefully review and choose the article's topic and then select the important and relevant information on that topic (seagrass meadows vs coral reef?), discarding the rest. In its current form, I consider the manuscript to be too lengthy, containing a wealth of information without a clear guiding thread. On the other hand, it appears to be a mix of data and interpretations in which, at times, it is difficult to find a common thread. Once the authors focus on the objective of what they want to convey in the article, they should direct the discussion to that topic. It's worth noting that the bulk of the work has already been done, as this should only involve a readjustment and reorganization of content.

R/ We agree. As stated above, we removed objectives 2 and 3 and focused on the diversity patterns of dead assemblages.

- A thorough review of the English is necessary to correct mistakes.

R/ We revised the English usage.

- Abstract. Authors should integrate better the sentences, sometimes seems to be a succession of sentences without links between them.

R/ Agree. We revised the abstract.

Introduction

- The authors should rearrange the introduction. I consider it very important to include information about coral reefs and seagrass meadows in the ocean and specifically the mollusk assemblages in those habitats, and why is very important to study them.

R/ Agree. We added a couple of sentences about the two habitats in the introduction, including a statement about the specific biotope sampled (sediments).

- Authors should reorganize the introduction from the most general to the most specific.

R/ Agree. Introduction covers some topics that necessarily must be started de novo, for instance, dead assemblages, habitat, diversity, and Cuba (regional and local settings). However, we improved the connection between paragraphs, keeping the order from general to specific.

Material and Methods

- Why isn't there the same number of replicates in all cases? Because in the case of GB, we have two seagrass sites and two coral reef sites, but in the case of GG, there are 3 and 3? Why wasn't the same number of replicates (n=3) maintained in all cases? Justify it.

R/ The number of replicates (box cores) was n = 3 for eight out of 11 sites, only in three sites we could not collect 3 replicates because diving time was not enough for it. We note that the number of studied sites was different because of limits imposed by the distance of navigation, and amount of material to be processed/identified. We believe that even with this unbalanced setting, our data is valuable and robust.

- Give more details of the deep-sea sampling.

R/ As suggested by the reviewer, this part of the manuscript was removed.

- Is a single slope replicate sufficient to address the differences between the communities of the platform and the slope? Shouldn't the study be balanced? Justify it because this fact could compromise the entire comparison.

R/ Agree. The evidence here is rather limited due to a single deep-sea site being studied. Therefore, we removed this part from the manuscript.

- Authors should also consider to conduct multivariate analyses such as PERMANOVA that assess the entire community and separately for living and dead mollusk assemblages.

R/ The disparate sample size between dead and living assemblages is a main shortage for a fair comparison. Therefore, we removed the section of the manuscript related to the living assemblages. However, we made a PERMANOVA for complementing the results of the assemblage structure because in this case we can use the replicates in the statistical analysis.

- It should be considered by the authors to give more details of the experimental design used, specifying the fixed factors, random factors, nested and orthogonal factors.

R/ Agree. More information was added to the manuscript.

Results

- At times, it is stated that there are significant differences between certain factors. What statistical tests have been conducted to assert the existence of such differences?

R/ …In the manuscript, subsection of data analysis we stated: “In general, the criterion for testing differences between groups was the overlapping of the 0.95 CIs calculated on basis of permutations”. We note here that for the analysis of richness, the most appropriate approach is to sum all the replicates of the sample, it means that no replication exists for making a traditional test such as analysis of variance. In this case, the way to generate a statistical device for the comparison is the calculation of confidence intervals (CIs) based on permutations and to use, as criterion of significance, the overlapping (or not) of the 0.95 CIs.

- The title refers to the comparison of living mollusks with the dead ones, but this comparison is not explicitly addressed except in a small section discussing percentages. Furthermore, throughout the manuscript, there is no mention of a separate matrix that differentiates these two distinct communities

R/ We agree. We removed the part of the comparison between living and dead, and modified the title in consequence.

- Some parts of the results are redundant and some are unnecessary (depending on the topic of the manuscript); for instance, the information provided in Figures 4 and 7 is essentially the same. It would make more biological sense to treat it separately by habitats as it appears in Fig 7 (seagrass and coral reefs) according to the indicated in the title of the work, rather than jointly in GB and GG. The authors should review this idea and provide only the necessary results for the study's topic.

R/ We disagree. The spatial scale is a very important issue in diversity studies; therefore, we consider it very important to describe the regional (i.e., between gulfs) and local (i.e., between sites) patterns.

- Perhaps it's a good idea to analyze the data in a more general manner without delving into details of differences between replicates and specific gulfs. If the goal is to contribute to the broader scientific community, it might be more important to focus on observable differences in mollusk assemblages between habitats (seagrass vs. coral reef). This approach could involve explaining differences in species number, abundance, and functional traits rather than detailing local differences between the two. With this shift, the almost exclusive local focus of the study can be broadened to a much more global interest

R/ We disagree. As stated above, the comparison at two spatial scales is relevant to current biodiversity research. The two large spatial units compared here (GG and GB) belong to two biomes of paramount importance in the tropical Atlantic Ocean: Gulf of Mexico and Caribbean Sea, respectively. In our opinion, there is no way that the comparison between these units be local or not interesting. This sort of comparison is the foundation of biogeography, and we believe they must be analyzed in the paper as is. In addition, to analyze diversity at local scale allows to disentangle the role of local processes on the difference at level of habitat. Indeed, the nested analysis indicates that the local differences are very important to explain the large variability within the same habitat.

Discussion

- Considering the aforementioned points, it might be advantageous to reframe the discussion. Instead of delving into intricate details of differences between replicas and specific gulf regions, a more effective approach could be to analyze the data in a broader context. Focusing on the overarching differences in mollusk assemblages between habitats, specifically comparing seagrass and coral reef environments, would align better with a global scientific community. This approach would involve elucidating variations in species number, abundance, and functional traits, steering away from intricate local disparities. By adopting this perspective, the study's interest could transition from predominantly local to a more globally significant contribution

R/ This comment is in the same spirit as the previous one and we disagree again. We do not see why global community would be interested to compare between seagrass and coral reefs, and not equally interested to compare spatial units between Gulf of Mexico and Caribbean Sea. Also, the role of local processes is very important to interpret diversity variation, based on the suggested point of view, no study using analysis between sites should be published. We think that the articulation in a nested design of spatial units (gulfs, sites) and habitats is a methodological strength of our study.

Figures and Tables

- The authors should integrate the Figures and Tables in the manuscript as closely as possible to the reference in the text to facilitate the reading of the manuscript.

R/ We followed the journal’s guideline, that is only figure captions are within the text, and figures at the end.

- The authors should keep only relevant figures and tables, taking in mind that this is a scientific paper rather than a chapter where considering and including all the possible information is necessary and mandatory.

R/ Agree. After the reorganization of the paper, the number of figures was reduced from 11 to 10, and the tables from 3 to 1. Two figures were simplified, and part of the information was sent to supplementary material.
