# Supplementary material for: Taxonomic, phylogenetic, and functional diversity of mollusk death assemblages in coral reef and seagrass sediments from two shallow gulfs in Western Cuban Archipelago
Source: PLoS One. 2024 May 14;19(5):e0303539. doi: 10.1371/journal.pone.0303539 (PMC11093297; doi:10.1371/journal.pone.0303539)
Supplement: Attachment — Submitted filename: Response to Reviewers.docx [file pone.0303539.s008.docx]

**Response to Reviewers**

**Response to Editor:**
Dear editor, thank you very much for guiding us during the revision process of our manuscript. As requested, we have addressed all the comments made by the Reviewer #3. We also revised the English usage and improved the readiness of some parts of the manuscript.

**Response to reviewers:**

Dear reviewers, thank you very much for taking the time to revise our manuscript, we appreciate your comments and criticisms very much. We have addressed all your comments. Please, see our responses below.

**Reviewer #2:**

The manuscript titled "Taxonomic, phylogenetic, and functional diversity of mollusk death assemblages in coral reef and seagrass sediments from two shallow gulfs in the western Cuban archipelago," authored by Peraza-Escarrá and collaborators, evaluates diversity patterns of mollusk assemblages in two gulfs and across two different habitats. This topic is of significant interest to the scientific community. Following the authors' thorough addressing of all raised issues from the previous review, as well as the resolution of my concerns, the manuscript has undergone substantial improvement. In my assessment, it has now reached the necessary standard for acceptance for publication.

R/ Thank you very much for your valuable comments that significantly improved our paper.

**Reviewer #3:**

This manuscript addresses the diversity (taxonomic, phylogenetic and functional) of mollusc death assemblages in two different habitats in the Western Cuban Archipelago.

The topic is interesting, worth of being published and the manuscript seems to have been well improved after the first round of review. The manuscript is well organized and the analyses are appropriate for the goals of this work. I propose some suggestions aimed at improving the clarity and importance of the work, most of my comments are in the annotated text. Some additional points are below.

R/ Thank you very much for your valuable comments that significantly improved our paper. For the comments below, we provide a response point by point. We corrected most of the comments annotated in the PDF; however, we disagree in a few comments and also explained here our points of view.

1. It would be helpful to better explain why phylogenetic diversity is important - for Cuba & the studied environments - both in the introduction and the discussion.

R/ Agree. We added the following sentence in the introduction: “Phylogenetic diversity adds an evolutionary perspective to the biodiversity patterns …”. In the discussion and conclusions, we reinforced the component of phylogenetic diversity.

2. Introduction: it needs to focus more on the problems that this paper is addressing, the Cuban archipelago and the gap in knowledge that this work fills.

R/ Agree. We rephrased the paragraph devoted to the studies done in Cuba and highlighted the knowledge gaps.

3. Figure 9: please change the position of the text so that the letters don't overlap.

R/ Agree. We already moved the labels, but we wrongly uploaded an old version of this figure. We now upload the right version.

4. Figures 6, S1, S2 and S4: "sp." should not be in italics

R/ Agree. We fixed the issues.

5. Paragraph starting at line 594: what is the relation between the information from the literature and the present work?

R/ Agree. We modified the paragraph.

6. Lines 638-646: please rephrase for clarity

R/ Agree. We improved the wording.

7. Time-averaging (lines 649-666): this part can be reduce, the information on the subject can be provided in the references used; it would be better to focus on the time-averaging of the studied material.

R/ We agree. The two paragraphs were modified and fused.

8. Discussion: refer to the results and the figures that are the main focus of the paper.

R/ Agree. We added references to tables and figures where necessary.

9. Conclusions: the section can start with a sentence on the present work, so as to present a global view. The section should highlight the importance of the work, what new it brings rather than summarizing the main points of the results and discussion.

R/ Agree. We modified the conclusions, removing the repetition of results and highlighting the importance of our work.

10. Lastly, the English language should be revised throughout the document.

R/ Agree. We extensively revised the English usage.

Two comments in the annotated PDF that we disagreed with and further explain here our point of view.

- Please explain why you did this. Usually, for all bivalved organisms (bivalves, ostracods, brachiopods etc), we divide the total number by two, in order to have a better estimate of the real number of specimens. See Kowalewski et al. 2002 ([https://doi.org/10.1130/0016606(2002)114<0239:MHAOMM>2.0.CO;2](https://doi.org/10.1130/0016606(2002)114%3c0239:MHAOMM%3e2.0.CO;2)) "However, because an individual bivalve yields two skeletal elements (valves) and, thus, is twice as likely to be sampled as an individual gastropod, which produces only one shell, all analyses that compare abundance of bivalves and gastropods require a correction: The number of bivalve valves has to be divided by 2".

R/ The suggested approach is better used when the objective is an accurate assessment of abundance, usually to compare bivalves versus gastropods (as Kowalewski et al. 2002 stated). Our approach is also valid because the goal is to assess diversity and in much lesser extension to estimate the absolute abundance. This approach is also known as “Maximum number of individuals approach” (Gilinsky and Bennington 1994). Additionally, when dividing the number of counted valves by two, we would artificially inflate those diversity metrics that depend on the abundance, for instance the rarefaction and the evenness. We consider that our approach is correct, and no change has been made.

Gilinsky, N. L., & Bennington, J. B. (1994). Estimating numbers of whole individuals from collections of body parts: a taphonomic limitation of the paleontological record. Paleobiology, 20(2), 245-258.

- better use the term sessile. Can you also please add the name(s) one or two species here? This can be done also for the other functional groups (chemosynthetic deposit feeders, herbivores etc.).

R/ We think that sedentary is more appropriate than sessile as they are not the same. We keep the category sedentary. However, we changed the term immobile × sessile, as indicated in another comment.

We also think that is not necessary to mention the names of the species. These names can be found in the S1 table. But more important when using the functional approach, we do not want to go back to the species identity, instead the objective is to find patterns based solely on the trait identity.
